# Cyclopropanation Using Electrons Derived from Hydrogen: Reaction of Alkenes and Hydrogen without Hydrogenation

**DOI:** 10.1021/jacsau.4c00098

**Published:** 2024-03-25

**Authors:** Seiji Ogo, Takeshi Yatabe, Keishi Miyazawa, Yunosuke Hashimoto, Chiaki Takahashi, Hidetaka Nakai, Yoshihito Shiota

**Affiliations:** † Department of Chemistry and Biochemistry, Graduate School of Engineering, Kyushu University, 744 Moto-oka, Nishi-ku, Fukuoka 819-0395, Japan; ‡ International Institute for Carbon-Neutral Energy Research (WPI Academy I2CNER), Kyushu University, 744 Moto-oka, Nishi-ku, Fukuoka 819-0395, Japan; § Center for Small Molecule Energy, Kyushu University, 744 Moto-oka, Nishi-ku, Fukuoka 819-0395, Japan; ∥ Department of Energy and Materials, Faculty of Science and Engineering, Kindai University, 3-4-1 Kowakae, Osaka 577-8502, Japan; ⊥ Institute for Materials Chemistry and Engineering, Kyushu University, 744 Moto-oka, Nishi-ku, Fukuoka 819-0395, Japan

**Keywords:** cyclopropanation, hydrogen activation, iridium catalyst, hydrogen energy carrier, electron storage catalyst

## Abstract

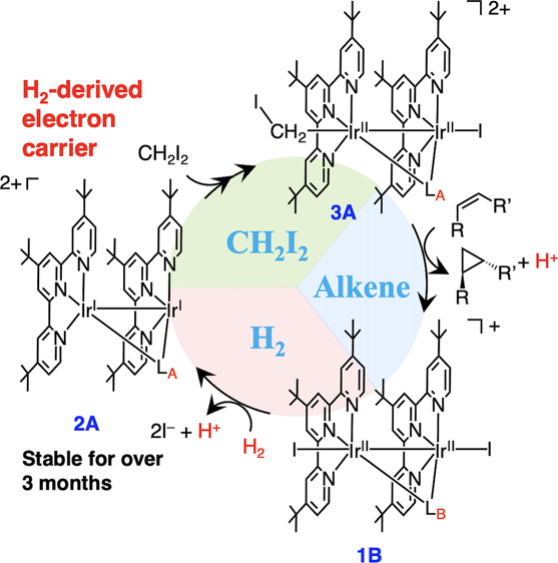

Have you ever imagined reactions of alkenes with hydrogen that result in anything other than hydrogenation or hydrogenative C–C coupling? We have long sought to develop not only hydrogenation catalysts that activate H_2_ as hydride ions but also electron transfer catalysts that activate H_2_ as a direct electron donor. Here, we report the reductive cyclopropanation of alkenes using an iridium electron storage catalyst with H_2_ as the electron source without releasing metal waste from the reductant. We discuss the catalytic mechanism with selectivity to give the *trans*-isomer. These findings are based on the isolation of three complexes and density functional theory calculations.

## Introduction

Reductive cyclopropanation, which converts alkenes to cyclopropanes, is an essential reduction reaction for the synthesis of many pharmaceuticals, pesticides, and natural products (Table S1).^1−16^ The Simmons–Smith reaction, which uses a zinc/copper reducing agent and diiodomethane as a CH_2_ source, is often used for such reductive cyclopropanation.^5,6^ However, metal waste from the reductant is a serious environmental problem. If it were possible to replace the Simmons–Smith reagent with hydrogen, we could greatly reduce the negative environmental impact of cyclopropanation.

**Figure 1:**
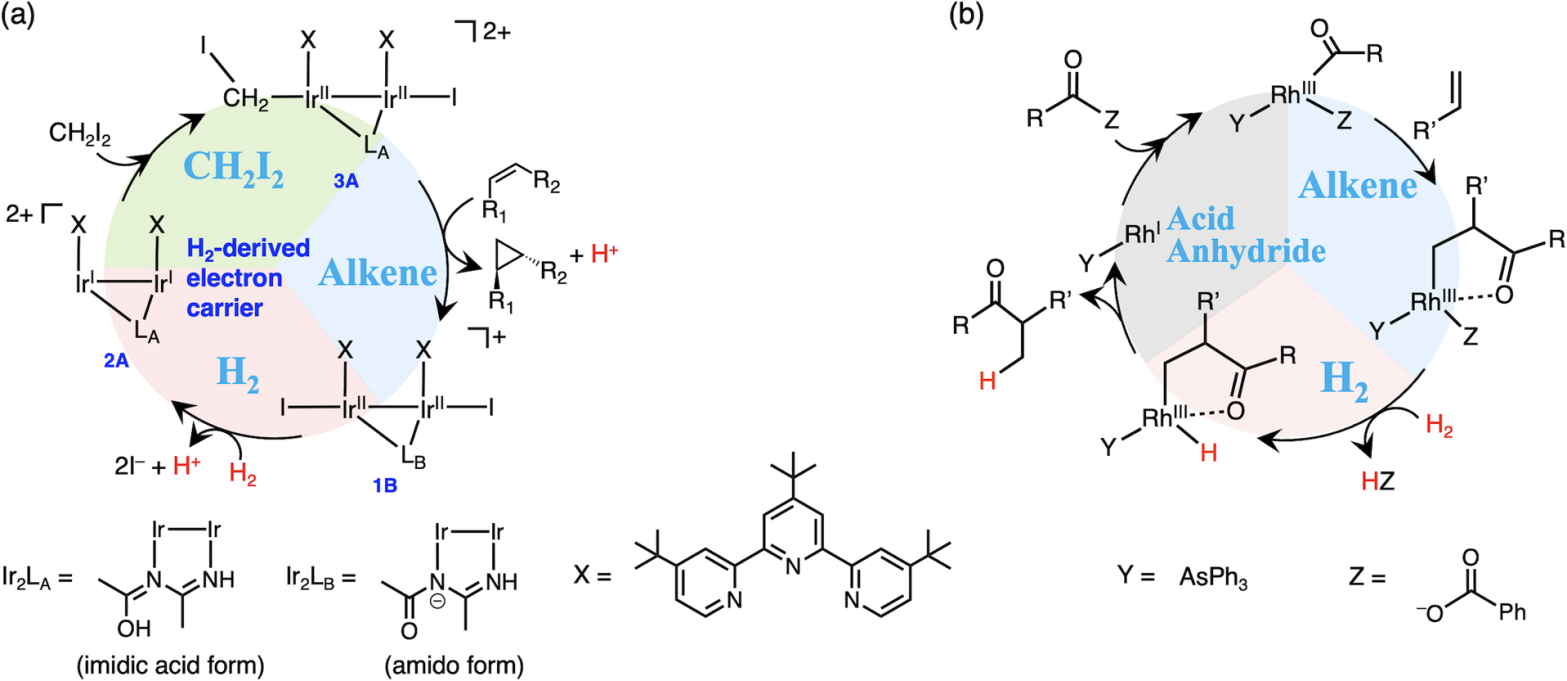
(a) Reductive cyclopropanation (this work). R_1_, R_2_ = alkyl or aryl. (b) Hydrogenative C–C coupling via Rh^I^ species in pioneering work by Krische and co-workers.^22^ R, R′ = aryl. Adapted with permission from ref (22). Copyright 2006 Wiley.

Hydrogen is a well-known reducing agent,^17^ but its application as a reductant in the presence of alkenes remains elusive because it simply adds across the double bonds and produces unwanted alkanes or hydrogenative C–C coupling products (Figure 1).^18−22^ Therefore, the ability to use H_2_ selectively as an electron source or as a hydride ion/atom source would be a great benefit to chemistry. This facility has already been realized in nature, however, and so we have chosen to emulate it.

Herein, we report a reductive cyclopropanation of alkenes using only H_2_ as the electron source for the first time. This reaction is based around an iridium catalyst that stores electrons from H_2_ before reaction with diiodomethane as the CH_2_ donor. In this paper, we discuss the mechanism of the reaction, based on the isolation of three complexes and density functional theory (DFT) calculations. These discussions also explain how the reaction proceeds via a stereoselective reductive cyclopropanation to give the *trans*-isomer, rather than stereospecific product as typically found in Simmons–Smith reactions.^23^

## Results and Discussion

**Figure 2:**
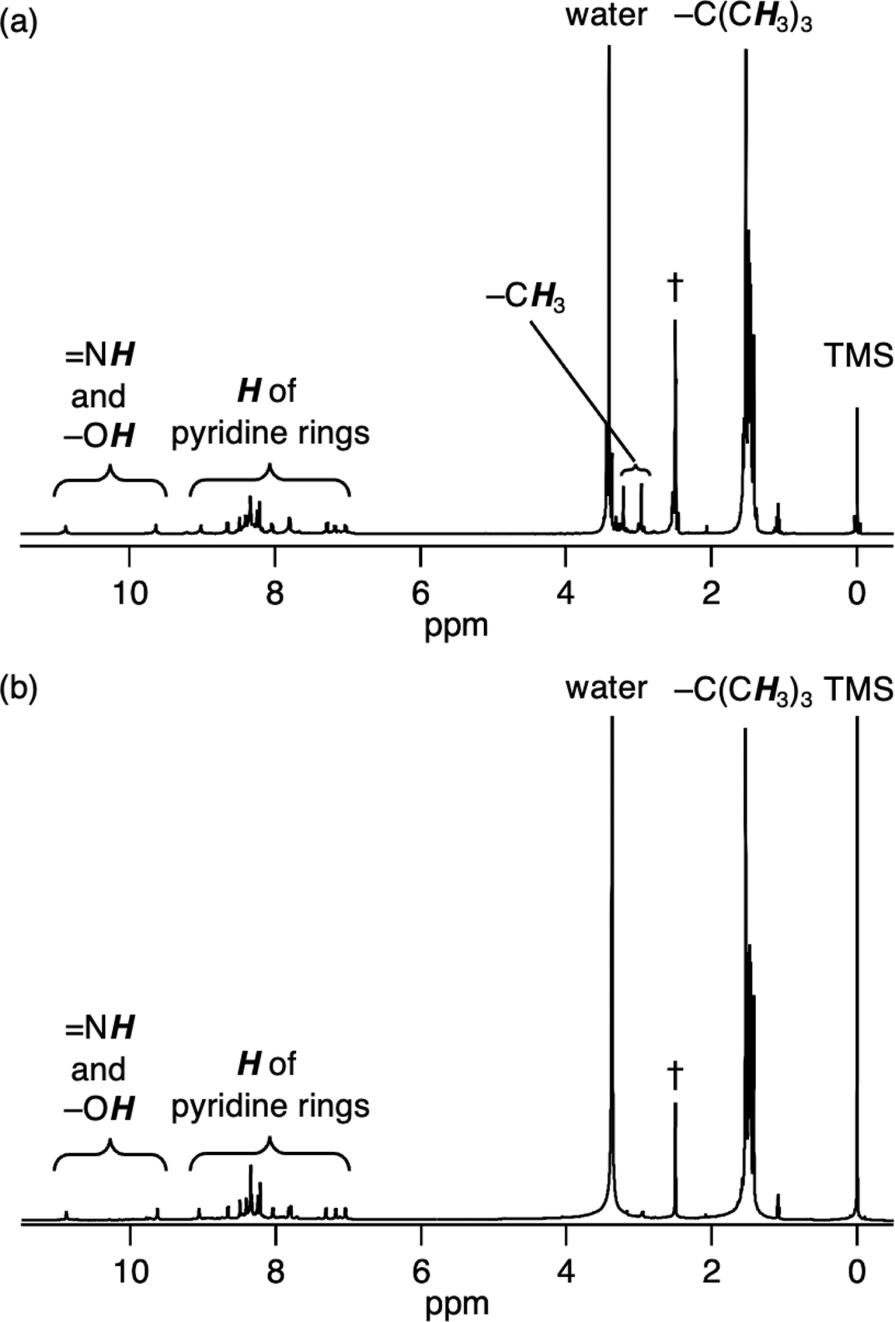
^1^H NMR spectra of (a) **1A** in DMSO-*d*_6_ and (b) D-labeled **1A** [Ir^II^_2_(Butpy)_2_(CD_3_(OH)C=N–C(CD_3_)=NH)(OH_2_)_2_](OTf)_4_ in DMSO-*d*_6_. Tetramethylsilane (TMS), reference with the methyl proton resonance set at 0.00 ppm. †: Peak of dimethyl sulfoxide.

**Figure 3:**
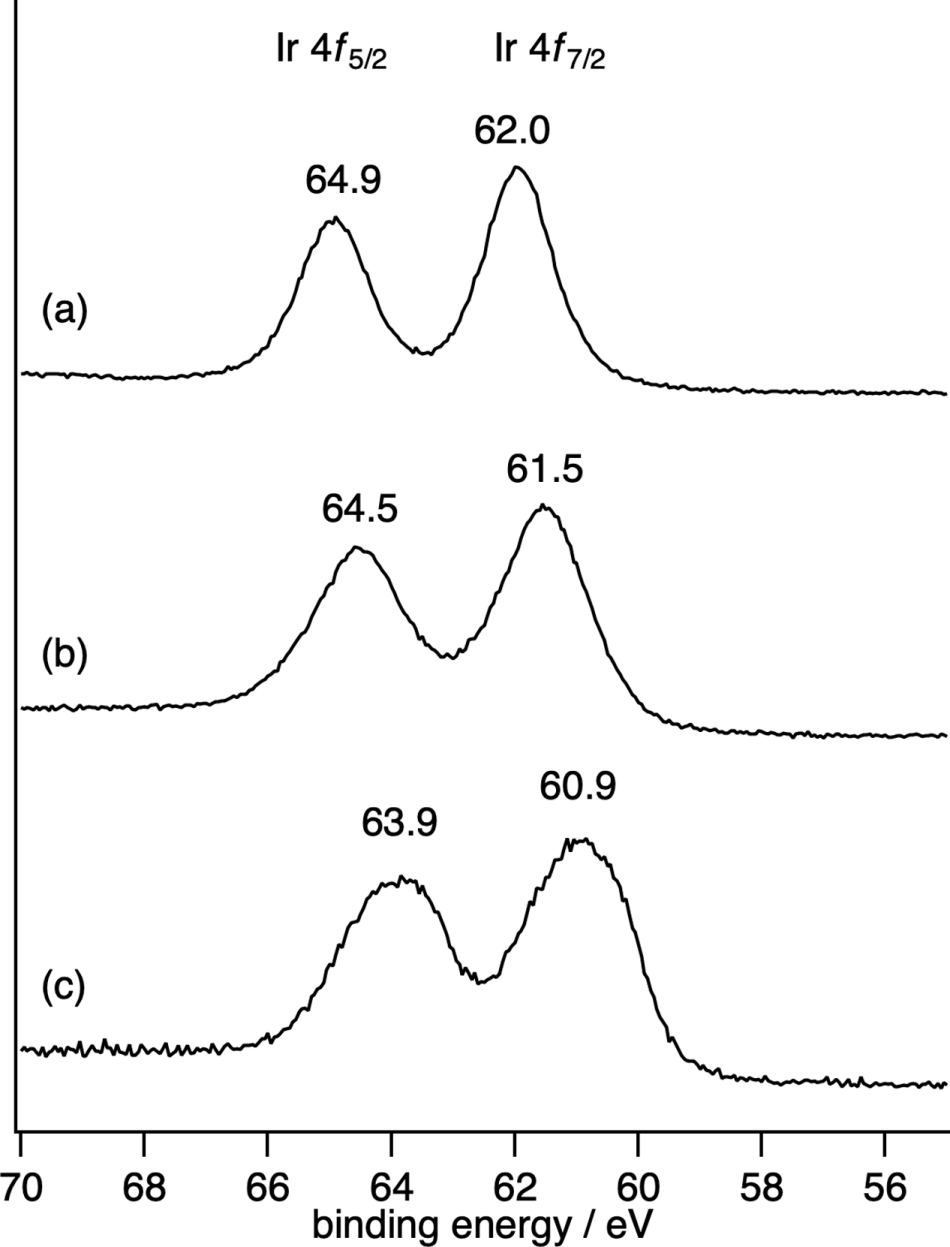
XPS spectra of (a) [Ir^III^(Butpy)(Cl)_3_], (b) **1A**, and (c) **2A** of the Ir 4*f* region.

An iridium dinuclear complex [Ir^II^_2_(Butpy)_2_(L_A_)(OH_2_)_2_](OTf)_4_ {[**1A**](OTf)_4_, Butpy = 4,4′,4″-tri*tert*-butyl-2,2′:6′,2″-terpyridine, L_A_ (imidic acid form) = CH_3_(OH)C=N–C(CH_3_)=NH, OTf = trifluoromethanesulfonate} was synthesized by the reaction of [Ir^I^(COD)Cl]_2_ (COD = 1,5-cyclooctadiene) with the Butpy and trifluoromethanesulfonic acid in CH_3_CN with 51% yield based on [Ir^I^(COD)Cl]_2_, which was characterized by nuclear magnetic resonance (NMR) spectroscopy (Figures 2 and S1), electrospray ionization–mass spectrometry (ESI-MS, Figure S2), infrared (IR) spectroscopy (Figure S3), X-ray photoelectron spectroscopy (XPS, Figure 3b), and elemental analysis. The ^1^H NMR spectrum of **1A** shows signals at 1.42–1.53 and 7.02–9.02 ppm from the Butpy, 2.97 and 3.21 ppm from −CH_3_ groups of L_A_, and 9.65 and 10.88 ppm from O–H and N–H groups of L_A_ (Figure 2a). A positive-ion ESI-mass spectrum shows the prominent peak at *m*/*z* 717.8 {relative intensity (*I*) = 100% in the range of *m*/*z* 100–2000}, which has a characteristic isotopic distribution that matches well with the calculated isotopic distribution for [**1A**+OTf–2H_2_O–2H]^2+^ (Figure S2). An XPS spectrum of **1A** shows that binding energies of Ir 4*f*_7/2_ and Ir 4*f*_5/2_ peaks are 61.5 and 64.5 eV, respectively (Figure 3b), which were lower than those of Ir^III^ complex [Ir^III^(Butpy)(Cl)_3_] and higher than those of Ir^I^ complex (Figure 3a,c) (vide infra).

To investigate the origin of L_A_, an isotope-labeled experiment using CD_3_CN for the synthesis of **1A** was conducted. The ^1^H NMR spectrum of the D-labeled **1A** [Ir^II^_2_(Butpy)_2_(CD_3_(OH)C=N–C(CD_3_)=NH)(OH_2_)_2_](OTf)_4_, synthesized by using CD_3_CN instead of CH_3_CN, shows that the signals of the methyl group of L_A_ at 2.97 and 3.21 ppm disappear (Figure 2b). This result indicates that the bridging ligand L_A_ forms by the hydrolytic coupling of two molecules of acetonitrile as solvent, as reported for the Cu and Ru complexes.^24−26^

**Scheme 1:**
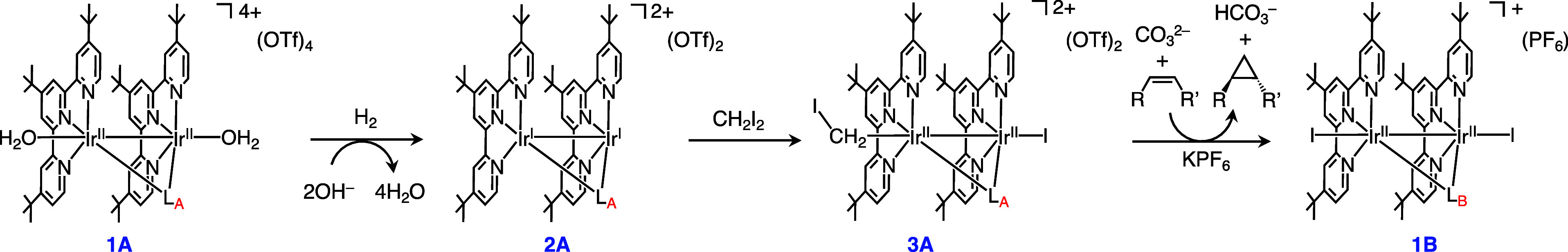
Reaction Scheme

Complex **1A** reacts with H_2_ (0.1–0.8 MPa) in water (pH 9) at room temperature to form an Ir^I^ complex [Ir^I^_2_(Butpy)_2_(L_A_)](OTf)_2_ {[**2A**](OTf)_2_} (H_2_-derived electron carrier, Scheme 1) with the yield of 68% based on **1A**. The extraction of the two electrons from H_2_ is facilitated by water as a Lewis base in stoichiometric reactions and by CO_3_^2–^ as a Lewis base in catalytic reactions.^27,28^ The two electrons are transferred to two Ir^II^ centers to provide low-valent Ir^I^ centers. The Ir^I^ centers are stabilized by the Butpy ligand, in which the ^t^Bu moiety is electron-donating and the tpy moiety is electron-withdrawing.

**Figure 4:**
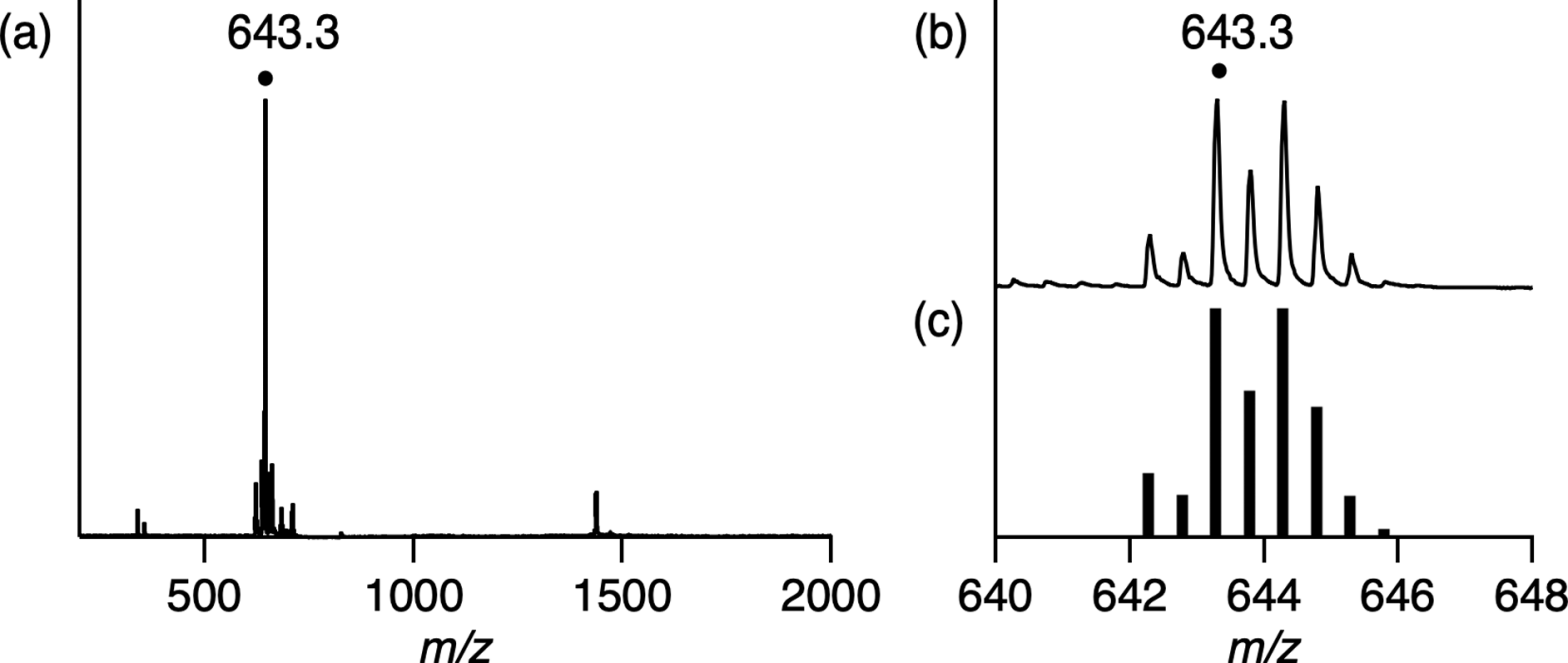
(a) Positive-ion ESI-mass spectrum of **2A** in methanol. The signal at *m*/*z* 643.3 corresponds to [**2A**]^2+^. (b) Signal at *m*/*z* 643.3 for [**2A**]^2+^. (c) Calculated isotopic distribution for [**2A**]^2+^.

Complex **2A** is stable in the solid state for more than 3 months and characterized by UV–vis–NIR absorption spectroscopy (Figure S4), ESI-MS (Figure 4), IR spectroscopy (Figure S5), ESR spectroscopy (Figure S6), XPS (Figure 3c), and elemental analysis. As shown in Figure S4, **2A** shows an absorption band at 550–1200 nm, which can be observed in the absorption spectra of the reported Ir^I^ polypyridyl complexes.^29^ A positive-ion ESI-mass spectrum of **2A** shows the prominent peak at *m*/*z* 643.3 {*I* = 100% in the range of *m*/*z* 100–2000} that corresponds to [**2A**]^2+^ and a characteristic isotopic distribution that matches well with the calculated isotopic distribution (Figure 4). The ESR spectrum of **2A** shows diamagnetism (Figure S6). The XPS spectrum of **2A** shows that binding energies of the Ir 4*f*_7/2_ and Ir 4*f*_5/2_ peaks are 60.9 and 63.9 eV, respectively (Figure 3c). These peaks were lower than those of **1A**, but similar to those found in the reported Ir^I^ complexes.^30^ These results show that the oxidation state of the two Ir metal centers in **2A** is monovalent.

**Figure 5:**
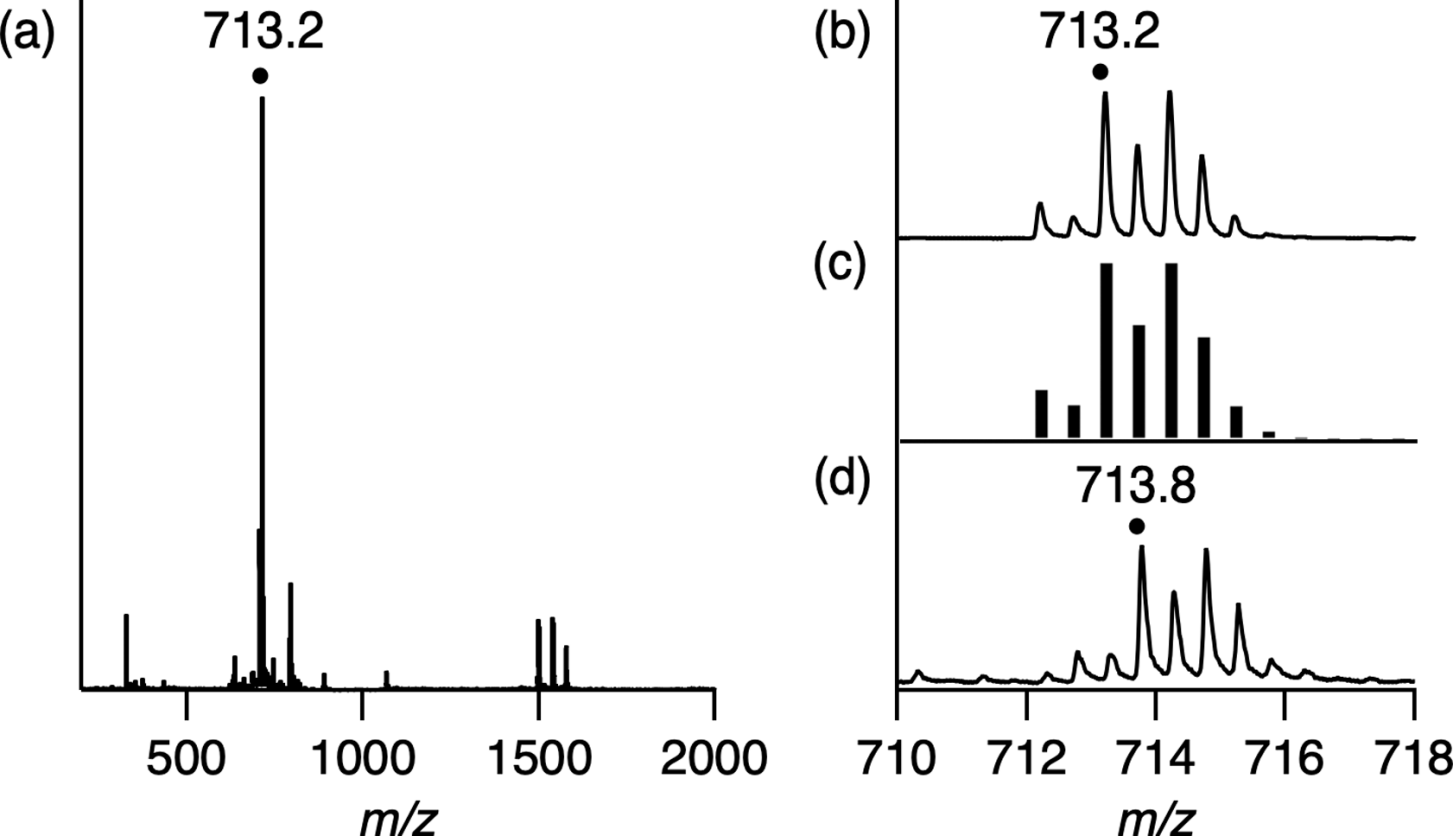
(a) Positive-ion ESI-mass spectrum of **3A** in CH_3_CN and methanol. The signal at *m*/*z* 713.2 corresponds to [**3A**–I–H]^2+^. (b) Signal at *m*/*z* 713.2 for [**3A**–I–H]^2+^. (c) Calculated isotopic distribution for [**3A**–I–H]^2+^. (d) Positive-ion ESI-mass spectrum of ^13^C-labeled **3A** in CH_3_CN and methanol. The signal at *m*/*z* 713.8 corresponds to [^13^C-labeled **3A**]^2+^_._

Complex **2A** reacts with CH_2_I_2_ at room temperature to afford an Ir^II^ carbenoid complex [Ir^II^_2_(Butpy)_2_(CH_2_I)(L_A_)(I)](OTf)_2_ {[**3A**](OTf)_2_} (Scheme 1), which was characterized by ESI-MS (Figure 5), NMR (Figures S7 and S8), and ESR spectroscopy (Figure S9). The reaction of **2A** with CH_2_I_2_ in acetone was monitored by UV–vis–NIR absorption spectroscopy (Figure S10). The absorption band at over 400 nm of **2A** decreases by the addition of CH_2_I_2_. A positive-ion ESI-mass spectrum of **3A** shows a prominent peak at *m*/*z* 713.2, which has an isotopic distribution corresponding to the calculated isotopic distribution of [**3A**–I–H]^2+^ (Figure 5). Isotope-labeled experiment for the reaction of **2A** with ^13^CH_2_I_2_ instead of ^12^CH_2_I_2_ showed an isotopic substitution of ^12^C by ^13^C in the carbenoid ligand resulted in a signal shift from 713.2 to 713.8 (Figure 5d). The ^1^H NMR spectrum of **3A** shows a signal at 5.31 ppm derived from the carbenoid moiety (Figure S7a). The ^1^H NMR spectrum of **3A** labeled with ^13^C showed a signal at 5.31 ppm that splits into a doublet with a coupling constant of 153 Hz due to ^13^C–^1^H coupling (Figure S7b). A ^13^C NMR spectrum of **3A** shows the signal at −23.0 ppm derived from C atom of the carbenoid group, which is similar to those found in the reported carbenoid complexes (Figure S8).^31^ The ESR spectrum of **3A** shows diamagnetism (Figure S9). These results indicate that the oxidative addition of CH_2_I_2_ to Ir^I^ complex **2A** yields Ir^II^ carbenoid complex **3A**.

**Figure 6:**
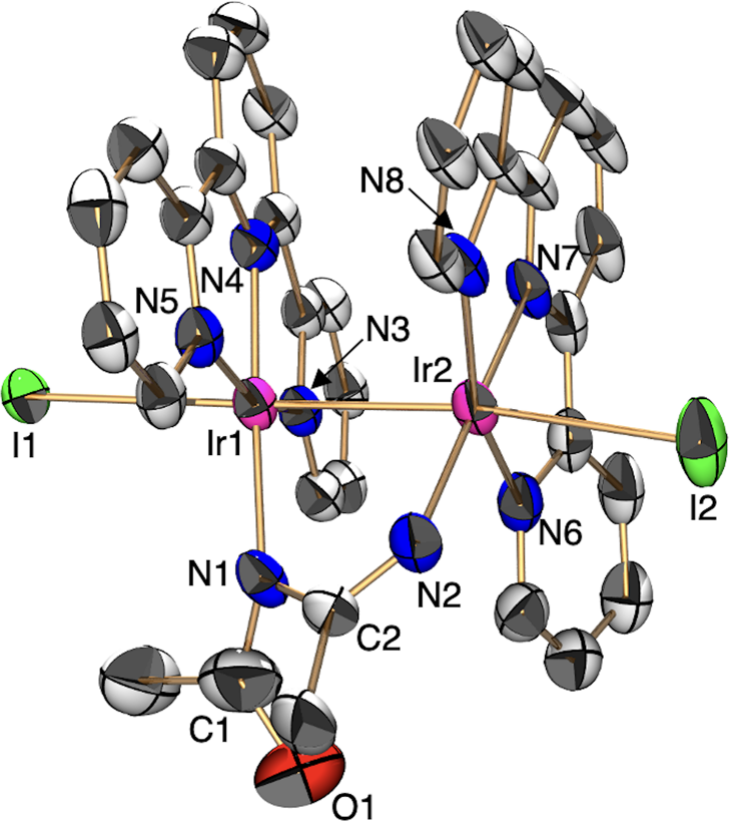
ORTEP drawing of **1B** with ellipsoids at 50% probability. ^t^Bu groups, hydrogen atoms, and conteranions are omitted for clarity. Selected interatomic distances (*l*/Å): Ir1–Ir2 = 2.6689(3), Ir1–N1 = 2.119(5), Ir2–N2 = 2.060(4), C1–O1 = 1.289(11), C1–N1 = 1.428(10), C2–N1= 1.351(8), C2–N2 = 1.269(7). In the catalytic cycle, the bridging ligand of complex **1A** changes from the imidic acid form (**1A**) to the amido form (**1B**) in the presence of potassium carbonate.

**Figure 7:**
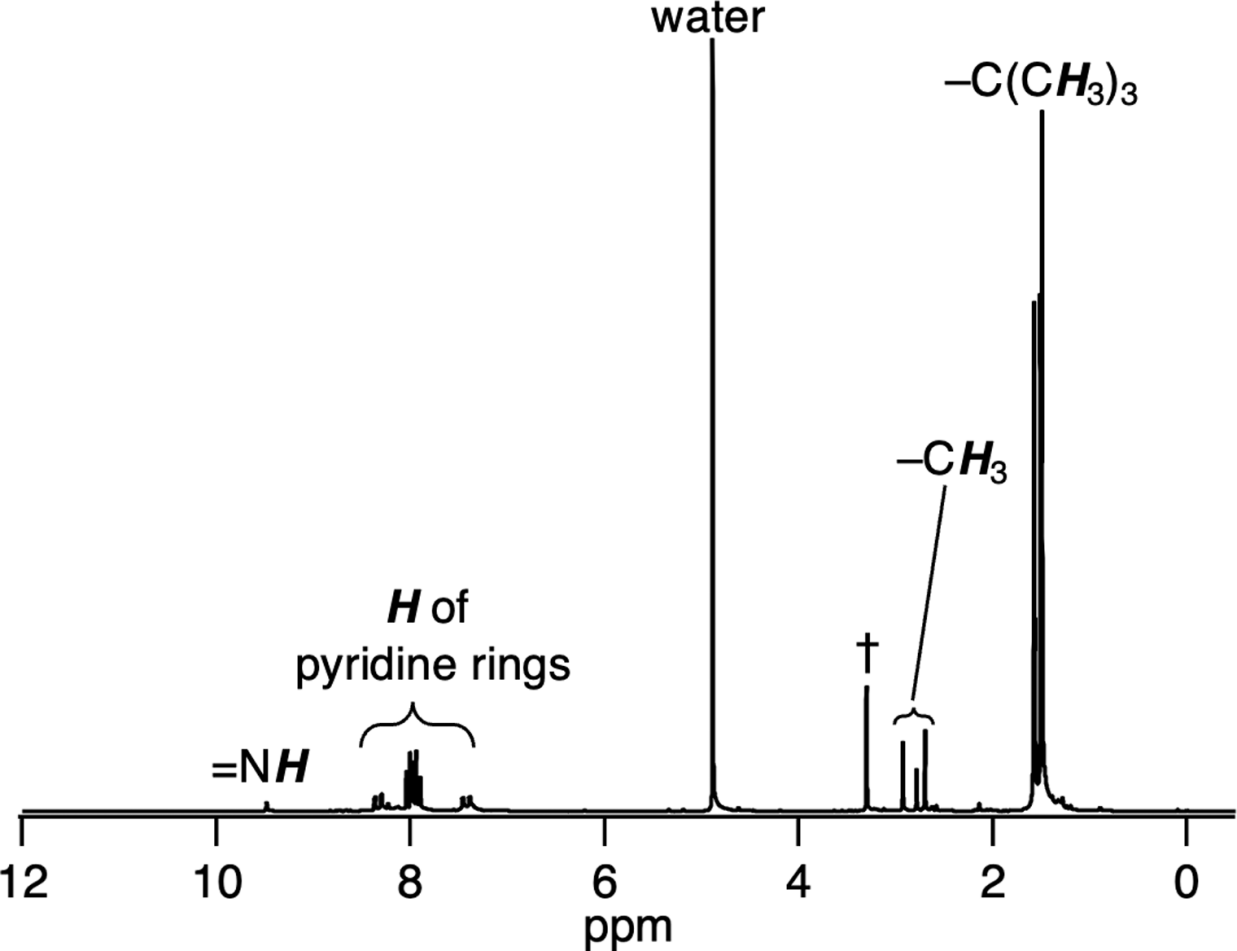
^1^H NMR spectrum of **1B** in CD_3_OD. †: Peak of methanol.

Carbenoid complex **3A** reacts with styrene in the presence of potassium carbonate with KPF_6_ to yield Ir^II^ diiodide complex [Ir^II^_2_(Butpy)_2_(L_B_)(I)_2_](PF_6_) {[**1B**](PF_6_), L_B_ (amido form) = CH_3_(C=O)N–C(CH_3_)=NH} and phenylcyclopropane (Scheme 1). Characterization of **1B** was conducted by X-ray analysis (Figure 6), ^1^H NMR spectrometry (Figure 7), ESI-MS (Figure S11), and elemental analysis. The isolation yield of **1B** was 38% based on **2A**. A single crystal of **1B** was obtained from the methanol/acetone solution of **1B** diffused by diethyl ether. An Oak Ridge thermal ellipsoid plot (ORTEP) drawing of **1B** shows that each Ir metal center adopts the distorted octahedral geometry with the Butpy, two iodide ions, and L_B_ (Figure 6). The Ir1–Ir2 bond distance {2.6689(3) Å} is comparable to those of the reported Ir dimer complexes {2.6584(5)–2.8703(8) Å}.^32^ The distance of C1=O1 bond {1.289(11) Å} is within the range of C=O double bond distance.^33^ The distances of C2–N1 {1.351(8) Å} and C2–N2 {1.269(7) Å} bonds are similar to those of the C–N single bond and the C=N double bond, respectively,^33^ which suggests that the negative charge is localized to the N1 atom. The ^1^H NMR spectrum of **1B** shows no O–H signal of the bridging ligand found in **1A** (Figure 7). Thus, the reaction of **3A** with alkene under basic conditions led to form L_B_ by the deprotonation of L_A_ in **1A**. A positive-ion ESI-mass spectrum of **1B** shows the prominent peak at *m*/*z* 1539.3, which has isotopic distribution corresponding to the calculated isotopic distribution of [**1B**]^+^ (Figure S11). The formed phenylcyclopropane was characterized by gas chromatography–mass spectrometry (GC-MS) and quantified by ^1^H NMR spectroscopy with the yield of 47% based on **3A**. These results indicate that the carbene transfer reaction clearly occurs from carbenoid complex **3A**, which is produced by the electrons from H_2_.

**Figure 8:**
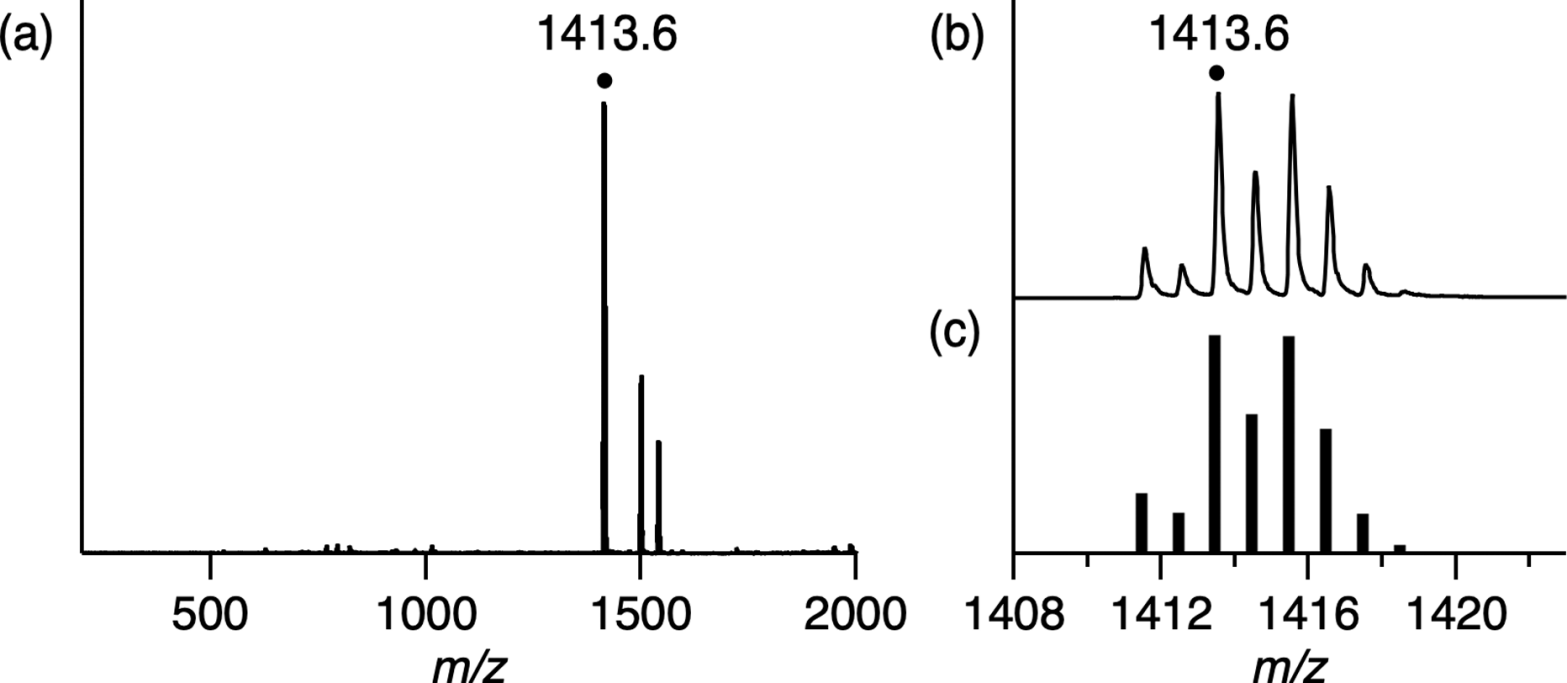
(a) Positive-ion ESI-mass spectrum of the reaction solution of **1B** with H_2_ in propanol and methanol. The signal at *m*/*z* 1413.6 corresponds to [**2A**+I]^+^. (b) Signal at *m*/*z* 1413.6 for [**2A**+I]^+^. (c) Calculated isotopic distribution for [**2A**+I]^+^.

To confirm the regeneration of Ir^I^ electron storage catalyst **2A** under the conditions without K_2_CO_3_, the reaction of **1B** with H_2_ was monitored by ESI-MS (Figure 8). The ESI-mass spectrum of the reaction solution of **1B** with H_2_ shows the prominent peak at *m*/*z* 1413.6, which has an isotopic distribution corresponding to the calculated isotopic distribution of [**2A**+I]^+^, suggesting that **1B** extracts electrons from H_2_ to regenerate the electron storage complex **2A**.

Having established the stepwise reactions, we investigated the catalytic cyclopropanation of various alkenes with CH_2_I_2_ by **1A** in the presence of potassium carbonate under a H_2_ atmosphere (0.5 MPa) in CD_3_CN at 80 °C (Tables 1 and S2). It is noteworthy that there are no hydrogenation products of alkene as a byproduct, indicating that the Ir-based electron storage catalyst uses H_2_ as an electron source, but not as a hydride source.

**Table 1:**
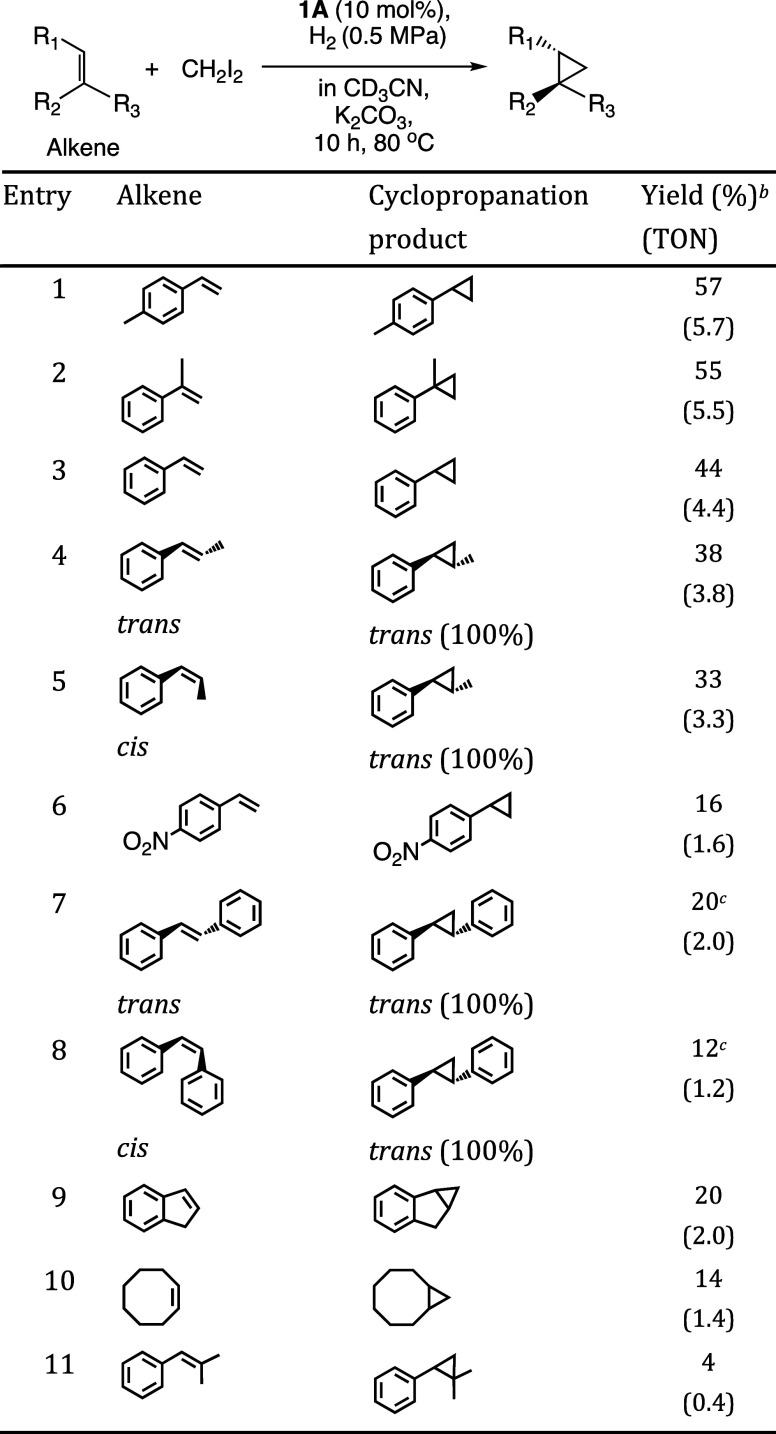
Catalytic Reductive Cyclopropanation of Alkene with CH_2_I_2_ under H_2_^a^

a Reaction conditions: complex **1A** (1.15 μmol), alkene (11.5 μmol), CH_2_I_2_ (0.42 mmol), K_2_CO_3_ (0.23 mmol), CD_3_CN (500 μL), H_2_ (0.5 MPa), 10 h, 80 °C. The reaction starts with **1A**, which is easier to synthesize than **1B**.

b Yields of the products of cyclopropanation and hydrogenation were determined by ^1^H NMR without purification because of the low boiling points of the products. Turnover numbers (TONs, mol of cyclopropane compounds/mol of **1A**) based on complex **1A**.

c Isolated yield measured by the balance of cyclopropanation of *trans*- or *cis*-stilbene giving the nonvolatile product, *trans*-1,2-diphenylcyclopropane, was 19% or 10%.

The formed cyclopropane compounds were quantified by ^1^H NMR spectroscopy. The yields of cyclopropanation for aromatic alkenes (entries 1–9) were better than those of aliphatic alkenes (entry 10). The maximum turnover number (TON) was determined to be 5.7 for 4-methylstyrene (entry 1). Most of the remaining material is an unreacted starting alkene. No cyclopropane compounds were formed without **1A** or H_2_ (Table S2 entries 2 or 3), and a hydrogenation product was formed without CH_2_I_2_ (Table S2 entry 4). While the Simmons–Smith reaction using Zn-carbenoid gives stereospecific products,^5,6,23^ the *trans*-products were obtained in the case of *cis*-β-methylstyrene and *cis*-stilbene (entries 5 and 8). The catalytic cyclopropanation of styrene using isotope-labeled ^13^CH_2_I_2_ gives ^13^C-labeled phenylcyclopropane, as detected by GC-MS (Figure S12). The addition of 200 equiv of radical trapping reagent (*tert*-butyl-α-phenylnitrone) to the catalytic reaction gives only trace amount of phenylcyclopropane. The hydrogenation of alkene was hardly promoted by the addition of water to the reaction solution (Table S2 entry 8). In contrast to **1A**, the hydrogenation catalyst Pd/C gives hydrogenation products under the same catalytic conditions without cyclopropanation products (Table S3).

A positive-ion ESI-mass spectrum of the catalytic reductive cyclopropanation of alkene (11.5 μmol) with **1A** (1.15 μmol), CH_2_I_2_ (0.42 mmol), K_2_CO_3_ (0.23 mmol), and H_2_ (0.5 MPa) in CD_3_CN (500 μL) at 80 °C shows a signal at *m*/*z* 727.3 (*I* = 100% in the range *m*/*z* 100–2000), which has a characteristic distribution that matches well with the calculated isotopic distribution of [**1B**–I–H]^2+^ (Figure S13) (eq 1).
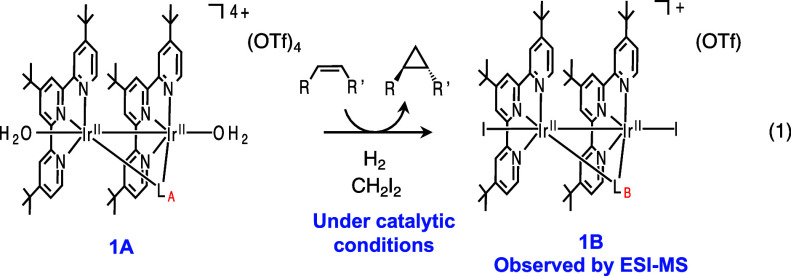
1

The catalytic reaction performed by using the mononuclear [Ir^III^(Butpy)(Cl)_3_] as a catalyst shows a low TON as 0.2 for the cyclopropanation of styrene (Table S2 entry 5). This result indicates that the dinuclear Ir structure is crucial for the cyclopropanation with H_2_.^34^

To provide deeper insights into the reaction pathways, including intermediates for the reactions of **2A** with CH_2_I_2_, **3A** with *cis*-stilbene, and **1B** with H_2_, DFT calculations were performed (Tables S5–S17). A free energy profile, using the B3LYP functional, is presented in Figures S14–S16.

The formation of the carbenoid complex **3A** by the oxidative addition of CH_2_I_2_ to **2A**, via transition state **TS1** (barrier height: 26.0 kcal/mol), is a thermodynamically favored reaction (−22.3 kcal/mol) (Figure S14). Complex **3A** is a singlet state with a metal–metal bond between the two Ir^II^ metal centers. Two electrons from each Ir^I^ metal center are used for the oxidative addition of CH_2_I_2_, as reported for the formation of other Ir^II^ dimer carbenoid complexes.^35^

The formation of **1B** and cyclopropane from **3A** and *cis*-stilbene, via transition states **TS2A** and **TS3A**, is also a thermodynamically favored reaction (−98.5 kcal/mol) (Figure S15). First, the reaction of **3A** with *cis*-stilbene yields a radical intermediate (**INT2A**) that cleaves the Ir–C bond via the transition state **TS2A** (barrier height: 34.8 kcal/mol). The radical intermediate **INT2A** is represented as Ir^I^ with the radical formally at the terminal carbon atom and is in the open-shell singlet state. The C(benzyl)–C(benzyl) bond in the radical intermediate is a partial double bond and rotates freely. The **INT2A** gives an imidic acid type species (**INT3A**) by the transfer of an iodide ion to the Ir metal center via transition state **TS3A** and gives the *trans-*cyclopropane product. The energy barrier of **TS3A** with the *trans* conformation to the phenyl groups (4.7 kcal/mol) is lower than that with the *cis* conformation (11.9 kcal/mol) due to the steric repulsion of the phenyl groups in the dissociated I–CH_2_–C(C_6_H_5_)–C(C_6_H_5_)· radical. The **INT3A** reacts with the Lewis base K_2_CO_3_ to give the amide type **1B** (−98.5 kcal/mol).^36,37^

The recovery of **2A** by the reaction of **1B** with H_2_ is a thermodynamically favored reaction (−46.3 kcal/mol) (Figure S16).^36,37^ These calculation results are consistent with the results from the radical trapping experiment (vide supra) and the stereoselectivity of the cyclopropanations (Table 1, entries 5 and 8). Thus, the reaction of carbenoid **3A** with alkenes, unlike the concerted [2 + 1] fashion of the Simmons–Smith reaction, forms radical intermediates and gives the thermodynamically stable *trans*-products. A similar *trans* selectivity of cyclopropanation with alkenes has been previously reported using the Ru photocatalyst (Table S1), and this study has also proposed iodomethyl radical intermediates.^11^

**Figure 9:**
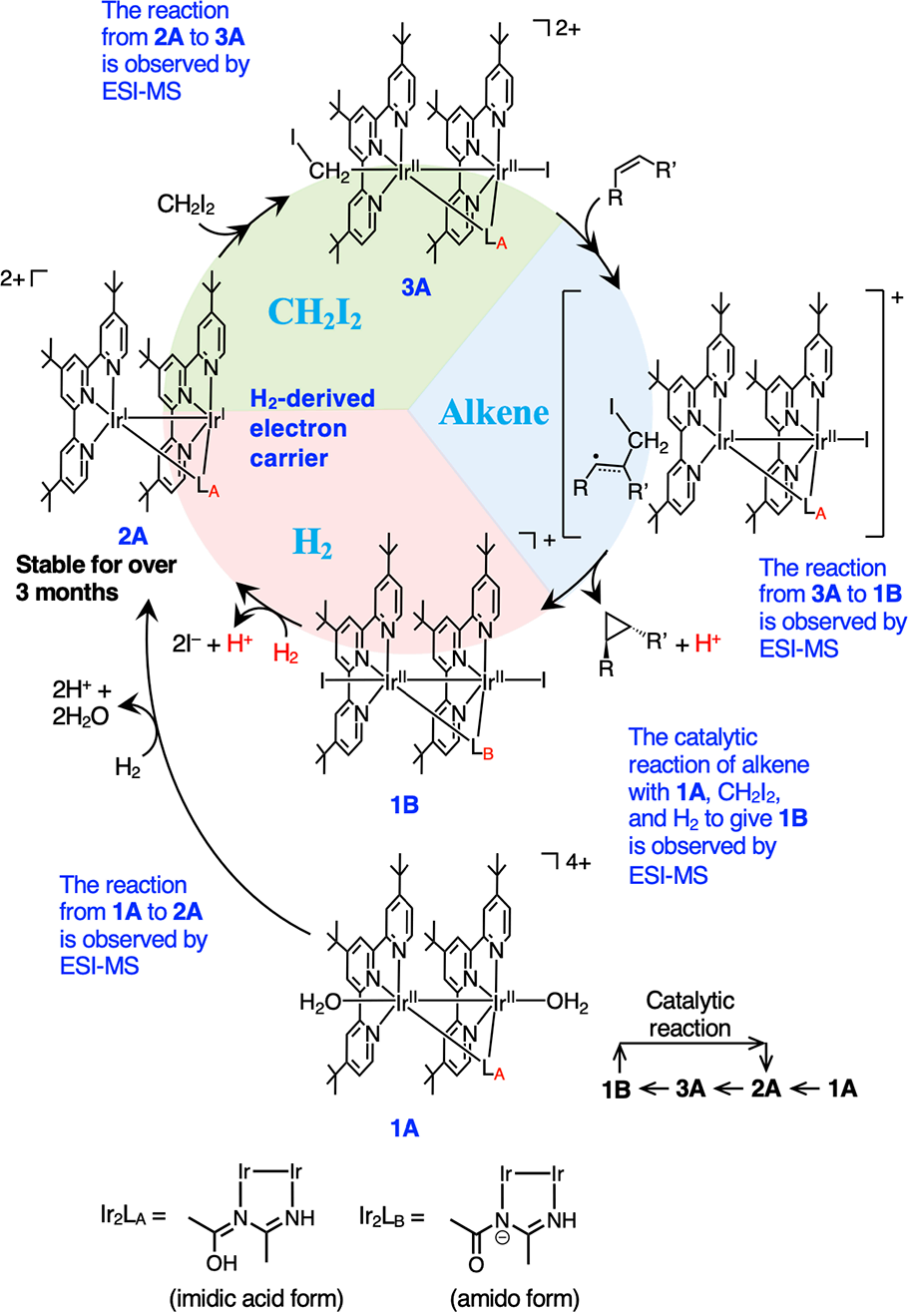
Proposed reaction mechanism of the reductive cyclopropanation of alkene with CH_2_I_2_ under H_2_. L_A_: CH_3_(OH)C=N–C(CH_3_)=NH. L_B_: CH_3_(C=O)N–C(CH_3_)=NH. The reaction starts with **1A**, which is easier to synthesize than **1B**. In the catalytic cycle, the bridging ligand of **1A** changes from the imidic acid form (**1A**) to the amido form (**1B**) in the presence of K_2_CO_3_. Catalytic reaction: **1A** → **2A** → **3A** → **1B** → **2A**.

Based on the above results and their analysis, we propose the full reaction mechanism, as shown in Figure 9. The reaction of starting catalyst **1A**, or **1B**, with H_2_ produces electron storage catalyst **2A**. Complex **2A** then cleaves the C–I bond of CH_2_I_2_ to yield carbenoid complex **3A**, which is followed by a carbene transfer to the alkene to produce the corresponding cyclopropane compounds and return the starting Ir^II^ complex **1B**.

## Conclusions

In conclusion, the reductive cyclopropanation of alkenes using H_2_ as an electron source was achieved for the first time using an Ir electron storage catalyst. The cyclopropanation yields only *trans-*products and proceeds via a stepwise radical mechanism, as elucidated by experimental and theoretical studies. Although the obtained TON is low, stoichiometric metal waste was avoided using this methodology, which points to a more environmentally friendly future for the production of cyclopropanes. This success therefore points the way to giving an old reducing agent new potential in organic synthesis.

## Methods

### Materials and Methods

All experiments were carried out under a N_2_ atmosphere using standard Schlenk techniques unless stated in a glovebox. Acetonitrile was distilled over CaH_2_ under a N_2_ atmosphere. H_2_ (99.9999%) was purchased from Sumitomo Seika Chemical Co., Ltd., sodium iodide, potassium carbonate, potassium hexafluorophosphate, potassium acetate, 4-methylstyrene, and indene were purchased from FUJIFILM Wako Pure Chemical Corporation, chloro(1,5-cyclooctadiene)iridium(I) dimer, trifluoromethanesulfonic acid, diiodomethane, styrene, α-methylstyrene, *trans*-β-methylstyrene, *cis*-β-methylstyrene, 4-nitrostyrene, *trans*-stilbene, *cis*-stilbene, cyclooctene, 2-methyl-1-phenylpropene, and *N*-*tert*-butyl-α-phenylnitrone (PBN) were purchased from Tokyo Chemical Industry Co. Ltd., 4,4′,4″-tri*tert*-butyl-2,2′:6′,2″-terpyridine (Butpy) was purchased from Aldrich, and CD_3_OD, CDCl_3_, CD_3_CN, DMSO-*d*_6_, and ^13^C-labeled diiodomethane were purchased from Cambridge Isotope Laboratories, Inc. These reagents were used without further purification. [Ir^III^(Butpy)(Cl)_3_] was synthesized by the literature method.^38^

ESI-MS data were obtained by a JEOL JMS-T100LC AccuTOF. Proton nuclear magnetic resonance (^1^H NMR) and ^13^C NMR spectra were recorded on a Bruker Advance III 600 FT-NMR spectrometer. Chemical shifts were referenced to tetramethylsilane (TMS) (δ = 0.00 ppm) in CD_3_OD and DMSO-*d*_6_ and to protio solvent impurities (δ = 2.05 ppm) in acetone-*d*_6_ for ^1^H NMR and to protio solvent impurities (δ = 118.3 or 29.8 ppm) in CD_3_CN or acetone-*d*_6_ for ^13^C NMR. Ultraviolet–visible–near-infrared (UV–vis–NIR) spectra were recorded on a JASCO V-670 UV–vis-NIR spectrophotometer. XPS spectra were recorded on an ULVAC PHI 5000 VersaProbe II system with an Al anode X-ray source. Binding energies were calibrated by the C 1s peak of carbon at 284.5 eV.^39^ GC-MS data were recorded on an Agilent 7890B GC coupled to a 5977B MSD. All catalytic and stoichiometric reactions using H_2_ were carried out in a high-pressure glass cylinder (95 mL, TAIATSU TECHNO).

### [Ir^II^_2_(Butpy)_2_(L_A_)(OH_2_)_2_](OTf)_4_ {[1A](OTf)_4_, L_A_ = CH_3_(OH)C=N–C(CH_3_)=NH, OTf = Trifluoromethanesulfonate}

In the glovebox, a mixture solution of Butpy (200 mg, 0.498 mmol) in tetrahydrofuran (5.0 mL) and chloro(1,5-cyclooctadine)iridium(I) dimer (167 mg, 0.249 mmol) in acetonitrile (20 mL) was stirred at room temperature for 8 h. The solvent was removed under reduced pressure, and the residue was washed by *n*-hexane. The resulting solid was dissolved in acetonitrile (5.0 mL) and water (50 μL). Trifluoromethanesulfonic acid (330 μL) was added to the solution with stirring, and the resulting solution was stirred at room temperature for 15 min under a N_2_ atmosphere. The solvent was removed under reduced pressure, and the residue was dissolved in methanol (5.0 mL). Diethyl ether (10 mL) and *n*-hexane (25 mL) were added to the solution to yield dark-red oil, and the solvent was removed by decantation followed by the evaporation. The residue was dissolved in acetonitrile (5.0 mL), and diethyl ether (50 mL) was added to the acetonitrile solution to precipitate a red-brown solid. The solvent was removed by decantation. After the red-brown solid was dried in vacuo, the residue was recrystallized in methanol solution (9.0 mL) diffused by diethyl ether for 2 days at 25 °C. Red-black crystals were collected by filtration and dried in vacuo {yield: 51% based on chloro(1,5-cyclooctadine)iridium(I) dimer}. ESI-MS (in methanol): *m*/*z* = 717.8 {[**1A**+OTf–2H_2_O–2H]^2+^, relative intensity (*I*) = 100% in the range of *m*/*z* 100 to 2000}.^1^H NMR (600 MHz, in DMSO-*d*_6_, referenced to TMS): δ 10.9 and 9.65 (s, 2H, −N*H* and −O*H*), 9.02 (s, 1H, −C*H*), 8.66 (s, 1H, −C*H*), 8.49 (d, 1H, −C*H*), 8.41 (d, 1H, −C*H*), 8.34–8.36 (m, 3H, −C*H*), 8.25 (s, 1H, −C*H*), 8.22 (s, 2H, −C*H*), 8.04 (d, 1H, −C*H*), 7.79–7.81 (m, 2H, −C*H*), 7.29 (d, 1H, −C*H*), 7.18 (d, 1H, −C*H*), 7.03 (d, 1H, −C*H*), 3.21 (s, 3H, −C–C*H*_3_), 2.97 (s, 3H, −C–C*H*_3_), 1.53–1.42 {s, 54H, −C(C*H*_3_)_3_}. ^13^C NMR (150 MHz, in acetone-*d*_6_, referenced to protio solvent impurities): δ 179.2, 173.8, 166.8, 166.4, 166.3, 165.9, 163.8, 159.3, 159.0, 158.4, 158.1, 154.5, 153.9, 127.6, 124.1, 123.9, 122.5, 121.8, 121.6, 37.6, 37.6, 37.5, 37.3, 36.7, 36.7, 36.6, 36.6, 31.0, 30.9, 30.6, 30.6, 30.54, 29.0, 25.7, 24.9, 23.5, 5.8. Anal. calcd for [**1A**](OTf)_4_ + 2H_2_O + CH_3_CN: C_64_H_89_S_4_F_12_N_9_O_17_Ir_2_: C, 38.49; H, 4.49; N, 6.31%. Found: C, 38.13; H, 4.41; N, 6.29%.

### [Ir^I^_2_(Butpy)_2_(L_A_)](OTf)_2_ {[2A](OTf)_2_}

[**1A**](OTf)_4_ (12.0 mg, 6.25 μmol) was dissolved to the aqueous solution of sodium hydroxide (1 mM, 8 mL), which was stirred under a H_2_ atmosphere (0.6 MPa) at room temperature for 2 h. Black precipitate was collected by filtration and washed with water in the glovebox. The obtained black solid was dried in vacuo {yield: 68% based on [**1A**](OTf)_4_}. ESI-MS (in methanol): *m*/*z* = 643.3 {[**2A**]^2+^, *I* = 100% in the range of *m*/*z* 100 to 2000}. Anal. calcd for [**2A**](OTf)_2_ + 3H_2_O: C_60_H_84_S_2_F_6_N_8_O_10_Ir_2_: C, 43.94; H, 5.16; N, 6.83%. Found: C, 43.87; H, 4.98; N, 6.53%.

### [Ir^II^_2_(Butpy)_2_(L_B_)(I)_2_](PF_6_) {[1B](PF_6_), L_B_ = CH_3_(C=O)N–C(CH_3_)=NH}

Sodium iodide (61.4 mg, 0.410 mmol), *trans*-stilbene (14.8 mg, 82.1 μmol), and diiodomethane (19.8 μL, 0.246 mmol) were added to the 1-propanol solution (3 mL) of [**2A**](OTf)_2_ (13.0 mg, 8.2 μmol) in the glovebox, which was stirred at 80 °C for 8 h. After the solvent was removed under reduced pressure, CH_2_Cl_2_ (9 mL) was added to the residue, and the insoluble materials were removed by filtration. The filtrate was evaporated under reduced pressure, and methanol (3 mL) and KPF_6_ (15.1 mg, 82.0 μmol) were added to the residue. The resulting solution was stirred for 1 min at room temperature, and the solvent was removed under reduced pressure. CH_2_Cl_2_ (9 mL) was added into the residue, and the insoluble materials were removed by filtration. The solvent was removed under reduced pressure to afford a brown solid, which was recrystallized in methanol (1.5 mL) diffused by diethyl ether for 1 day. The formed crystals were removed by filtration, and the filtrate was evaporated under reduced pressure. The residue was recrystallized in a methanol/acetone (1/1) solution (1 mL) diffused by diethyl ether for 2 days. The red-black crystal was collected by filtration and dried in vacuo {yield: 38% based on [**2A**](OTf)_2_}. ESI-MS (in methanol): *m*/*z* = 1539.3 {[**1B**]^+^, *I* = 100% in the range of *m*/*z* 100–2000}. ^1^H NMR (600 MHz, in CD_3_OD, referenced to TMS): δ 9.48 (s, 1H, −N*H*), 8.37 (d, 2H, −C*H*), 8.30 (d, 2H, −C*H*), 7.90–8.04 (m, 8H, −C*H*), 7.46 (d, 2H, −C*H*), 7.39 (d, 2H, −C*H*), 2.93 (s, 3H, −C–C*H*_3_), 2.70 (s, 3H, −C–C*H*_3_), 1.49–1.57 {s, 54H, −C(C*H*_3_)_3_}. Anal. calcd for [**1B**](PF_6_) + 3H_2_O + CH_3_OH: C_59_H_87_F_6_I_2_N_8_O_5_PIr_2_: C, 40.00; H, 4.95; N, 6.33%. Found: C, 40.23; H, 4.73; N, 6.02%.

### General Procedure for Catalytic Cyclopropanation of Styrene Derivatives, Indene, or Cyclooctene with Diiodomethane (Entries 1–6 and 9–11 in Table 1)

A CD_3_CN solution (500 μL) of [**1A**](OTf)_4_ (2.20 mg, 1.15 μmol), potassium carbonate (31.7 mg, 0.23 mmol), diiodomethane (33.6 μL, 0.42 mmol), and alkene (styrene derivatives, indene, or cyclooctene) (11.5 μmol) was stirred at 80 °C for 10 h under a H_2_ atmosphere (0.5 MPa). After the resulting solution was cooled to room temperature, the products were analyzed and quantified by ^1^H NMR with dibromomethane or 1,4-dioxane as an internal standard. The yields were 57% (entry 1), 55% (entry 2), 44% (entry 3), 38% (entry 4), 33% (entry 5), 16% (entry 6), 20% (entry 9), 14% (entry 10), and 4% (entry 11) as shown in Table 1. The yields were determined as 58% for 4-methylstyrene, 54% for α-methylstyrene, 44% for styrene, and 13% for 4-nitrostyrene with 1,4-dioxane as an internal standard (Table S4). No cyclopropanation products were formed without **1A**, H_2_, or CH_2_I_2_ (Table S2 entries 2, 3, or 4). The conversion of 4-methylstyrene, α-methylstyrene, and styrene is 60, 60, and 53%, respectively. These values were determined by ^1^H NMR spectroscopy. The GC-MS yield (54%) of *p*-tolylcyclopropane, entry 1 in Table 1, was in close agreement with the NMR yield. The authentic sample, *p*-tolylcyclopropane, was synthesized according to the literature.^9^ Other CHRI_2_ such as CMe_2_I_2_ does not act as a carbene source in this cyclopropanation. **1A** does not activate CH_2_I_2_ and H_2_ does not reduce **3A** (Figures S17 and S18).

### General Procedure for Catalytic Cyclopropanation of *Trans*-Stilbene and *Cis*-Stilbene with Diiodomethane (Entries 7 and 8 in Table 1)

A CD_3_CN solution (500 μL) of [**1A**](OTf)_4_ (2.20 mg, 1.15 μmol), potassium carbonate (31.7 mg, 0.23 mmol), diiodomethane (33.6 μL, 0.42 mmol), and *trans*-stilbene or *cis*-stilbene (11.5 μmol) was stirred at 80 °C for 10 h under a H_2_ atmosphere (0.5 MPa). After the resulting solution was cooled to room temperature, the solvent was removed under reduced pressure. The product was extracted with toluene (5 × 1 mL), which was passed through a silica gel column eluted with toluene. After removal of the solvent from the resulting solution, the product was identified by ^1^H NMR in CDCl_3_. The yields were 20% (entry 7) and 12% (entry 8) in Table 1. The isolated yield of *trans*-1,2-diphenylcyclopropane was determined by increasing the scale of the catalytic cyclopropanation of *trans*- or *cis*-stilbene by a factor of 5. After purification of phenylcyclopropane by column chromatography, an isolated yield of 19% or 10% was measured by a balance.

## Supporting Information Available

Experimental details, computational details, IR spectra, ESR spectra, and ESI-mass spectra (PDF)

## References

[ref1] Wessjohann L. A.; Brandt W.; Thiemann T. Biosynthesis and Metabolism of Cyclopropane Rings in Natural Compounds. Chem. Rev. 2003, 103, 1625–1647. 10.1021/cr0100188. 12683792

[ref2] Chen D. Y.-K.; Pouwer R. H.; Richard J.-A. Recent Advances in the Total Synthesis of Cyclopropane-Containing Natural Products. Chem. Soc. Rev. 2012, 41, 4631–4642. 10.1039/c2cs35067j. 22592592

[ref3] Ebner C.; Carreira E. M. Cyclopropanation Strategies in Recent Total Syntheses. Chem. Rev. 2017, 117, 11651–11679. 10.1021/acs.chemrev.6b00798. 28467054

[ref4] Doering W. E.; Hoffmann A. K. The Addition of Dichlorocarbene to Olefins. J. Am. Chem. Soc. 1954, 76, 6162–6165. 10.1021/ja01652a087.

[ref5] Simmons H. E.; Smith R. D. A New Synthesis of Cyclopropanes from Olefines. J. Am. Chem. Soc. 1958, 80, 5323–5324. 10.1021/ja01552a080.

[ref6] Furukawa J.; Kawabata N.; Nishimura J. Syntheshis of Cyclopropanes by the Reaction of Olefins with Dialkylzinc and Methylene Iodide. Tetrahedron 1968, 24, 53–58. 10.1016/0040-4020(68)89007-6.

[ref7] Kanai H.; Hiraki N. Cyclopropanation of Electron-Deficient Olefines with gem-Dibromides Catalyzed by Nickel Catalysts. Chem. Lett. 1979, 8, 761–762. 10.1246/cl.1979.761.

[ref8] Takai K.; Toshikawa S.; Inoue A.; Kokumai R. Stereoselective Iodocyclopropanation of Terminal Alkenes with Iodoform, Chromium(II) Chloride, and N,N,N’,N’-Tetraethylethylenediamine. J. Am. Chem. Soc. 2003, 125, 12990–12991. 10.1021/ja0373061. 14570448

[ref9] Lorenz J. C.; Long J.; Yang Z.; Xue S.; Xie Y.; Shi Y. A Novel Class of Tunable Zinc Reagents (RXZnCH_2_Y) for Efficient Cyclopropanation of Olefins. J. Org. Chem. 2004, 69, 327–334. 10.1021/jo030312v. 14725443

[ref10] Zhou Y.-Y.; Uyeda C. Reductive Cyclopropanations Catalyzed by Dinuclear Nickel Complexes. Angew. Chem., Int. Ed. 2016, 55, 3171–3175. 10.1002/anie.201511271. 26822193

[ref11] del Hoyo A. M.; Herraiz A. G.; Suero M. G. A Stereoconvergent Cyclopropanation Reaction of Styrenes. Angew. Chem., Int. Ed. 2017, 56, 1610–1613. 10.1002/anie.201610924. 27981721

[ref12] Werth J.; Uyeda C. Regioselective Simmons–Smith-Type Cyclopropanations of Polyalkenes Enabled by Transition Metal Catalysis. Chem. Sci. 2018, 9, 1604–1609. 10.1039/C7SC04861K. 29675205 PMC5890799

[ref13] Werth J.; Uyeda C. Cobalt-Catalyzed Reductive Dimethylcyclopropanation of 1,3-Dienes. Angew. Chem., Int. Ed. 2018, 57, 13902–13906. 10.1002/anie.201807542. PMC6489504 30144253

[ref14] Peil S.; Guthertz A.; Biberger T.; Fürstner A. Hydrogenative Cyclopropanation and Hydrogenative Metathesis. Angew. Chem., Int. Ed. 2019, 58, 8851–8856. 10.1002/anie.201904256. 31025795

[ref15] Ikeda H.; Nishi K.; Tsurugi H.; Mashima K. Chromium-Catalyzed Cyclopropanation of Alkenes with Bromoform in the presence of 2,3,5,6-Tetramethyl-1,4-bis(trimethylsilyl)-1,4-dihydropyrazine. Chem. Sci. 2020, 11, 3604–3609. 10.1039/D0SC00964D. 34094048 PMC8152687

[ref16] Wei N.; Yang D.; Zhao J.; Mei T.; Zhang Y.; Wang B.; Qu J. Structure and Methylene Transfer Reactivity of Thiolate-Bridged Dichromium Methylene Complexes Derived from Dihalomethane via Cleavage of Two Carbon–Halogen Bonds. Organometallics 2021, 40, 1434–1442. 10.1021/acs.organomet.1c00031.

[ref17] Ramachandran R.; Menon R. K. An Overview of Industrial Uses of Hydrogen. Int. J. Hydrogen Energy 1998, 23, 593–598. 10.1016/S0360-3199(97)00112-2.

[ref18] Wilkinson G. Die lange Suche nach stabilen Alkyl-Übergangsmetall-Verbindungen. Angew. Chem. 1974, 86, 664–667. 10.1002/ange.19740861803.

[ref19] Knowles W. S. Asymmetric Hydrogenations. Angew. Chem., Int. Ed. 2002, 41, 1998–2007. 10.1002/1521-3773(20020617)41:12<1998::AID-ANIE1998>3.0.CO;2-8. 19746594

[ref20] Noyori R. Asymmetric Catalysis: Science and Opportunities. Angew. Chem., Int. Ed. 2002, 41, 2008–2022. 10.1002/1521-3773(20020617)41:12<2008::AID-ANIE2008>3.0.CO;2-4. 19746595

[ref21] Ngai M.-Y.; Kong J.-R.; Krische M. J. Hydrogen-Mediated C-C Bond Formation: A Broad New Concept in Catalytic C-C Coupling. J. Org. Chem. 2007, 72, 1063–1072. 10.1021/jo061895m. 17288361

[ref22] Hong Y.-T.; Barchuk A.; Krische M. J. Branch-Selective Intermolecular Hydroacylation: Hydrogen-Mediated Coupling of Anhydrides to Styrenes and Activated Olefins. Angew. Chem., Int. Ed. 2006, 45, 6885–6888. 10.1002/anie.200602377. 16991162

[ref23] Nakamura M.; Hirai A.; Nakamura E. Reaction Pathways of the Simmons–Smith Reaction. J. Am. Chem. Soc. 2003, 125, 2341–2350. 10.1021/ja026709i. 12590564

[ref24] Leung C. W.; Zheng W.; Zhou Z.; Lin Z.; Lau C. P. Mechanism of Catalytic Hydration of Nitriles with Hydrotris(pyrazolyl)borato (Tp) Ruthenium Complexes. Organometallics 2008, 27, 4957–4969. 10.1021/om800474w.

[ref25] Khusnutdinova J. R.; Luo J.; Rath N. P.; Mirica L. M. Late First-Row Transition Metal Complexes of a Tetradentate Pyridinophane Ligand: Electronic Properties and Reactivity Implications. Inorg. Chem. 2013, 52, 3920–3932. 10.1021/ic400260z. 23517006

[ref26] Kukushkin V. Y.; Pombeiro A. J. L. Metal-mediated and Metal-Catalyzed Hydrolysis of Nitriles. Inorg. Chim. Acta 2005, 358, 1–21. 10.1016/j.ica.2004.04.029.

[ref27] Ogo S. H_2_ and O_2_ Activation—A Remarkable Insight into Hydrogenase. Chem. Rec. 2014, 14, 397–409. 10.1002/tcr.201402010. 24890792

[ref28] Ogo S. H_2_ and O_2_ Activation by [NiFe]hydrogenases – Insights from Model Complexes. Coord. Chem. Rev. 2017, 334, 43–53. 10.1016/j.ccr.2016.07.001.

[ref29] Garg K.; Matsubara Y.; Ertem M. Z.; Lewandowska-Andralojc A.; Sato S.; Szalda D. J.; Muckerman J. T.; Fujita E. Striking Differences in Properties of Geometric Isomers of [Ir(tpy)(ppy)H]^+^: Experimental and Computational Studies of their Hydricities, Interaction with CO_2_, and Photochemistry. Angew. Chem., Int. Ed. 2015, 54, 14128–14132. 10.1002/anie.201506961. 26427767

[ref30] Smirnov M. Y.; Kalinkin A. V.; Kovtunova L. M.; Bukhtiyarov V. I. Deposition of [Ir(COD)(IMes)Cl] Complex on the HOPG Surface by Means of Evaporation in Vacuum. Surf. Interfaces 2021, 25, 101176 10.1016/j.surfin.2021.101176.

[ref31] Jiménez M. V.; Sola E.; Francisco A. C.; Oro L. A.; Lahoz F. J.; Martínez A. P. Methylene- and Diamidonaphthalene-Bridged Diiridium(III) Complexes. Inorg. Chim. Acta 2003, 350, 266–276. 10.1016/S0020-1693(02)01549-9.

[ref32] Berry J. F. Metal-Metal Bonded Compounds of the Group IX Elements. Comprehensive Coordination Chemistry III; Elsevier: 2021; pp. 4–42.

[ref33] Allen F. H.; Kennard O.; Watson D. G.; Brammer L.; Orpen A. G.; Taylor R. Tables of Bond Lengths Determined by X-ray and Neutron Diffraction. Part 1. Bond Lengths in Organic Compounds. J. Chem. Soc., Perkin Trans. 1987, II, S1–S19. 10.1039/p298700000s1.

[ref34] van Beek C. B.; van Leest N. P.; Lutz M.; de Vos S. D.; Gebbink R. J. M. K.; de Bruin B.; Broere D. L. J. Combining metal–metal cooperativity, metal– ligand cooperativity and chemical non-innocence in diiron carbonyl complexes. Chem. Sci. 2022, 13, 2094–2104. 10.1039/d1sc05473b. 35308864 PMC8849050

[ref35] Ciriano M. A.; Viguri F.; Oro L. A.; Tiripicchio A.; Tiripicchio-Camellini M. Diiridium(II)-Komplexe durch Iichtunterstützte Oxidative Additionsreaktionen: Struktur eines stabilen zweikernigen Iodo(iodmethyl)iridium(II)-Komplexes. Angew. Chem. 1987, 99, 452–453. 10.1002/ange.19870990508.

[ref36] Isegawa M.; Sharma A. K.; Ogo S.; Morokuma K. Electron and Hydride Transfer in a Redox-Active NiFe Hydride Complex: A DFT Study. ACS Catal. 2018, 8, 10419–10429. 10.1021/acscatal.8b02368.

[ref37] Isegawa M.; Matsumoto T.; Ogo S. H_2_ activation by hydrogenase-inspired NiFe catalyst using frustrated Lewis pair: effect of buffer and halide ion in the heterolytic H–H bond cleavage. RSC Adv. 2021, 11, 28420–28432. 10.1039/D1RA05928A. 35480737 PMC9038005

[ref38] Porras J. A.; Mills I. N.; Transue W. J.; Bernhard S. Highly Fluorinated Ir(III) – 2,2’:6’,2”-Terpyridine–Phenylpyridine–X Complexes via Selective C–F Activation: Robust Photocatalysts for Solar Fuel Generation and Photoredox Catalysis. J. Am. Chem. Soc. 2016, 138, 9460–9472. 10.1021/jacs.6b03246. 27387149

[ref39] Moulder J. F.; Stickle W. F.; Sobol P. E.; Bomben K. D. Handbook of X-ray Photoelectron Spectroscopy, Chastain J.; King R. C., Eds.; Physical Electronics Eden Prairie, 1995; pp. 40–41.

