# Supplementary material for: Cyclopropanation Using Electrons Derived from Hydrogen: Reaction of Alkenes and Hydrogen without Hydrogenation
Source: JACS Au. 2024 Mar 25;4(4):1615–22. doi: 10.1021/jacsau.4c00098 (PMC11040560; doi:10.1021/jacsau.4c00098)
Supplement: Supplementary file 1 — au4c00098_si_001.pdf [file au4c00098_si_001.pdf]

Supporting Information for

Cyclopropanation using Electrons Derived from Hydrogen—Reaction of Alkenes and Hydrogen without Hydrogenation

Seiji Ogo,*[a],[b],[c] Takeshi Yatabe,[a],[b],[c] Keishi Miyazawa,[a],[b] Yunosuke Hashimoto,[a],[b] Chiaki Takahashi,[a],[b] Hidetaka Nakai,[d] Yoshihito Shiota[e]

[a]Department of Chemistry and Biochemistry, Graduate School of Engineering, Kyushu University, 744 Moto-oka, Nishi-ku, Fukuoka 819-0395, Japan

[b]International Institute for Carbon-Neutral Energy Research (WPI Academy I2CNER), Kyushu University, 744 Moto-oka, Nishi-ku, Fukuoka 819-0395, Japan

[c]Center for Small Molecule Energy, Kyushu University, 744 Moto-oka, Nishi-ku, Fukuoka 819-0395, Japan

[d]Department of Energy and Materials, Faculty of Science and Engineering, Kindai University, 3-4-1 Kowakae, Higashi-Osaka, Osaka 577-8502, Japan

[e]Institute for Materials Chemistry and Engineering, Kyushu University, 744 Moto-oka, Nishi-ku, Fukuoka 819-0395, Japan

*To whom correspondence should be addressed.

E-mail: ogo.seiji.872@m.kyushu-u.ac.jp

This PDF file includes:

Experimental section

Figures S1 to S18

Tables S1 to S17

References

A Table of Contents

Experimental section	page 1
Contents of figures	page 3
Figure S1	page 4
Figure S2	page 5
Figure S3	page 6
Figure S4	page 7
Figure S5	page 8
Figure S6	page 9
Figure S7	page 10
Figure S8	page 11
Figure S9	page 12
Figure S10	page 13
Figure S11	page 14
Figure S12	page 15
Figure S13	page 16
Figure S14	page 17
Figure S15	page 18
Figure S16	page 19
Figure S17	page 20
Figure S18	page 21
Table S1	page 22
Table S2	page 23
Table S3	page 24
Table S4	page 25
Table S5	page 26
Table S6	page 31
Table S7	page 37
Table S8	page 43
Table S9	page 49

Table S10	page 55
Table S11	page 61
Table S12	page 67
Table S13	page 73
Table S14	page 79
Table S15	page 85
Table S16	page 91
Table S17	page 97
References	page 102

Experimental Section

[Ir^{II}_2(Butpy)$_2$(CD_3(OH)C=N–C(CD_3)=NH)(OH_2)$_2$](OTf)$_4$ {[D-labeled 1A](OTf)$_4$} [D-labeled **1A**](OTf)$_4$ was synthesized by the same procedure as [**1A**](OTf)$_4$ except CD_3CN was used. ^{1}H NMR (600 MHz, in DMSO-*d*$_6$, referenced to TMS): δ 10.9 and 9.63 (s, 2H, –N*H* and –O*H*), 9.05 (s, 1H, –C*H*), 8.66 (s, 1H, –C*H*), 8.49 (d, 1H, –C*H*), 8.41 (d, 1H, –C*H*), 8.33-8.36 (m, 3H, –C*H*), 8.25 (s, 1H, –C*H*), 8.22 (s, 2H, –C*H*), 8.04 (d, 1H, –C*H*), 7.79-7.82 (m, 2H, –C*H*), 7.31 (d, 1H, –C*H*), 7.17 (d, 1H, –C*H*), 7.04 (d, 1H, –C*H*), 1.54-1.43 {s, 54H, –C(CH_3)$_3$}.

Detection of carbenoid complex [Ir^{II}_2(Butpy)$_2$(CH_2I)(L_A)(I)](OTf)$_2$ {[3A](OTf)$_2$}. Diiodomethane (10.2 μL, 0.127 mmol) was added to an acetonitrile solution (1 mL) of [**2A**](OTf)$_2$ (6.7 mg, 4.2 μmol) under an N_2 atmosphere, which was stirring for 1 min at room temperature. ^{1}H NMR (600 MHz, in acetone-*d*$_6$, referenced to protio solvent impurities): δ 5.31 (s, 2H, Ir–CH_2–I). ESI-MS (in CH_3CN and methanol): *m*/*z* = 713.2 {[**3A**–I–H]$^{2+}$, *I* = 100% in the range of *m*/*z* 100 to 2000}.

Detection of ^{13}C-labeled carbenoid complex [Ir^{II}_2(Butpy)$_2$($^{13}CH_2I$)(L_A)(I)](OTf)$_2$ {[^{13}C-labeled 3A](OTf)$_2$}. ^{13}C-labeled diiodomethane (0.8 μL, 9.9 μmol) was added to a CD_3CN solution (500 μL) of [**2A**](OTf)$_2$ (3.0 mg, 1.9 μmol) under an N_2 atmosphere, which was stirring for 1 min at room temperature. The formed ^{13}C-labeled **3A** was detected by NMR and ESI-MS. ^{1}H NMR (600 MHz, in acetone-*d*$_6$, referenced to protio solvent impurities): δ 5.31 (d, 2H, Ir–CH_2–I, $^1J_{CH}$ = 153 Hz). ^{13}C NMR (150 MHz, in CD_3CN, referenced to TMS): δ –23.0 (s, Ir*C*H_2I). ESI-MS (in CH_3CN and methanol): *m*/*z* = 713.8 {[^{13}C-labeled **3A**–I–H]$^{2+}$, *I* = 100% in the range of *m*/*z* 100 to 2000}.

Formation of 1-phenylcyclopropane from the reaction of 3A with styrene and diiodomethane. Potassium carbonate (35 mg, 0.25 mmol), styrene (1.4 μL, 12 μmol), and diiodomethane (37 μL, 0.46 mmol) were added to a CD_3CN solution (550 μL) of [**2A**](OTf)$_2$ (2.0 mg, 1.3 μmol) in the glovebox, which was stirring at 80 ºC for 10 h. The formed 1-phenylcyclopropane in the resulting solution was quantified by ^{1}H NMR with dibromomethane as an internal standard {yield: 47% based on [**2A**](OTf)$_2$}.

Detection of 2A from the reaction of 1B with H_2. [**1B**](PF_6) (1.0 mg, 0.59 μmol) and potassium acetate (2.9 mg, 30 μmol) was dissolved to 1-propanol (1 mL), which was stirring under an H_2 atmosphere (0.5 MPa) at room temperature for 5 min. The resulting solution was

diluted with methanol and analyzed by ESI-MS. ESI-MS (in methanol): m/z = 1413.6 {[**2A**+I]$^+$, I = 100% in the range of m/z 100 to 2000}.

Catalytic cyclopropanation of styrene with diiodomethane in the presence of PBN. The mixture solution of [**1A**]$(OTf)_4$ (2.20 mg, 1.15 μmol), potassium carbonate (31.7 mg, 0.23 mmol), diiodomethane (33.6 μL, 0.42 mmol), PBN (40.6 mg, 0.23 mmol), and styrene (1.33 μL, 11.6 μmol) in CD_3CN solution (500 μL) was stirring at 80 ºC for 10 h under an H_2 atmosphere (0.5 MPa). After the resulting solution was cooled to room temperature, the products were analyzed and quantified by ^{1}H NMR with dibromomethane as an internal standard.

Catalytic cyclopropanation of styrene with ^{13}C-labeled diiodomethane. A CD_3CN solution (500 μL) of [**1A**]$(OTf)_4$ (2.20 mg, 1.15 μmol), potassium carbonate (31.7 mg, 0.23 mmol), ^{13}C-diiodomethane (33.6 μL, 0.42 mmol), and styrene (1.33 μL, 11.6 μmol) was stirring at 80 ºC for 10 h under an H_2 atmosphere (0.5 MPa). After cooling to room temperature, the resulting solution was passed through the short silica gel column to remove Ir complex. The ^{13}C-labeled phenylcyclopropane was analyzed by GC-MS.

Computational Details. The density functional theory (DFT) calculations were performed with the Gaussian 16 program package (Rev. C01).[1] All geometry optimizations were carried out with the B3LYP functional.[2-4] We employed the SDD (Stuttgart/Dresden pseudopotentials) basis set[5] for Ir and the D95** basis set[6] for the other atoms. After geometry optimizations, vibrational analyses were calculated for all reaction species to confirm stable and transition structures. Energy profiles of calculated species are presented as the Gibbs free energy changes at 298.15 K.

X-ray Crystallographic Analysis of 1B. A red black crystal of [**1B**](PF_6) was obtained from a methanol/acetone (1/1) solution diffused with diethyl ether at room temperature. Measurements were made on a Rigaku XtalLAB/HyPix-6000HE with graphite monochromated Mo-Kα radiation (λ = 0.71070 Å). Data were collected and processed using the CrysAlis Pro program. All calculations were performed using the Olex2 except for refinement, which was performed using SHELXL-97. Crystallographic data for **1B** have been deposited with the Cambridge Crystallographic Data Centre as Supplementary Publication No. 2246755 (complex **1B**). Copies of the data can be obtained free of charge on application to CCDC, 12 Union Road, Cambridge CB2 1EZ, UK {fax: (+44)1223-336-033; e-mail: deposit@ccdc.cam.ac.uk}.

Contents of figures

	Ir^{II} dinuclear complex **1A**	Ir^{I} low-valent complex **2A**	Ir^{II} carbenoid complex **3A**	Ir^{II} dinuclear complex **1B**
X-ray	–	–	–	Fig. 6
ESI-MS	Fig. S2	Fig. 4	Fig. 5	Fig. S11
^{1}H NMR	Fig. 2	–	Fig. S7	Fig. 7
^{13}C NMR	Fig. S1	–	Fig. S8	–
UV-vis-NIR	Fig. S4	Fig. S4	Fig. S10	–
XPS	Fig. 3	Fig. 3	–	–
FT-IR	Fig. S3	Fig. S5	–	–
ESR	–	Fig. S6	Fig. S9	

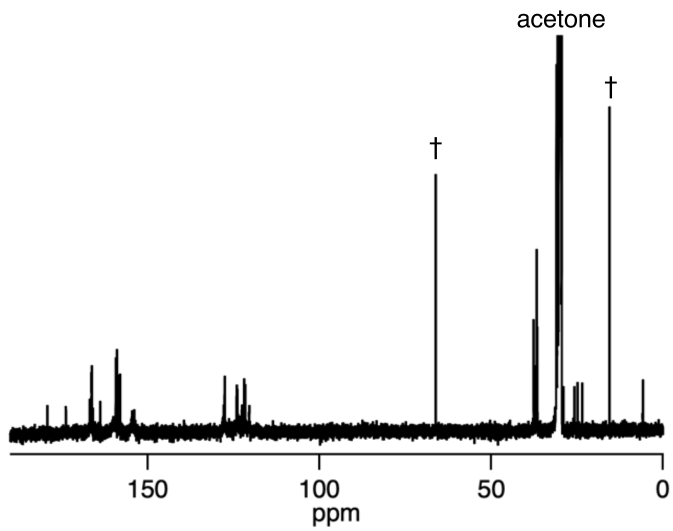

Fig. S1. ^{13}C NMR spectrum of **1A** in acetone-d_6. †: Peaks of diethyl ether.

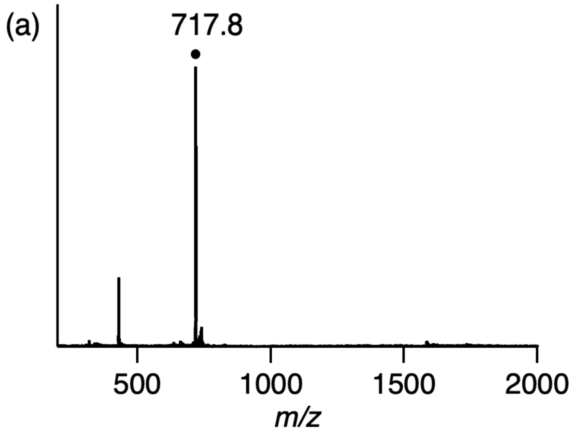

Fig. S2. (a) Positive-ion ESI-mass spectrum of **1A** in methanol. The signal at *m*/*z* 717.8 corresponds to [**1A**+OTf–2H_2O–2H]$^{2+}$. (b) The signal at *m*/*z* 717.8 for [**1A**+OTf–2H_2O–2H]$^{2+}$. (c) The calculated isotopic distribution for [**1A**+OTf–2H_2O–2H]$^{2+}$.

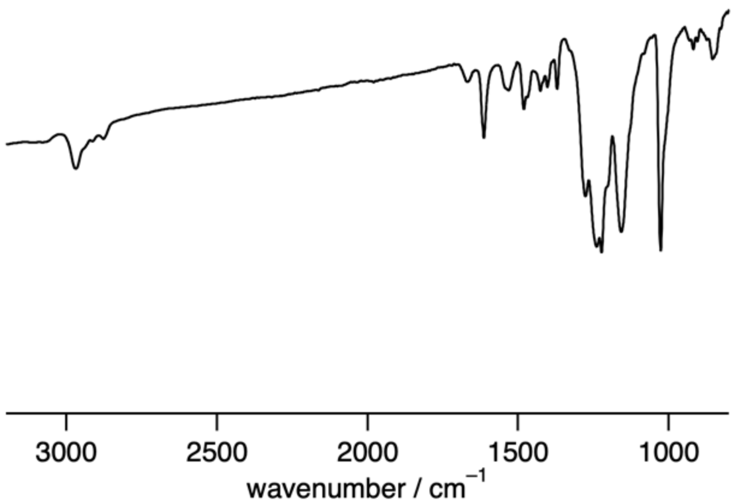

Fig. S3. IR spectrum of **1A**.

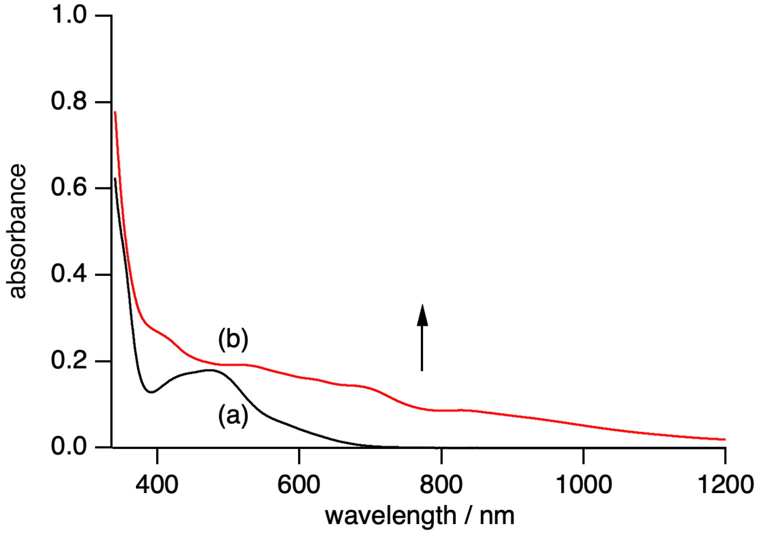

Fig. S4. UV-vis-NIR absorption spectra of (a) **1A** (0.37 mM) in acetone and (b) **2A** (0.37 mM) in acetone. The light path length is 0.1 cm.

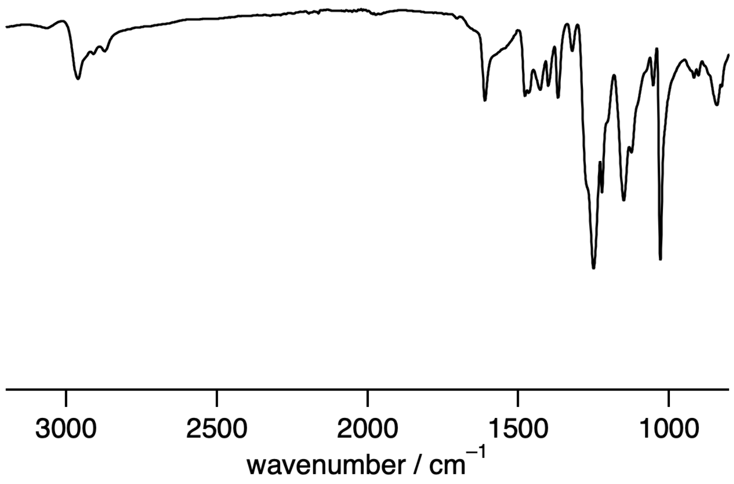

Fig. S5. IR spectrum of **2A**.

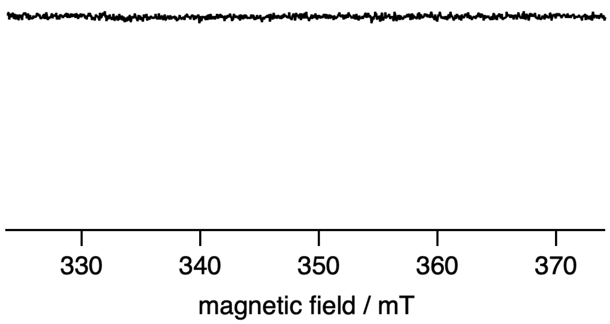

Fig. S6. ESR spectrum of **2A** in acetone at –150 °C.

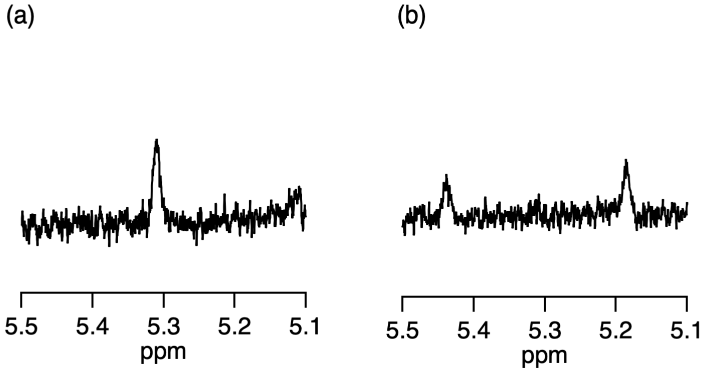

Fig. S7. ^{1}H NMR spectra of **3A** prepared from the reaction of **2A** with 5 equivalents of CH_2I_2 in acetone-d_6 (a) and ^{13}C-labeled **3A** prepared from the reaction of **2A** with 5 equivalents of $^{13}CH_2I_2$ in acetone-d_6 (b).

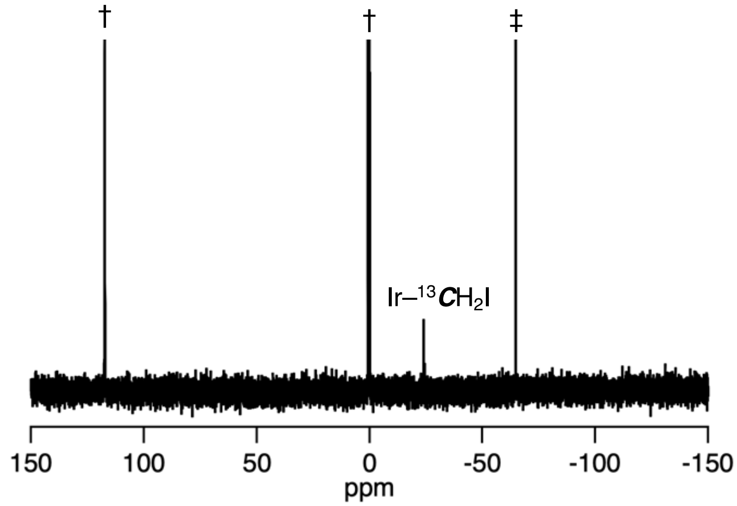

Fig. S8. ^{13}C NMR spectrum of ^{13}C-labeled **3A** prepared from the reaction of **2A** with 5 equivalents of $^{13}CH_2I_2$ in CD_3CN. †: Peaks of CH_3CN. ‡: Peak of ^{13}C-labeled diiodomethane.

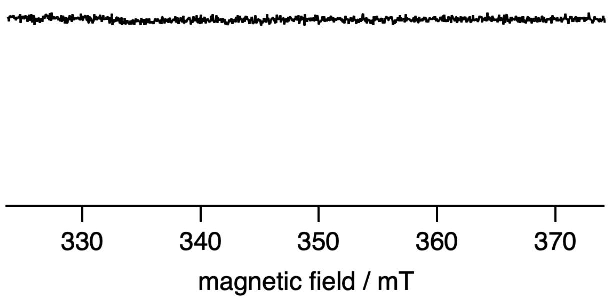

Fig. S9. ESR spectrum of **3A** in acetone at –150 °C.

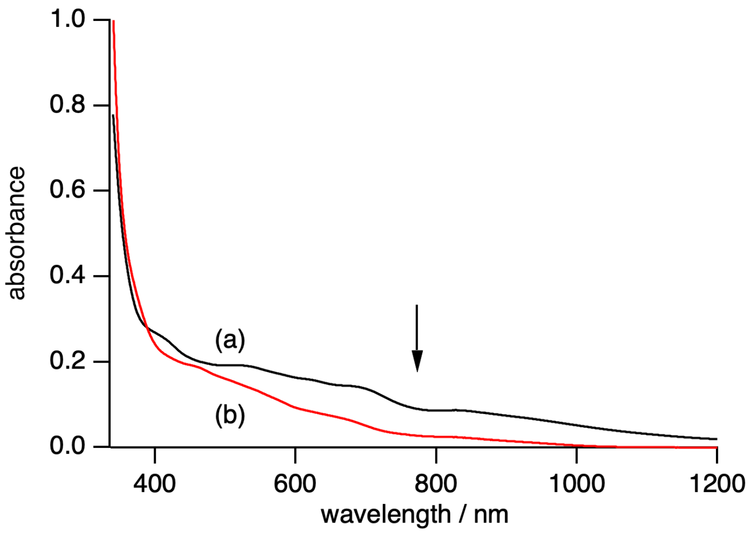

Fig. S10. UV-vis-NIR absorption spectra of **2A** (0.37 mM) in acetone (a) and **3A** prepared from the reaction of **2A** (0.37 mM) with CH_2I_2 in acetone (b). The light path length is 0.1 cm.

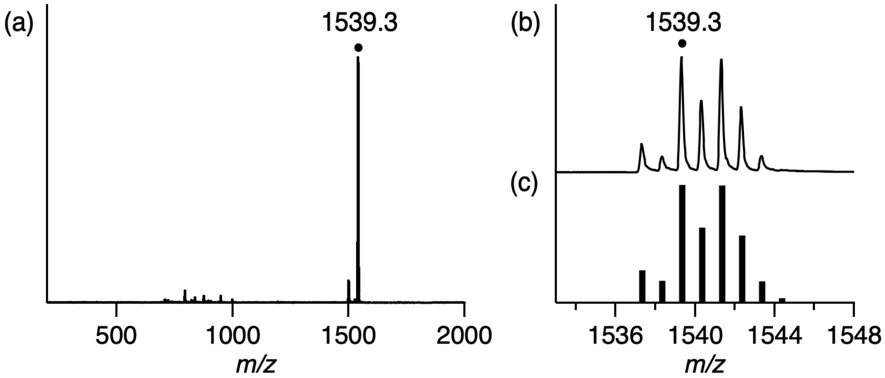

Fig. S11. (a) Positive-ion ESI-mass spectrum of **1B** in methanol. The signal at *m*/*z* 1539.3 corresponds to [**1B**]$^+$. (b) The signal at *m*/*z* 1539.3 for [**1B**]$^+$. (c) The calculated isotopic distribution for [**1B**]$^+$.

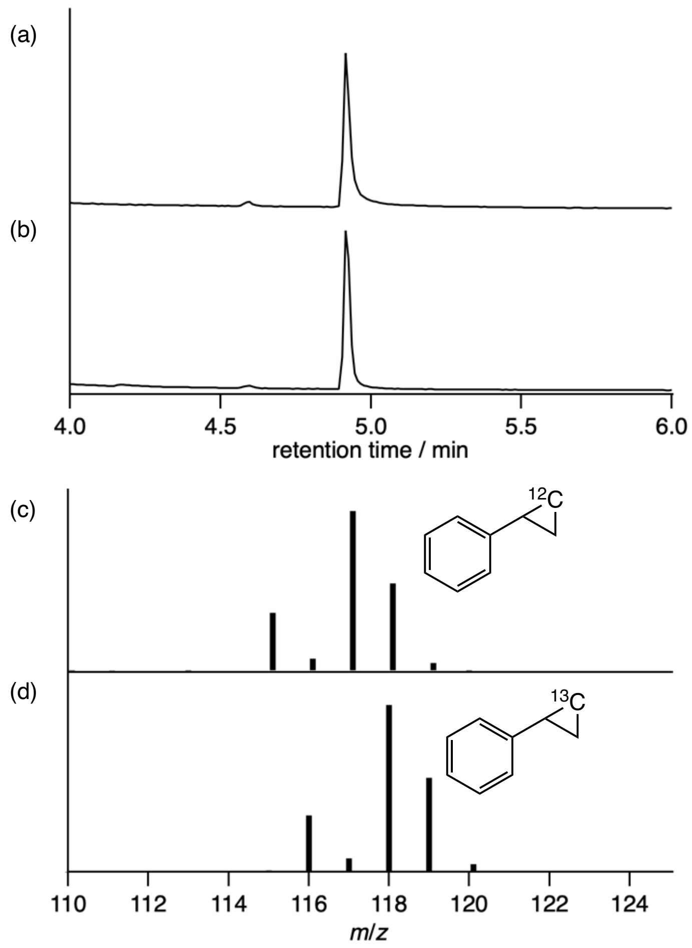

Fig. S12. Gas chromatogram of (a) phenylcyclopropane and (b) ^{13}C-labeled phenylcyclopropane from the catalytic reaction. Mass spectra of a gas chromatogram of (c) phenylcyclopropane and (d) ^{13}C-labeled phenylcyclopropane from the catalytic reaction.

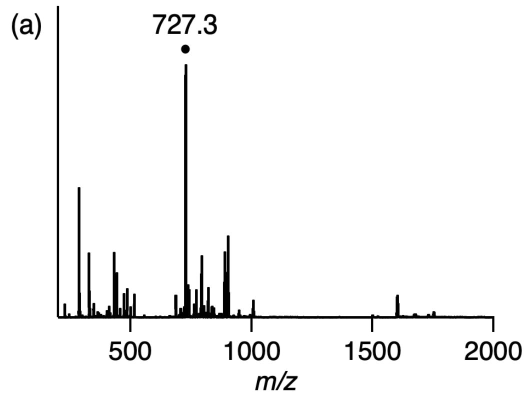

Fig. S13. (a) Positive-ion ESI-mass spectrum of the catalytic reductive cyclopropanation of alkene (11.5 μmol) with **1A** (1.15 μmol), CH_2I_2 (0.42 mmol), K_2CO_3 (0.23 mmol), and H_2 (0.5 MPa) in CD_3CN (500 μL) at 80 °C. The signal at *m*/*z* 727.3 corresponds to $[\mathbf{1B}–I–H]^{2+}$. (b) The signal at *m*/*z* 727.3 for $[\mathbf{1B}–I–H]^{2+}$. (c) The calculated isotopic distribution for $[\mathbf{1B}–I–H]^{2+}$.

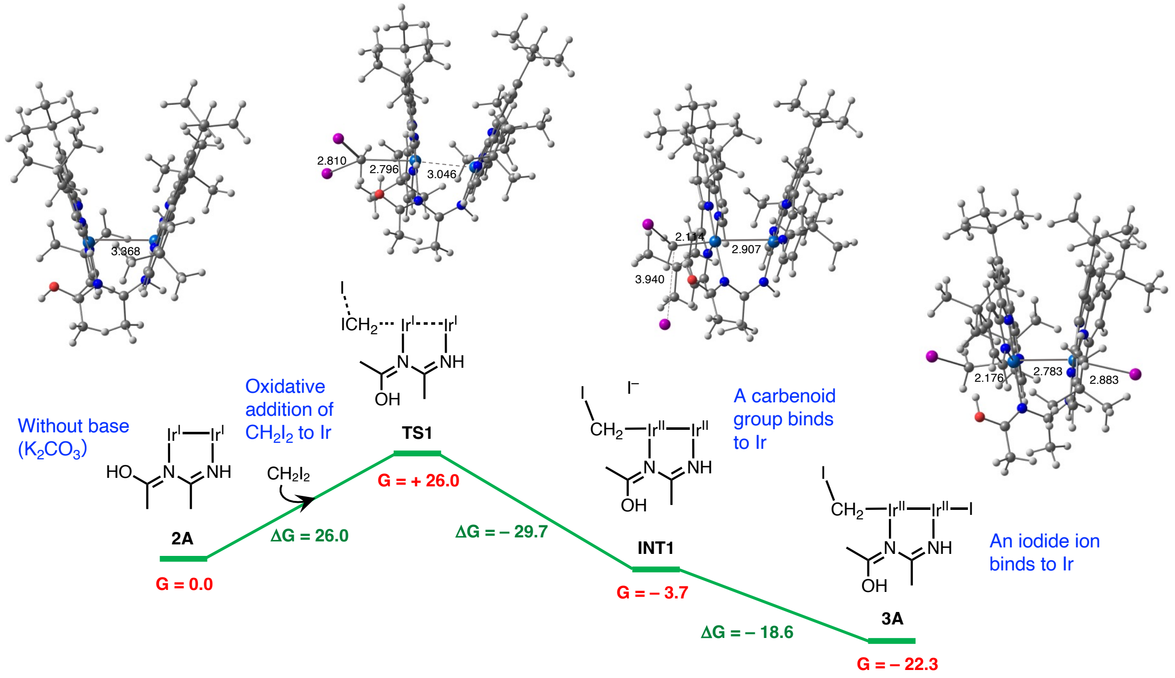

Fig. S14. Optimized geometries and free energy diagrams for the formation of complex **3A** at 298.15 K in the singlet state (S = 0). Units are in kcal/mol and angstrom.

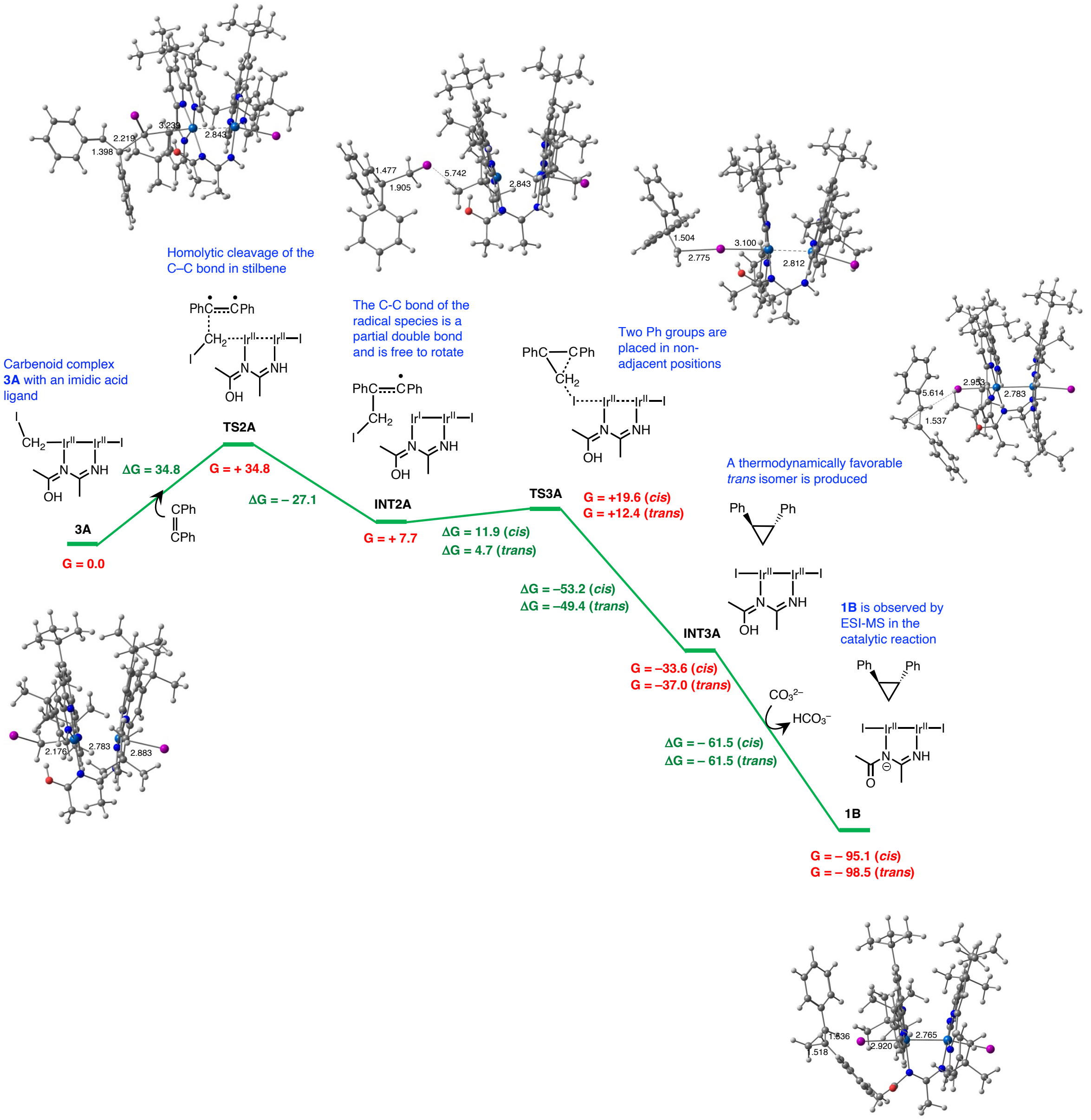

Fig. S15. Optimized geometries and free energy diagrams for the formation of **1B** and cyclopropane from **3A** and *cis*-stilbene at 298.15 K in the singlet state (S = 0). Units are in kcal/mol and angstrom.

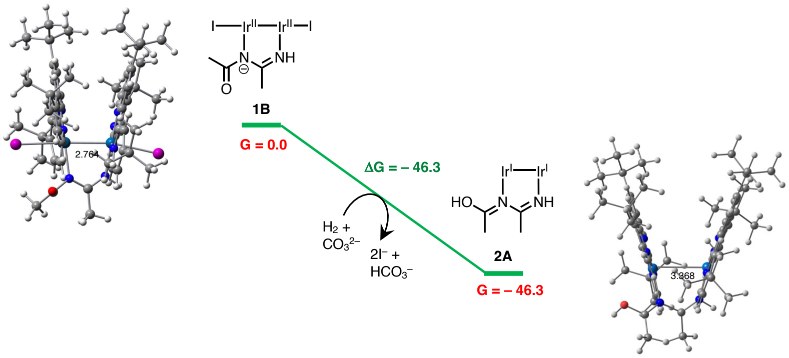

Fig. S16. Optimized geometries and free energy diagrams for the recovery of **2A** by the reaction of **1B** with H_2 at 298.15 K in the singlet state (S = 0). Units are in kcal/mol and angstrom.

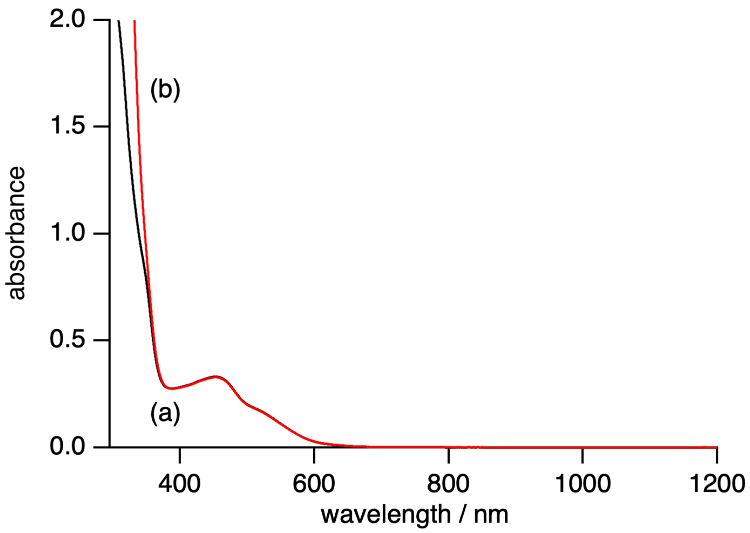

Fig. S17. UV-vis-NIR absorption spectra of (a) **1A** (0.52 mM) in CH_3CN and (b) **1A** (0.52 mM) with CH_2I_2 in CH_3CN. The light path length is 0.1 cm.

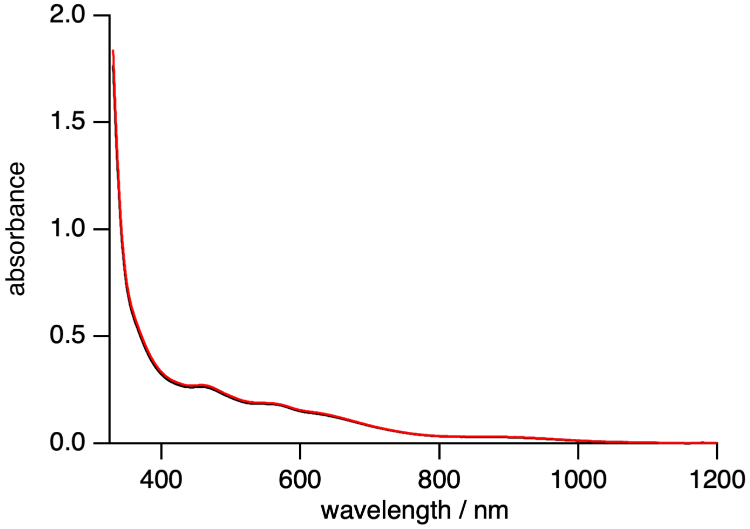

Fig. S18. UV-vis-NIR absorption spectra of (a) **3A** (0.38 mM) in CH_3CN under an N_2 atmosphere and (b) **3A** (0.38 mM) in CH_3CN under an H_2 atmosphere. The light path length is 0.1 cm.

Table S1. Representative reductive cyclopropanation

Entry	Reductant	C source	Substrate	Solvent	Stereoselectivity for *cis*- or *trans*-alkene	Research group	Year	Ref.
1	Na	$CHBr_3$	cyclohexene	*tert*-BuOH, $CHBr_3$	–	Doering, Hoffmann	1954	*7*
2	Zn(Cu)	CH_2I_2	cyclohexene<br>*trans*-3-hexene	diethyl ether	stereospecific[a]	Simmons, Smith	1958	*8*
3	$ZnEt_2$	CH_2I_2	cyclohexene<br>terminal alkenes<br>vinyl ethers	diethyl ether, benzene	stereospecific[a]	Furukawa	1968	*9*
4	Zn	CH_2Br_2	methyl acrylate<br>methyl vinyl ketone<br>acrylonitrile	THF	–	Kanai	1979	*10*
5	$CrCl_2$	CHI_3	terminal alkenes	THF	–[b]	Takai	2003	*11*
6	$ZnEt_2$ ($CF_3CO_2ZnCH_2I$)	CH_2I_2	styrene derivatives<br>stilbene derivatives	CH_2Cl_2	stereospecific[a]	Shi	2004	*12*
7	Zn, $ZnEt_2$	CH_2Cl_2	styrene derivatives<br>dialkyl ethylene<br>cyclic alkenes	CH_2Cl_2	stereospecific[a]	Uyeda	2016	*13*
8	$^{i}Pr_2EtN$	CH_2I_2	styrene derivatives	CH_3CN, H_2O	*trans* selective[c]	Suero	2017	*14*
9	Zn	CH_2I_2	polyalkenes	THF	stereospecific[a]	Uyeda	2018	*15*
10	Zn	CMe_2Cl_2	1,3-dienes	THF	stereospecific[a]	Uyeda	2018	*16*
11	H_2 (0.1 MPa)	alkyne	alkenes (hydrogenative cyclopropanation)[e]	DCE[d]	–	Fürstner	2019	*17*
12	Silicon	$CHBr_3$	allyl ether derivatives<br>terminal alkenes<br>cyclic alkene	DME[f]	–[b]	Mashima	2020	*18*
13	Zn	CH_2Br_2	styrene	DME[f]	–	Yang, Qu	2021	*19*
14	H_2	CH_2I_2	styrene derivatives	CH_3CN	*trans* selective[c]	Ogo	2024	this work

[a]stereospecific: *cis*- and *trans*-product was formed from *cis*- and *trans*-alkene, respectively (Simmons-Smith-type).

[b]*trans*-product was selectively formed from terminal alkene.

[c]*trans* selective: *trans*-product was selectively formed from *cis*- or *trans*-alkene.

[d]1,2-dichloroethane.

[e]intramolecular cyclopropanation with hydrogenation.
[f]1,2-dimethoxyethane.

Table S2. Representative reductive cyclopropanation[a]

Entry	Alkene	Cyclopropanation product	Yield (%)[b] (TON)	Hydrogenation product	Yield (%)[b] (TON)
1	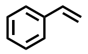	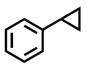	44 (4.4)	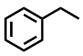	0 (0)
2[c]	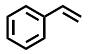 Without **1A**	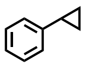	0 (0)	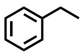	0 (0)
3[d]	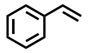 Without H_2	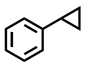	0 (0)	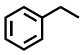	0 (0)
4[e]	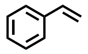 Without CH_2I_2	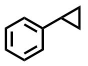	0 (0)	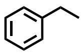	58 (5.8)
5[f]	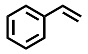 With [Ir^{III}(Butpy)Cl_3] (20 mol%)	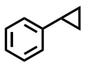	5 (0.2)	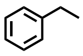	0 (0)
6[g]	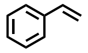 With Cs_2CO_3	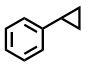	40 (4.0)	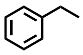	0 (0)
7[h]	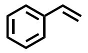 50 equivalents of CH_2I_2	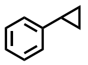	41 (4.1)	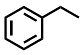	2 (0.2)
8[i]	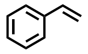 With H_2O	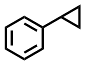	32 (3.2)	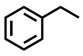	3 (0.3)

[a]Reaction conditions: complex **1A** (1.15 µmol), alkene (11.5 µmol), CH_2I_2 (0.42 mmol), K_2CO_3 (0.23 mmol), CD_3CN (500 µL), H_2 (0.5 MPa), 10 h, 80 °C. The reaction starts with **1A**, which is easier to synthesize than **1B**. [b]The yields of the products of cyclopropanation and hydrogenation were determined by ^{1}H NMR without purification because of the low boiling points of the products. Turnover numbers (TONs, mol of cyclopropane compounds/mol of **1A**) based on complex **1A**. [c]Reaction performed without **1A**. [d]Reaction performed without H_2. [e]Reaction performed without CH_2I_2. [f]Reaction performed with 20 mol% of [Ir^{III}(Butpy)Cl_3] instead of **1A**. [g]Reaction performed with Cs_2CO_3 instead of K_2CO_3. [h]Reaction performed with 50 equivalents of CH_2I_2. [i]Reaction performed with 50 µL of H_2O.

Table S3. Catalytic hydrogenation of alkene using Pd/C under H_2[a]

Entry	Alkene	Cyclopropanation product	Yield (%)[b] (TON)	Hydrogenation Product	Yield (%)[b] (TON)
1	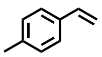	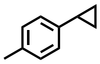	0 (0)	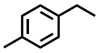	62 (6.2)
2	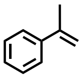	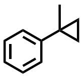	0 (0)	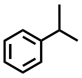	37 (3.7)
3	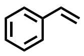	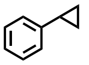	0 (0)	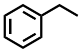	47 (4.7)

[a]Reaction conditions: Pd/C (1.15 µmol), alkene (11.5 µmol), CH_2I_2 (0.42 mmol), K_2CO_3 (0.23 mmol), CD_3CN (500 µL), H_2 (0.5 MPa), 10 h, 80 °C. [b]The yields of the products of cyclopropanation and hydrogenation were determined by ^{1}H NMR without purification because of the low boiling points of the products. Turnover numbers (TONs, mol of cyclopropane compounds/mol of Pd containing of Pd/C) based on Pd/C.

Table S4. Yield of cyclopropanation with 1,4-dioxane or CH_2Br_2 as internal standard (IS)

Entry	Alkene	Cyclopropanation product	Yield obtained with 1,4-dioxane as IS (%)	Yield obtained with CH_2Br_2 as IS (%)
1	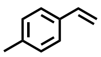	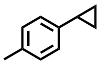	58	57
2	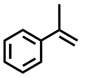	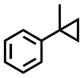	54	55
3	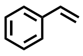	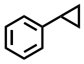	44	44
4	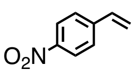	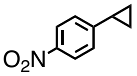	13	16
5	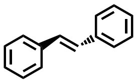 *trans*	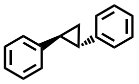 *trans* (100%)	19 (isolated)	20
6	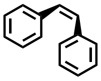 *cis*	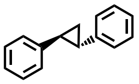 *trans* (100%)	10 (isolated)	12

Table S5. Optimized structure of **2A** at the B3LYP level

Atom	X	Y	Z
Ir	-0.871129	-1.774968	0.641206
Ir	0.854779	-0.292452	-1.842253
O	0.232846	-4.582907	1.301583
H	0.615218	-5.411866	1.632547
N	1.63591	1.465865	-1.477364
N	2.833523	-0.795423	-1.607941
N	-2.849377	-1.861954	0.073713
N	-1.628303	-0.432408	1.827671
N	0.118545	-2.094322	-2.495025
N	0.794098	-1.285019	1.763597
N	-0.082167	-3.385481	-0.563521
N	-0.803118	0.920189	-2.088749
C	-0.549971	2.256195	-1.889452
C	-5.030481	-0.896738	0.512079
H	-5.611056	-0.171855	1.072953
C	2.849991	-0.423642	3.549289
C	-3.46303	-2.685365	-0.802895
H	-2.825433	-3.403753	-1.305041
C	3.653904	0.267829	-1.29991
C	5.654223	-1.141722	-1.292008
C	-4.831962	-2.664691	-1.063999
H	-5.227263	-3.377428	-1.77944
C	-1.545958	3.225297	-2.055783
H	-1.287628	4.264285	-1.890672
C	3.426216	-2.00246	-1.761493
H	2.764778	-2.819186	-2.032295
C	2.765876	3.98837	-1.005238
C	-2.734561	1.469912	3.569552

C	3.075014	-1.388724	2.552729
H	4.04689	-1.852492	2.425746
C	0.368275	-4.488274	-0.026808
C	-3.520215	0.806523	2.603659
H	-4.580502	1.021673	2.527834
C	-3.65682	-0.963207	0.744782
C	-2.847314	2.879497	-2.445533
C	3.545666	2.819719	-0.97973
H	4.608818	2.881226	-0.782716
C	4.791452	-2.21147	-1.618477
H	5.176228	-3.214305	-1.779197
C	1.54605	0.101535	3.600577
H	1.292226	0.852053	4.341656
C	-0.72375	-4.370133	-2.838842
H	-1.294589	-5.092235	-2.249647
H	-1.387965	-3.953047	-3.602836
H	0.066872	-4.914815	-3.367064
C	-3.079243	1.501418	-2.638905
H	-4.05306	1.130628	-2.944006
C	-4.453079	3.802782	-4.13539
C	-3.374985	2.504549	4.506704
C	-2.957759	-0.140722	1.74881
C	-0.839114	0.167256	2.75805
C	5.035204	0.105586	-1.135697
H	5.630342	0.979544	-0.899318
C	1.062605	-5.64121	-0.708777
H	1.92484	-5.95111	-0.106066
H	0.390601	-6.504747	-0.789447
H	1.424581	-5.385222	-1.703353
C	2.967192	1.567973	-1.222424

C	-5.671772	-1.750264	-0.403945
C	-0.166454	-3.249143	-1.975736
C	-1.37429	1.120581	3.630561
H	-0.72799	1.584994	4.364931
C	3.348427	5.387411	-0.755976
C	-3.964611	3.905456	-2.668018
C	7.417942	-2.402654	-0.026978
H	8.493067	-2.587442	0.072487
H	7.04826	-2.041394	0.940531
H	6.938079	-3.364691	-0.238412
C	0.84621	2.57491	-1.531317
C	4.858732	5.344389	-0.454053
C	-4.492498	1.817222	5.331762
H	-5.282363	1.403284	4.695266
H	-4.090215	1.00184	5.943535
H	-4.960462	2.545155	6.003665
C	3.925121	0.045143	4.537326
C	0.549086	-0.327924	2.724602
C	1.391867	3.835295	-1.294578
H	0.748985	4.707248	-1.344701
C	-2.059186	0.577641	-2.448997
H	-2.238373	-0.482797	-2.588577
C	-3.98634	3.647638	3.657924
H	-4.757638	3.284102	2.969676
H	-4.456067	4.38856	4.314159
H	-3.216732	4.15827	3.067084
C	-7.186888	-1.672678	-0.627536
C	-7.672742	-2.710082	-1.657843
H	-7.473512	-3.737702	-1.331878
H	-8.755738	-2.616177	-1.786894

H	-7.214582	-2.560674	-2.643049
C	7.164221	-1.364147	-1.148664
C	3.12535	6.2598	-2.01749
H	3.625897	5.829116	-2.892092
H	2.062954	6.374656	-2.259203
H	3.534649	7.262689	-1.853193
C	7.913589	-0.065254	-0.795714
H	8.983369	-0.276198	-0.698918
H	7.804439	0.697622	-1.575714
H	7.579202	0.358723	0.159244
C	4.120708	1.574854	4.388321
H	4.450872	1.835258	3.37552
H	3.201061	2.131858	4.598407
H	4.883454	1.922833	5.093613
C	-3.492589	5.34856	-2.407049
H	-4.323348	6.040744	-2.577822
H	-3.157511	5.493051	-1.372579
H	-2.681926	5.644482	-3.083493
C	3.458979	-0.278959	5.97959
H	4.215503	0.053906	6.698803
H	2.519591	0.224545	6.232895
H	3.312538	-1.356287	6.117072
C	-5.13884	3.588338	-1.707999
C	-2.353216	3.11599	5.484645
H	-1.544336	3.641198	4.962005
H	-2.855123	3.849319	6.123902
H	-1.91252	2.36024	6.1458
C	5.278204	-0.649364	4.291536
H	5.672054	-0.43898	3.289369
H	6.013814	-0.282126	5.014392

H	5.212243	-1.736187	4.419732
C	7.719879	-1.906481	-2.490327
H	8.799128	-2.07366	-2.404181
H	7.260683	-2.859971	-2.773042
H	7.551943	-1.194789	-3.3065
C	2.044411	-1.777625	1.698017
H	2.225598	-2.511463	0.921227
C	-7.551418	-0.256852	-1.141136
H	-7.255641	0.528185	-0.435981
H	-7.072011	-0.049478	-2.105484
H	-8.635034	-0.180443	-1.282473
C	-7.909657	-1.936944	0.717921
H	-7.646213	-1.197686	1.482464
H	-8.994266	-1.886603	0.572203
H	-7.66723	-2.930878	1.110478
C	2.624343	6.030487	0.45362
H	2.769564	5.437705	1.364443
H	3.024262	7.033996	0.636001
H	1.54673	6.133298	0.284153
H	-0.045331	-2.095282	-3.504684
H	-3.642314	4.020378	-4.839803
H	-4.847554	2.808425	-4.371068
H	-5.256369	4.526848	-4.310691
H	5.083532	4.75957	0.446375
H	5.435368	4.935384	-1.292309
H	5.223762	6.361365	-0.278498
H	-5.952394	4.304582	-1.867365
H	-5.545684	2.584388	-1.873111
H	-4.823543	3.66164	-0.660114

Table S6. Optimized structure of **TS1** at the B3LYP level

Atom	X	Y	X
C	-0.359644	4.728774	-0.093001
C	0.35716	3.592138	-0.463387
N	-0.106281	2.73443	-1.440424
C	-1.27941	3.046457	-2.024272
C	-2.025507	4.179092	-1.700712
C	-1.579405	5.068927	-0.708738
C	1.686236	3.249424	0.087602
N	2.231829	2.154244	-0.507229
C	3.492931	1.747024	-0.211582
C	4.239475	2.42171	0.761072
C	3.704467	3.537259	1.429699
C	2.40329	3.944422	1.060395
Ir	1.210387	1.200819	-1.88856
N	0.260506	0.332644	-3.491446
C	-0.207018	-0.858367	-3.724315
C	-0.858225	-1.188533	-5.052824
C	3.941538	0.628933	-1.065289
N	3.0058	0.184775	-1.971051
C	3.397713	-0.767382	-2.846313
C	4.675716	-1.311534	-2.865299
C	5.652106	-0.886971	-1.938588
C	5.237553	0.101346	-1.033492
C	4.482097	4.321922	2.496441
C	4.662863	5.78406	2.014031
C	7.070648	-1.466515	-1.9701
C	7.968893	-0.869721	-0.870072
C	-2.329734	6.345804	-0.31248
C	-2.665263	6.290239	1.199196

N	-0.202177	-1.770358	-2.646926
C	-0.074969	-3.071467	-2.830284
O	-0.222492	-3.859779	-1.787937
Ir	-0.51197	-0.977473	-0.637916
C	-2.380492	-2.837176	0.294257
I	-1.559651	-4.305005	1.576826
N	1.133341	-1.707826	0.416897
C	2.139158	-2.499886	0.004076
C	3.140425	-2.980672	0.847093
C	3.151813	-2.638745	2.209244
C	2.11272	-1.787271	2.628229
C	1.12793	-1.345739	1.746279
C	-0.00587	-0.493725	2.157444
N	-0.868102	-0.242475	1.144245
C	-1.999996	0.488384	1.337936
C	-2.277946	1.013603	2.599597
C	-1.407689	0.785527	3.686929
C	-0.257813	0.016532	3.435372
C	4.192863	-3.157944	3.207409
C	3.465633	-3.992099	4.293706
C	-2.853449	0.568979	0.132705
N	-2.350181	-0.060597	-0.980122
C	-3.136793	-0.093186	-2.076014
C	-4.403038	0.475471	-2.13004
C	-4.944674	1.127941	-1.004541
C	-4.118209	1.164776	0.128367
C	-1.750822	1.341525	5.076143
C	-1.869732	2.884064	4.991482
C	-6.362975	1.70417	-1.036976
C	-6.765881	2.333657	0.309886

C	5.249783	-4.049653	2.528609
C	4.909824	-1.955513	3.870998
C	-0.679171	0.989852	6.12579
C	-3.104417	0.73934	5.533952
C	-7.352131	0.554621	-1.363282
C	-6.443172	2.788889	-2.140473
C	3.679186	4.308489	3.821712
C	5.875207	3.720203	2.763199
C	7.70405	-1.154584	-3.350647
C	6.995642	-3.001404	-1.771576
C	-3.641033	6.514427	-1.103066
C	-1.420091	7.56881	-0.597085
C	0.289976	-3.824944	-4.080392
I	-5.164471	-3.21992	0.263473
H	-0.505222	-3.294477	-1.009042
H	-4.475804	1.627767	1.039717
H	-2.739668	-0.624447	-2.932987
H	-4.972711	0.369057	-3.047949
H	0.056322	5.372885	0.674442
H	2.651687	-1.077909	-3.570178
H	3.89433	-3.633462	0.421986
H	-3.188007	1.584326	2.74509
H	5.243973	2.085571	0.98625
H	4.903862	-2.058075	-3.620189
H	2.049881	-1.479552	3.666324
H	-1.746347	-1.813894	-4.918343
H	-1.160804	-0.265889	-5.556732
H	-0.179124	-1.717809	-5.72804
H	-2.949079	4.353173	-2.241183
H	5.936454	0.493482	-0.304408

H	0.717358	-4.785801	-3.785863
H	-0.605248	-4.034599	-4.67646
H	1.004631	-3.279313	-4.699392
H	0.434407	-0.200383	4.238953
H	8.002379	-3.430496	-1.814721
H	6.564488	-3.255858	-0.795431
H	6.397654	-3.491526	-2.547587
H	-3.925795	1.000959	4.85807
H	-3.05427	-0.353472	5.595304
H	-3.362059	1.122592	6.527228
H	1.957353	4.816449	1.525854
H	-1.628831	2.358989	-2.787294
H	-2.64759	3.202643	4.288595
H	-2.132625	3.289186	5.974561
H	-0.921551	3.339409	4.681077
H	-6.189017	2.392675	-3.129753
H	-7.46362	3.18247	-2.19807
H	-5.771357	3.62944	-1.924693
H	5.225018	5.826298	1.074325
H	3.704383	6.291199	1.857702
H	5.217583	6.356418	2.765449
H	8.964253	-1.321268	-0.927782
H	8.099649	0.212711	-0.986869
H	7.580141	-1.070345	0.1362
H	5.437132	-1.346698	3.12651
H	4.21469	-1.304846	4.413085
H	5.648526	-2.318207	4.59379
H	-3.190952	7.204278	1.49563
H	-3.31507	5.437792	1.430975
H	-1.76681	6.21283	1.821629

H	4.194714	-4.373491	5.016834
H	2.730427	-3.398606	4.848288
H	2.945696	-4.850497	3.853723
H	0.303891	1.404305	5.869412
H	-0.966086	1.413818	7.093179
H	-0.580272	-0.093012	6.266923
H	5.811349	-3.508423	1.756772
H	5.972656	-4.391933	3.275871
H	4.805706	-4.943543	2.075597
H	8.719615	-1.562962	-3.391722
H	7.137223	-1.598772	-4.175949
H	7.76682	-0.074493	-3.524817
H	2.137282	-2.770235	-1.04513
H	-6.774389	1.595129	1.12011
H	-6.107548	3.164796	0.594314
H	-7.780042	2.737863	0.232736
H	-7.279514	-0.254834	-0.629198
H	-8.37717	0.940776	-1.35262
H	-7.172321	0.124276	-2.354226
H	3.543943	3.28597	4.193888
H	4.217558	4.877416	4.587393
H	2.689026	4.764593	3.711409
H	0.153902	0.942581	-4.30376
H	-0.486409	7.533061	-0.024856
H	-1.163676	7.634836	-1.660375
H	-1.941774	8.490663	-0.317862
H	5.816857	2.687054	3.1275
H	6.512742	3.743914	1.87136
H	6.382569	4.306836	3.535497
H	-4.143334	7.433275	-0.784458

H	-3.46447	6.599775	-2.18157
H	-4.338104	5.685681	-0.927172
H	-2.754821	-1.961168	0.783139
H	-2.593408	-3.123741	-0.721379

Table S7. Optimized structure of **INT1** at the B3LYP level

Atom	X	Y	Z
C	2.336884	-2.105151	0.72687
N	1.365817	-1.208384	0.964609
C	1.587133	-0.282435	1.957487
C	2.774043	-0.267393	2.6862
C	3.789781	-1.216516	2.460481
C	3.535547	-2.144732	1.438809
Ir	-0.559771	-1.070727	0.145544
N	-0.560321	-2.609298	-1.424692
C	-0.985321	-2.158676	-2.69089
C	-1.873058	-2.974399	-3.596876
C	5.061724	-1.222854	3.315186
C	6.041105	-2.331858	2.886441
C	0.462743	0.641471	2.226025
N	-0.621191	0.400885	1.455795
C	-1.781886	1.087458	1.60465
C	-1.856402	2.108052	2.55383
C	-0.749615	2.416007	3.372372
C	0.419849	1.655982	3.187151
C	-0.863608	3.516701	4.436587
C	0.450406	3.713364	5.216659
C	-2.878526	0.590279	0.741732
N	-2.531258	-0.425975	-0.123102
C	-3.512099	-1.012664	-0.828935
C	-4.850719	-0.616528	-0.744399
C	-5.234847	0.431497	0.100108
C	-4.192942	1.033531	0.843005
C	-6.689285	0.876941	0.284272
C	-7.093185	0.633224	1.762137

Ir	0.476327	0.490534	-2.076534
C	-1.514727	-2.272908	1.598872
I	-0.390443	-3.113766	3.336531
N	1.644088	1.894488	-1.328058
C	2.970649	1.649979	-1.2012
C	3.822472	2.646196	-0.710533
C	3.318501	3.916047	-0.373896
C	1.938549	4.137659	-0.58041
C	1.119677	3.120163	-1.069326
C	4.207132	5.048638	0.160233
C	3.670566	5.504295	1.540836
C	3.357049	0.314442	-1.713341
N	2.315442	-0.451673	-2.181292
C	2.624504	-1.629073	-2.764523
C	3.927198	-2.09688	-2.898271
C	5.015201	-1.341846	-2.414522
C	4.683044	-0.116954	-1.811845
C	6.454463	-1.840434	-2.580044
C	6.750738	-2.004228	-4.093625
C	-0.314128	3.216209	-1.433621
N	-0.877223	2.053575	-1.906618
C	-2.159862	2.112925	-2.324404
C	-2.913454	3.281977	-2.314674
C	-2.360581	4.490423	-1.844808
C	-1.033569	4.413101	-1.390392
C	-3.169534	5.791715	-1.864384
C	-4.432978	5.613362	-0.985516
N	-0.681067	-0.928414	-3.004774
C	-0.292291	-3.895196	-1.220928
C	-0.13414	-4.987201	-2.244763

O	0.06839	-4.308808	-0.026584
C	-1.978298	3.123388	5.440482
C	-1.232424	4.854364	3.747251
C	5.674937	4.610442	0.325806
C	4.157346	6.237584	-0.833836
C	-3.592853	6.090741	-3.325907
C	-2.361267	6.989688	-1.330459
C	5.772424	0.147527	3.182497
C	4.662364	-1.459478	4.795096
C	-7.652426	0.090183	-0.625242
C	-6.811423	2.38477	-0.046483
C	6.59803	-3.212435	-1.873693
C	7.483029	-0.863976	-1.978737
H	-0.156323	-3.664729	0.677856
H	-4.421105	1.828712	1.544557
H	-3.248697	-1.876122	-1.425994
H	-5.574223	-1.185802	-1.316858
H	-0.5366	5.303342	-1.024482
H	1.787277	-2.198941	-3.1516
H	4.247217	-2.924711	1.193549
H	-2.786887	2.651103	2.673216
H	4.880316	2.439117	-0.607961
H	4.079767	-3.049652	-3.396121
H	2.892186	0.475401	3.46728
H	-2.599731	-3.549445	-2.999749
H	-2.407543	-2.308405	-4.281483
H	-1.307748	-3.68424	-4.206342
H	-3.930984	3.23508	-2.690054
H	5.467343	0.52915	-1.436282
H	0.48424	-5.771349	-1.803331

H	-1.116987	-5.420477	-2.456088
H	0.323054	-4.629303	-3.170129
H	1.288266	1.839434	3.806544
H	7.61723	-3.590677	-2.00554
H	6.409077	-3.128248	-0.796358
H	5.914868	-3.964466	-2.282743
H	-2.956515	3.024206	4.957452
H	-1.74935	2.174685	5.938523
H	-2.069918	3.896018	6.211376
H	1.510389	5.112235	-0.374103
H	-2.585518	1.182866	-2.686095
H	-2.185112	4.795949	3.209314
H	-1.331088	5.643122	4.500567
H	-0.455758	5.16215	3.036444
H	-7.647675	-0.982132	-0.40041
H	-8.674581	0.44895	-0.468032
H	-7.419807	0.224358	-1.688593
H	4.525576	5.945043	-1.823576
H	3.144384	6.637404	-0.953545
H	4.789662	7.052922	-0.466362
H	8.490683	-1.269094	-2.113534
H	7.461763	0.114634	-2.473218
H	7.335287	-0.717879	-0.901144
H	6.074746	0.340229	2.145728
H	5.139747	0.978072	3.515285
H	6.675282	0.156385	3.802278
H	-2.981573	7.890666	-1.365226
H	-2.053782	6.850344	-0.286414
H	-1.470101	7.189678	-1.937521
H	5.560919	-1.471915	5.42131

H	4.004066	-0.671987	5.17843
H	4.149993	-2.419686	4.919843
H	1.280423	4.00886	4.562422
H	0.319016	4.512685	5.952545
H	0.740277	2.812741	5.770704
H	6.373434	-2.21093	1.847651
H	6.933676	-2.293867	3.518711
H	5.60719	-3.33176	3.000725
H	7.777063	-2.361122	-4.2307
H	6.084006	-2.730741	-4.570136
H	6.65085	-1.051135	-4.625226
H	2.140688	-2.834063	-0.049654
H	-6.164989	3.005712	0.584517
H	-6.559932	2.580988	-1.095971
H	-7.842696	2.715394	0.116351
H	-6.480128	1.212268	2.462452
H	-8.135881	0.933318	1.911923
H	-7.005483	-0.425971	2.027469
H	3.703066	4.685713	2.269983
H	4.288478	6.322311	1.926133
H	2.639914	5.872094	1.484828
H	-1.085329	-0.666322	-3.904364
H	-2.719866	6.21704	-3.976203
H	-4.218341	5.296693	-3.747473
H	-4.174049	7.018505	-3.357874
H	5.781524	3.77716	1.031559
H	6.130961	4.324195	-0.629393
H	6.260627	5.445797	0.721891
H	-5.031239	6.530367	-1.009983
H	-5.070088	4.795542	-1.337931

H	-4.165692	5.413865	0.05937
H	-2.259118	-1.669663	2.119783
H	-2.034705	-3.131379	1.152072
I	-3.718259	-4.621451	-0.671335

Table S8. Optimized structure of **3A** at the B3LYP level

Atom	X	Y	Z
Ir	-0.237796	-1.466742	-0.91969
Ir	0.13486	1.344088	-1.247907
C	-0.689839	-3.595321	-0.933126
O	1.605872	-3.332151	-2.872039
H	1.117634	-3.393494	-2.016114
N	0.631113	1.899993	0.57642
N	2.201038	1.25262	-1.372326
N	-2.296963	-1.349118	-1.243253
N	-0.874009	-1.416889	0.934455
N	-0.328907	0.774899	-3.168621
N	1.573093	-1.617011	0.106643
N	0.380304	-1.415485	-3.028204
N	-1.737467	1.833053	-0.468878
C	-1.679716	2.331946	0.808833
C	-4.412485	-1.49375	-0.081211
H	-4.932732	-1.526744	0.869822
C	3.794206	-2.066341	1.845572
C	-2.984421	-1.456392	-2.395415
H	-2.397412	-1.467502	-3.30566
C	2.838256	1.643286	-0.220364
C	5.031691	1.583887	-1.300637
C	-4.370375	-1.58388	-2.457489
H	-4.831926	-1.680529	-3.433872
C	-2.797217	2.913275	1.419547
H	-2.687815	3.314544	2.419516
C	2.964472	1.047449	-2.465856
H	2.430932	0.797597	-3.375936
C	1.354506	2.831593	3.110488

C	-1.800643	-1.499421	3.567485
C	3.931502	-2.00196	0.448841
H	4.893765	-2.13835	-0.031219
C	1.20678	-2.286405	-3.569071
C	-2.689458	-1.398502	2.476138
H	-3.758689	-1.35899	2.650754
C	-3.021198	-1.376175	-0.072073
C	-4.019104	3.038616	0.74585
C	2.32264	2.430035	2.17423
H	3.374601	2.486777	2.424098
C	4.345418	1.192472	-2.470441
H	4.874337	1.026011	-3.404002
C	2.489505	-1.877419	2.332267
H	2.297966	-1.920663	3.398749
C	-0.570962	-0.505295	-5.239032
H	-0.96527	-1.509155	-5.424624
H	-1.352776	0.223867	-5.469989
H	0.247941	-0.331903	-5.943229
C	-4.049641	2.508763	-0.561505
H	-4.944824	2.566946	-1.172978
C	-5.578538	4.971471	0.450699
C	-2.345631	-1.55227	5.002194
C	-2.213819	-1.37313	1.165425
C	0.019325	-1.543368	1.945166
C	4.229303	1.800282	-0.172981
H	4.679453	2.130097	0.755313
C	1.871518	-2.237512	-4.920936
H	2.771049	-2.854883	-4.87777
H	1.217195	-2.664212	-5.688697
H	2.139856	-1.220948	-5.213945

C	1.945108	1.979683	0.903748
C	-5.138164	-1.616556	-1.279921
C	-0.136322	-0.338841	-3.796674
C	-0.429487	-1.577463	3.269712
H	0.292178	-1.68355	4.06942
C	1.714647	3.353069	4.509698
C	-5.240278	3.748106	1.341918
C	7.238636	0.469164	-1.742152
H	8.323949	0.611688	-1.778783
H	7.030952	-0.339494	-1.030272
H	6.918801	0.138819	-2.736377
C	-0.33885	2.327749	1.426892
C	3.232503	3.318377	4.772352
C	-3.272002	-2.787533	5.141817
H	-4.130717	-2.741858	4.462861
H	-2.728903	-3.717277	4.938594
H	-3.664821	-2.842082	6.162831
C	4.959183	-2.346649	2.80172
C	1.409344	-1.672287	1.473926
C	0.004755	2.780778	2.700255
H	-0.77771	3.119666	3.370023
C	-2.916547	1.929646	-1.118207
H	-2.932083	1.551004	-2.134206
C	-3.154369	-0.261554	5.285488
H	-3.997442	-0.140265	4.596182
H	-3.563956	-0.295749	6.300709
H	-2.518813	0.628965	5.208161
C	-6.658094	-1.817539	-1.268485
C	-7.236158	-1.93546	-2.691889
H	-6.831669	-2.799693	-3.231651

H	-8.320508	-2.072004	-2.633151
H	-7.055601	-1.033158	-3.288647
C	6.550445	1.789041	-1.312569
C	1.227395	4.819115	4.639245
H	1.700377	5.462528	3.889085
H	0.141654	4.907005	4.522152
H	1.483599	5.209405	5.630259
C	7.093581	2.202558	0.068357
H	8.179827	2.324086	0.010998
H	6.679899	3.161125	0.403408
H	6.892302	1.444764	0.836412
C	5.084943	-1.17818	3.811151
H	5.300749	-0.23231	3.299362
H	4.17525	-1.045008	4.40742
H	5.90612	-1.377624	4.507914
C	-4.984424	4.241972	2.778689
H	-5.885906	4.730915	3.16126
H	-4.746794	3.418235	3.463781
H	-4.175439	4.980769	2.822022
C	4.667911	-3.66367	3.566619
H	5.492317	-3.881869	4.254153
H	3.749555	-3.602889	4.161112
H	4.569126	-4.509568	2.877344
C	-6.443376	2.773998	1.360642
C	-1.220904	-1.664791	6.049268
H	-0.538251	-0.806346	6.016949
H	-1.659409	-1.690862	7.051748
H	-0.636957	-2.585186	5.930295
C	6.29715	-2.498242	2.053307
H	6.567157	-1.588872	1.501857

H	7.098291	-2.690409	2.773959
H	6.283938	-3.340934	1.352516
C	6.890351	2.906046	-2.333138
H	7.973239	3.070131	-2.353924
H	6.576586	2.646436	-3.349838
H	6.408843	3.852275	-2.062404
C	2.823886	-1.776283	-0.364947
H	2.945791	-1.726709	-1.439138
C	-7.328035	-0.614903	-0.559638
H	-6.977702	-0.490366	0.470937
H	-7.138054	0.319517	-1.100539
H	-8.412098	-0.766108	-0.522228
C	-6.979364	-3.121949	-0.493604
H	-6.641228	-3.080655	0.547901
H	-8.062333	-3.285224	-0.480873
H	-6.512973	-3.992703	-0.968026
C	1.011795	2.477613	5.577138
H	1.347956	1.434944	5.518919
H	1.247735	2.851494	6.579293
H	-0.078896	2.491707	5.47143
H	-0.710489	1.519052	-3.759314
H	-4.740748	5.676252	0.408079
H	-5.825375	4.679051	-0.575535
H	-6.445814	5.499521	0.861905
H	3.641505	2.302339	4.704514
H	3.7849	3.966614	4.081951
H	3.435029	3.682271	5.78462
H	-7.325471	3.283194	1.763498
H	-6.700814	2.416351	0.358243
H	-6.240042	1.901739	1.994021

I	0.238485	4.015996	-2.325642
I	0.61533	-5.099035	0.106103
H	-1.619897	-3.736606	-0.378555
H	-0.842519	-4.042385	-1.921565

Table S9. Optimized structure of **TS2A** at the B3LYP level

Atom	X	Y	Z
Ir	-0.52524	-0.24923	-0.63346
Ir	2.062639	0.58929	-1.40724
C	-3.6467	-0.79088	0.042124
O	-1.91559	-2.53449	-2.2158
H	-1.87763	-1.99816	-1.36212
N	3.022271	0.397717	0.305709
N	2.843638	-1.3208	-1.62213
N	-1.46122	1.580964	-0.83053
N	-0.61306	0.291456	1.237879
N	1.082618	0.725421	-3.21324
N	0.189825	-1.96758	0.296684
N	-0.57513	-0.79747	-2.75285
N	1.779336	2.487687	-0.59139
C	2.427267	2.664012	0.605788
C	-2.35066	3.443142	0.448984
H	-2.47087	3.90637	1.422913
C	0.997631	-4.17371	1.931623
C	-2.00681	2.157343	-1.92574
H	-1.87979	1.621572	-2.85896
C	3.62384	-1.71421	-0.56294
C	4.346501	-3.73817	-1.72927
C	-2.72915	3.343748	-1.89725
H	-3.14171	3.711238	-2.8302
C	2.532101	3.925922	1.201646
H	3.078567	4.012699	2.132624
C	2.825151	-2.11443	-2.7124
H	2.241143	-1.75337	-3.55168
C	4.500148	0.16752	2.6626

C	-0.68836	1.108053	3.917211
C	0.912391	-4.26345	0.527945
H	1.140499	-5.18586	0.00611
C	-1.23444	-1.87741	-3.13168
C	-1.14515	1.94644	2.878014
H	-1.54206	2.928857	3.110393
C	-1.63453	2.242378	0.375723
C	1.999344	5.069911	0.593326
C	4.432447	-0.88709	1.736056
H	4.965411	-1.81033	1.92504
C	3.538519	-3.30241	-2.80172
H	3.478018	-3.86737	-3.72699
C	0.66502	-2.92532	2.475807
H	0.692422	-2.7769	3.550141
C	-0.44606	0.301352	-5.07254
H	-1.53995	0.332674	-5.09413
H	-0.06434	1.25758	-5.44066
H	-0.1192	-0.47061	-5.77536
C	1.321927	4.852502	-0.62508
H	0.877441	5.674194	-1.1781
C	2.96003	7.334208	0.137287
C	-0.75147	1.585841	5.376271
C	-1.11157	1.530079	1.547305
C	-0.17337	-0.55895	2.209619
C	4.360378	-2.9048	-0.60359
H	4.975675	-3.15979	0.250535
C	-1.28822	-2.54222	-4.48142
H	-1.59636	-3.57964	-4.3372
H	-2.03439	-2.05995	-5.12227
H	-0.32247	-2.51832	-4.99004

C	3.698183	-0.75127	0.552028
C	-2.93633	4.031116	-0.68211
C	0.070306	0.056871	-3.66977
C	-0.20494	-0.16153	3.54886
H	0.141214	-0.85005	4.309541
C	5.304381	0.078168	3.968225
C	2.169746	6.48252	1.164364
C	4.21848	-6.21651	-2.11554
H	4.799111	-7.13938	-2.22011
H	3.508787	-6.35812	-1.291
H	3.645241	-6.08403	-3.03951
C	3.096606	1.462831	1.14463
C	5.96727	-1.30054	4.152146
C	-2.22524	1.888795	5.74801
H	-2.65783	2.673958	5.117869
H	-2.84988	0.993534	5.649904
H	-2.28638	2.231615	6.786845
C	1.396621	-5.34801	2.834684
C	0.25734	-1.85462	1.673888
C	3.818843	1.359235	2.332824
H	3.869874	2.214496	2.997364
C	1.236275	3.577759	-1.17091
H	0.739301	3.413324	-2.12066
C	0.094245	2.87484	5.52884
H	-0.26348	3.683203	4.881304
H	0.044386	3.235623	6.56213
H	1.147369	2.686133	5.287378
C	-3.76438	5.31862	-0.57043
C	-4.32781	5.763797	-1.93393
H	-4.99933	5.013352	-2.36795

H	-4.90953	6.682318	-1.80614
H	-3.53398	5.980986	-2.65859
C	5.170583	-5.0257	-1.84048
C	6.415948	1.158432	3.948933
H	7.097578	1.011486	3.103753
H	6.007183	2.172489	3.879663
H	7.003673	1.103422	4.871842
C	5.969648	-5.31938	-0.5566
H	6.530277	-6.2512	-0.68143
H	6.699623	-4.53132	-0.3367
H	5.31723	-5.44776	0.316525
C	2.635585	-4.95114	3.675456
H	3.494425	-4.72374	3.032067
H	2.443073	-4.07773	4.308559
H	2.919596	-5.77834	4.335204
C	2.939473	6.483317	2.499078
H	3.028608	7.510374	2.867135
H	2.423026	5.904279	3.275068
H	3.957698	6.092296	2.38717
C	0.21419	-5.67649	3.781759
H	0.480626	-6.51558	4.433999
H	-0.04749	-4.82845	4.424312
H	-0.67989	-5.95983	3.214772
C	0.776409	7.11545	1.396984
C	-0.21025	0.5302	6.359614
H	0.842521	0.288637	6.16704
H	-0.27156	0.918041	7.381512
H	-0.79425	-0.39743	6.330866
C	1.738854	-6.61182	2.02271
H	2.583258	-6.44761	1.341634

H	2.023869	-7.41913	2.705011
H	0.883842	-6.969	1.437117
C	6.166138	-4.87589	-3.01974
H	6.767905	-5.78649	-3.11365
H	5.654149	-4.71864	-3.97509
H	6.848009	-4.03328	-2.85962
C	0.523819	-3.16543	-0.23107
H	0.471617	-3.24403	-1.31
C	-2.87211	6.454687	-0.01323
H	-2.45835	6.211096	0.971455
H	-2.03675	6.667517	-0.69053
H	-3.46036	7.372719	0.09343
C	-4.95076	5.075085	0.396685
H	-4.6151	4.80102	1.403112
H	-5.55046	5.987362	0.487818
H	-5.60703	4.277449	0.029221
C	4.357153	0.330896	5.167776
H	3.56532	-0.42679	5.213472
H	4.922562	0.284845	6.104888
H	3.882707	1.317503	5.119531
H	1.507271	1.351441	-3.90134
H	3.949941	6.906227	-0.05617
H	2.435599	7.416686	-0.82072
H	3.099058	8.348657	0.52695
H	5.230327	-2.11281	4.186505
H	6.692864	-1.51761	3.359373
H	6.51352	-1.31806	5.100592
H	0.892166	8.13347	1.784457
H	0.193304	7.180174	0.47262
H	0.195329	6.540435	2.128293

I	4.328451	1.622761	-2.77245
I	-3.67644	-2.64019	1.091259
H	-3.18188	0.001745	0.605178
H	-3.53509	-0.86243	-1.03015
C	-5.64882	0.125215	-0.2372
C	-5.9393	-0.1828	-1.66906
C	-6.36172	-0.26922	0.898262
H	-5.13203	1.077271	-0.10923
C	-7.47463	-1.16901	1.116652
H	-5.9852	0.177181	1.820405
C	-6.09851	0.887758	-2.57111
C	-6.35364	0.655517	-3.9302
C	-6.42735	-0.65731	-4.41707
C	-6.24167	-1.73233	-3.53259
C	-5.99988	-1.49792	-2.17374
H	-6.05213	1.910902	-2.19962
H	-6.50613	1.495899	-4.60449
H	-6.6356	-0.84142	-5.46889
H	-6.29798	-2.75512	-3.89987
H	-5.86067	-2.34111	-1.5006
C	-7.76554	-1.51929	2.464736
C	-8.84137	-2.34666	2.784342
C	-9.6751	-2.8348	1.764858
C	-9.42122	-2.48052	0.427864
C	-8.33887	-1.66411	0.102088
H	-7.12969	-1.13356	3.26026
H	-9.04038	-2.60504	3.821845
H	-10.5215	-3.47287	2.009089
H	-10.0808	-2.83882	-0.35942
H	-8.18014	-1.38325	-0.93234

Table S10. Optimized structure of **INT2A** at the B3LYP level

Atom	X	Y	Z
Ir	-0.22663	0.227486	-0.84067
Ir	2.562249	-0.02355	-1.33216
C	-5.59162	1.612445	0.66428
O	-2.45199	-0.79663	-2.63458
H	-2.176	-0.42227	-1.73896
N	3.197397	-0.51216	0.472668
N	2.585706	-2.09286	-1.48608
N	-0.38798	2.281414	-0.90547
N	-0.37017	0.615591	1.06504
N	1.898083	0.428736	-3.23216
N	-0.40687	-1.69428	-0.07135
N	-0.32509	-0.1194	-2.99758
N	2.92312	1.867926	-0.52518
C	3.452804	1.823482	0.739644
C	-0.69445	4.240376	0.49154
H	-0.77256	4.641836	1.495529
C	-0.87682	-4.14372	1.324868
C	-0.50883	3.101075	-1.97586
H	-0.44078	2.624845	-2.94815
C	3.044517	-2.72004	-0.35488
C	3.087378	-4.89475	-1.47203
C	-0.73192	4.466852	-1.87735
H	-0.83093	5.036248	-2.79625
C	3.936571	2.976136	1.368718
H	4.365946	2.882966	2.359096
C	2.392202	-2.84759	-2.58656
H	2.071541	-2.31568	-3.47651
C	4.226552	-1.21016	2.969395

C	-0.56883	1.192155	3.796663
C	-0.77359	-4.08292	-0.07887
H	-0.88986	-4.96929	-0.69258
C	-1.44836	-0.63442	-3.47086
C	-0.51861	2.218073	2.828021
H	-0.56797	3.256868	3.137028
C	-0.4779	2.864446	0.343345
C	3.92277	4.221671	0.725889
C	3.862234	-2.18971	2.029087
H	3.986757	-3.23972	2.263197
C	2.621777	-4.217	-2.6189
H	2.459007	-4.74076	-3.55631
C	-0.71615	-2.92134	1.996533
H	-0.79386	-2.88216	3.078261
C	0.576569	0.69778	-5.26254
H	-0.31193	1.323539	-5.39852
H	1.45084	1.268203	-5.58741
H	0.484877	-0.16427	-5.92908
C	3.361217	4.234825	-0.5681
H	3.310287	5.148296	-1.15295
C	5.690159	5.968492	0.443459
C	-0.6886	1.546808	5.286329
C	-0.43043	1.920088	1.469196
C	-0.44853	-0.40806	1.959614
C	3.282864	-4.09961	-0.33424
H	3.660161	-4.54449	0.578797
C	-1.7673	-1.15353	-4.84892
H	-2.62818	-1.81961	-4.76555
H	-2.03856	-0.339	-5.52609
H	-0.92625	-1.7027	-5.28188

C	3.359729	-1.82281	0.775155
C	-0.84377	5.089483	-0.61317
C	0.745738	0.291519	-3.81292
C	-0.5404	-0.13382	3.32703
H	-0.61005	-0.95756	4.026997
C	4.80741	-1.55649	4.348248
C	4.51779	5.493602	1.340699
C	2.121441	-7.16543	-1.95139
H	2.338101	-8.23789	-2.00691
H	1.305865	-7.02447	-1.23119
H	1.762868	-6.85307	-2.93833
C	3.571712	0.472591	1.328683
C	4.87816	-3.0769	4.586684
C	-1.99155	2.356553	5.508771
H	-1.99721	3.295289	4.943201
H	-2.87311	1.777089	5.210725
H	-2.09539	2.612127	6.569357
C	-1.16659	-5.43544	2.09937
C	-0.49993	-1.72449	1.308056
C	4.075582	0.140377	2.585687
H	4.37292	0.93282	3.263866
C	2.884693	3.064303	-1.14466
H	2.474618	3.067833	-2.14884
C	0.532188	2.402922	5.707237
H	0.603813	3.333105	5.131904
H	0.450033	2.676977	6.765028
H	1.468665	1.847249	5.57406
C	-1.17563	6.580859	-0.48176
C	-0.36333	7.409803	-1.50377
H	-0.6108	7.153172	-2.53992

H	-0.58578	8.474471	-1.37487
H	0.716518	7.275515	-1.36459
C	3.393766	-6.39591	-1.5176
C	6.241819	-0.97635	4.446646
H	6.894177	-1.39563	3.672143
H	6.256008	0.114658	4.344773
H	6.674897	-1.22091	5.423029
C	3.857093	-6.93839	-0.15261
H	4.049147	-8.01324	-0.23384
H	4.790436	-6.46978	0.181911
H	3.096037	-6.8027	0.626817
C	0.016552	-5.72727	3.05643
H	0.950304	-5.87596	2.499792
H	0.174046	-4.91522	3.77557
H	-0.18297	-6.6402	3.628746
C	5.0529	5.258223	2.766334
H	5.455919	6.195766	3.163212
H	4.265245	4.926158	3.454715
H	5.86849	4.525266	2.78401
C	-2.46602	-5.25082	2.924172
H	-2.68428	-6.16814	3.482513
H	-2.38423	-4.43565	3.651955
H	-3.32261	-5.03851	2.274129
C	3.430125	6.593873	1.396494
C	-0.73704	0.293623	6.181726
H	0.169511	-0.31746	6.085866
H	-0.81234	0.597752	7.230947
H	-1.60946	-0.33443	5.964374
C	-1.35283	-6.64307	1.161091
H	-0.45271	-6.84964	0.568603

H	-1.56091	-7.53866	1.755767
H	-2.19707	-6.50446	0.475458
C	4.521629	-6.63491	-2.55501
H	4.759438	-7.70348	-2.60293
H	4.230752	-6.31538	-3.56168
H	5.435442	-6.09575	-2.28051
C	-0.53726	-2.87113	-0.71919
H	-0.45816	-2.83007	-1.80021
C	-0.87274	7.114837	0.933738
H	-1.48689	6.628342	1.701077
H	0.183349	6.989857	1.200568
H	-1.0973	8.185706	0.974976
C	-2.68969	6.759776	-0.77099
H	-3.30332	6.200045	-0.05541
H	-2.96112	7.818733	-0.69239
H	-2.95214	6.421642	-1.78
C	3.917131	-0.92737	5.449116
H	2.895682	-1.32541	5.40882
H	4.330322	-1.15723	6.437507
H	3.86163	0.163889	5.363342
H	2.633022	0.755045	-3.86436
H	6.477129	5.208323	0.382099
H	5.362883	6.198322	-0.57638
H	6.130865	6.879913	0.863337
H	3.888453	-3.54921	4.543531
H	5.538258	-3.57669	3.867605
H	5.286123	-3.26985	5.584149
H	3.853794	7.51337	1.814561
H	3.037293	6.836519	0.402295
H	2.589758	6.289248	2.032514

I	5.17234	0.04403	-2.42219
I	-4.42092	-0.22275	0.816029
H	-5.49313	2.080431	1.642708
H	-5.191	2.211796	-0.15471
C	-7.46067	1.37978	0.378076
C	-7.78065	1.297193	-1.10179
C	-7.98792	0.374521	1.322967
H	-7.73931	2.375466	0.745719
C	-8.36436	-0.98835	1.126346
H	-8.08195	0.734358	2.347664
C	-9.10591	1.569196	-1.49426
C	-9.47981	1.53457	-2.84284
C	-8.53058	1.231742	-3.83038
C	-7.20853	0.96544	-3.45112
C	-6.83694	0.999116	-2.09847
H	-9.85264	1.803058	-0.73746
H	-10.5097	1.745926	-3.12252
H	-8.8199	1.204418	-4.87874
H	-6.46393	0.726836	-4.20859
H	-5.80928	0.771331	-1.82794
C	-8.87958	-1.69884	2.257998
C	-9.28388	-3.02692	2.164149
C	-9.19129	-3.70896	0.935953
C	-8.68538	-3.03424	-0.19058
C	-8.27856	-1.70383	-0.10807
H	-8.96191	-1.17739	3.210645
H	-9.68004	-3.53541	3.040793
H	-9.51641	-4.74389	0.856026
H	-8.61906	-3.5552	-1.144
H	-7.90102	-1.20928	-0.99552

Table S11. Optimized structure of **TS3A(*cis*)** at the B3LYP level

Atom	X	Y	Z
Ir	-0.226634	0.227486	-0.840669
Ir	2.562249	-0.023548	-1.332158
C	-5.591624	1.612445	0.66428
O	-2.451991	-0.796631	-2.634575
H	-2.175997	-0.422267	-1.738961
N	3.197397	-0.512162	0.472668
N	2.585706	-2.092856	-1.486081
N	-0.38798	2.281414	-0.905465
N	-0.370168	0.615591	1.06504
N	1.898083	0.428736	-3.232159
N	-0.406872	-1.694283	-0.071348
N	-0.325089	-0.119401	-2.997579
N	2.92312	1.867926	-0.525184
C	3.452804	1.823482	0.739644
C	-0.694453	4.240376	0.49154
H	-0.772555	4.641836	1.495529
C	-0.876824	-4.143715	1.324868
C	-0.508831	3.101075	-1.975862
H	-0.440779	2.624845	-2.948146
C	3.044517	-2.720041	-0.354878
C	3.087378	-4.894746	-1.472034
C	-0.73192	4.466852	-1.877348
H	-0.830926	5.036248	-2.796254
C	3.936571	2.976136	1.368718
H	4.365946	2.882966	2.359096
C	2.392202	-2.847592	-2.586564
H	2.071541	-2.31568	-3.476506
C	4.226552	-1.210156	2.969395

C	-0.568834	1.192155	3.796663
C	-0.773591	-4.082918	-0.078871
H	-0.889856	-4.96929	-0.692579
C	-1.448355	-0.634423	-3.470859
C	-0.518614	2.218073	2.828021
H	-0.567969	3.256868	3.137028
C	-0.477898	2.864446	0.343345
C	3.92277	4.221671	0.725889
C	3.862234	-2.189705	2.029087
H	3.986757	-3.239722	2.263197
C	2.621777	-4.217002	-2.618902
H	2.459007	-4.740764	-3.556312
C	-0.716154	-2.921338	1.996533
H	-0.793855	-2.882155	3.078261
C	0.576569	0.69778	-5.262536
H	-0.31193	1.323539	-5.398515
H	1.45084	1.268203	-5.587414
H	0.484877	-0.164268	-5.929081
C	3.361217	4.234825	-0.568099
H	3.310287	5.148296	-1.152952
C	5.690159	5.968492	0.443459
C	-0.688596	1.546808	5.286329
C	-0.430426	1.920088	1.469196
C	-0.448534	-0.408061	1.959614
C	3.282864	-4.099609	-0.334244
H	3.660161	-4.544493	0.578797
C	-1.767295	-1.153531	-4.848919
H	-2.628175	-1.819608	-4.765545
H	-2.038559	-0.338999	-5.526092
H	-0.926253	-1.702696	-5.281883

C	3.359729	-1.822813	0.775155
C	-0.843766	5.089483	-0.613171
C	0.745738	0.291519	-3.812924
C	-0.540403	-0.133824	3.32703
H	-0.610049	-0.957563	4.026997
C	4.80741	-1.556494	4.348248
C	4.51779	5.493602	1.340699
C	2.121441	-7.165426	-1.951394
H	2.338101	-8.237894	-2.006908
H	1.305865	-7.02447	-1.231189
H	1.762868	-6.853069	-2.938334
C	3.571712	0.472591	1.328683
C	4.87816	-3.076895	4.586684
C	-1.991546	2.356553	5.508771
H	-1.997212	3.295289	4.943201
H	-2.873105	1.777089	5.210725
H	-2.095392	2.612127	6.569357
C	-1.166591	-5.435438	2.09937
C	-0.499926	-1.724494	1.308056
C	4.075582	0.140377	2.585687
H	4.37292	0.93282	3.263866
C	2.884693	3.064303	-1.144661
H	2.474618	3.067833	-2.148839
C	0.532188	2.402922	5.707237
H	0.603813	3.333105	5.131904
H	0.450033	2.676977	6.765028
H	1.468665	1.847249	5.57406
C	-1.175628	6.580859	-0.481763
C	-0.363331	7.409803	-1.503769
H	-0.610804	7.153172	-2.539917

H	-0.585783	8.474471	-1.374865
H	0.716518	7.275515	-1.364594
C	3.393766	-6.395911	-1.517596
C	6.241819	-0.976353	4.446646
H	6.894177	-1.395633	3.672143
H	6.256008	0.114658	4.344773
H	6.674897	-1.220913	5.423029
C	3.857093	-6.938394	-0.152612
H	4.049147	-8.013242	-0.233841
H	4.790436	-6.46978	0.181911
H	3.096037	-6.802696	0.626817
C	0.016552	-5.72727	3.05643
H	0.950304	-5.875956	2.499792
H	0.174046	-4.915217	3.77557
H	-0.18297	-6.640202	3.628746
C	5.0529	5.258223	2.766334
H	5.455919	6.195766	3.163212
H	4.265245	4.926158	3.454715
H	5.86849	4.525266	2.78401
C	-2.466023	-5.25082	2.924172
H	-2.684284	-6.168135	3.482513
H	-2.384231	-4.435648	3.651955
H	-3.32261	-5.038511	2.274129
C	3.430125	6.593873	1.396494
C	-0.737035	0.293623	6.181726
H	0.169511	-0.317455	6.085866
H	-0.812344	0.597752	7.230947
H	-1.60946	-0.334432	5.964374
C	-1.352828	-6.643067	1.161091
H	-0.452714	-6.849641	0.568603

H	-1.560912	-7.538661	1.755767
H	-2.197068	-6.504462	0.475458
C	4.521629	-6.634912	-2.555007
H	4.759438	-7.703482	-2.602925
H	4.230752	-6.315375	-3.561681
H	5.435442	-6.095748	-2.280514
C	-0.537256	-2.871134	-0.719189
H	-0.458161	-2.830072	-1.800212
C	-0.872739	7.114837	0.933738
H	-1.486893	6.628342	1.701077
H	0.183349	6.989857	1.200568
H	-1.097301	8.185706	0.974976
C	-2.689685	6.759776	-0.770987
H	-3.303322	6.200045	-0.055413
H	-2.961122	7.818733	-0.692394
H	-2.95214	6.421642	-1.779996
C	3.917131	-0.927373	5.449116
H	2.895682	-1.325413	5.40882
H	4.330322	-1.157226	6.437507
H	3.86163	0.163889	5.363342
H	2.633022	0.755045	-3.864364
H	6.477129	5.208323	0.382099
H	5.362883	6.198322	-0.576379
H	6.130865	6.879913	0.863337
H	3.888453	-3.549206	4.543531
H	5.538258	-3.576687	3.867605
H	5.286123	-3.269851	5.584149
H	3.853794	7.51337	1.814561
H	3.037293	6.836519	0.402295
H	2.589758	6.289248	2.032514

I	5.17234	0.04403	-2.422193
I	-4.420922	-0.222745	0.816029
H	-5.49313	2.080431	1.642708
H	-5.191004	2.211796	-0.154709
C	-7.460672	1.37978	0.378076
C	-7.780654	1.297193	-1.101793
C	-7.987923	0.374521	1.322967
H	-7.739307	2.375466	0.745719
C	-8.36436	-0.988353	1.126346
H	-8.081952	0.734358	2.347664
C	-9.105912	1.569196	-1.494257
C	-9.479811	1.53457	-2.842842
C	-8.530584	1.231742	-3.830381
C	-7.208531	0.96544	-3.451115
C	-6.836935	0.999116	-2.09847
H	-9.852635	1.803058	-0.737457
H	-10.50969	1.745926	-3.122523
H	-8.819897	1.204418	-4.878735
H	-6.46393	0.726836	-4.208593
H	-5.809279	0.771331	-1.827936
C	-8.879583	-1.698841	2.257998
C	-9.283881	-3.026917	2.164149
C	-9.191287	-3.708958	0.935953
C	-8.685383	-3.034235	-0.190577
C	-8.278556	-1.703829	-0.108067
H	-8.961912	-1.177392	3.210645
H	-9.680044	-3.535411	3.040793
H	-9.516411	-4.743893	0.856026
H	-8.61906	-3.5552	-1.144
H	-7.901019	-1.209277	-0.995524

Table S12. Optimized structure of **TS3A(*trans*)** at the B3LYP level

Atom	X	Y	Z
C	-8.58011	-0.2467	-0.54502
C	-7.38214	-0.30637	0.236612
C	-7.17679	0.737432	1.197203
C	-8.10742	1.756414	1.370036
C	-9.28129	1.789544	0.590351
C	-9.50404	0.781066	-0.36549
C	-6.41367	-1.33429	0.113113
C	-6.4754	-2.52331	-0.82372
C	-5.64421	-2.26416	-2.04991
H	-5.51615	-1.27249	0.724904
C	-6.18864	-3.82489	-0.07014
C	-7.1391	-4.2679	0.870208
C	-6.9467	-5.45427	1.586234
C	-5.79157	-6.22375	1.374882
C	-4.83803	-5.78866	0.446312
C	-5.03304	-4.59657	-0.26934
I	-3.10563	-1.26276	-1.54445
Ir	-0.21647	-0.19475	-1.19376
N	0.312101	-2.00748	-0.30635
C	-0.02807	-2.07119	1.029236
C	0.145288	-3.24016	1.771712
C	0.660709	-4.41315	1.194585
C	1.008686	-4.31564	-0.16427
C	0.827187	-3.12222	-0.8591
C	-0.64638	-0.85123	1.575977
N	-0.81494	0.119049	0.643037
C	-1.46966	1.278518	0.929531
C	-1.93155	1.50803	2.224935

C	-1.75384	0.544643	3.240402
C	-1.10344	-0.64935	2.882242
C	-1.6875	2.127724	-0.25513
N	-1.14433	1.646579	-1.42232
C	-1.41044	2.331465	-2.55511
C	-2.18974	3.479458	-2.59093
C	-2.76218	3.99723	-1.40892
C	-2.4748	3.285515	-0.23727
C	-3.66853	5.232049	-1.45196
C	-4.17164	5.634896	-0.05282
C	-2.29499	0.808083	4.653623
C	-1.63988	2.093059	5.218785
C	0.79573	-5.70057	2.016433
C	-0.60972	-6.12256	2.516545
N	0.437273	-0.46137	-3.27931
C	0.163442	-1.52152	-4.01851
O	-0.73366	-2.39694	-3.63039
Ir	2.381155	0.858125	-0.97081
N	2.018527	1.193342	-2.96901
C	1.249639	0.571205	-3.80381
C	1.131255	1.042512	-5.24053
I	4.891315	2.116681	-1.39619
N	2.759529	0.522078	0.937384
C	3.409474	-0.61468	1.284253
C	3.753528	-0.83917	2.622541
C	3.456096	0.117119	3.608764
C	2.816285	1.302376	3.185225
C	2.486842	1.495253	1.844134
C	3.759065	-1.45737	0.122986
N	3.334013	-0.98156	-1.09241

C	3.729115	-1.64574	-2.1967
C	4.519303	-2.7874	-2.1565
C	4.962276	-3.31122	-0.92347
C	4.550276	-2.6086	0.217133
C	3.838115	-0.06469	5.085371
C	4.821565	1.06334	5.490427
C	1.927422	2.716582	1.226857
N	1.723417	2.647761	-0.12816
C	1.321146	3.770594	-0.75644
C	1.107488	4.975229	-0.09917
C	1.305363	5.079188	1.293335
C	1.712296	3.902805	1.937671
C	5.867494	-4.54704	-0.87521
C	6.218719	-4.95654	0.567733
C	1.120641	6.417223	2.01805
C	1.372851	6.30028	3.533363
C	-1.99903	-0.35582	5.618837
C	-3.83124	1.000995	4.576374
C	1.389852	-6.85575	1.188303
C	1.719531	-5.43664	3.231752
C	-4.89495	4.910699	-2.34514
C	-2.88366	6.421283	-2.05963
C	2.561218	0.023149	5.957733
C	4.5175	-1.42221	5.3495
C	7.181821	-4.22695	-1.63333
C	5.147367	-5.73201	-1.56619
C	-0.32589	6.923482	1.79478
C	2.124362	7.440146	1.425798
C	0.837386	-1.95005	-5.29833
H	-1.32103	-2.04984	-2.89516

H	-2.89449	3.609619	0.707179
H	-1.00564	1.916386	-3.47029
H	-2.36409	3.948605	-3.55444
H	1.903749	3.908482	3.003668
H	3.423312	-1.21432	-3.1433
H	1.411353	-5.16344	-0.70675
H	-2.45321	2.433635	2.441108
H	4.273761	-1.75014	2.890192
H	4.805669	-3.24528	-3.09842
H	-0.15752	-3.23701	2.813193
H	0.111731	0.917485	-5.61705
H	1.396413	2.101908	-5.30083
H	1.80612	0.502435	-5.91092
H	0.805427	5.832527	-0.69262
H	4.872006	-2.93505	1.198483
H	0.778066	-3.03929	-5.35943
H	0.299302	-1.5506	-6.16528
H	1.880964	-1.63662	-5.35158
H	-0.96782	-1.43332	3.61642
H	5.794593	-6.61553	-1.55671
H	4.217367	-5.9916	-1.04528
H	4.903662	-5.51643	-2.61212
H	-4.11008	1.85127	3.94399
H	-4.32334	0.106021	4.179126
H	-4.23311	1.190838	5.577669
H	2.598151	2.085983	3.902103
H	1.186723	3.694201	-1.82961
H	-1.84567	2.971206	4.596549
H	-2.0313	2.301872	6.220351
H	-0.55195	1.978776	5.29898

H	-2.52863	6.209884	-3.07402
H	-3.53132	7.30271	-2.11693
H	-2.01574	6.682192	-1.44171
H	5.732986	1.032823	4.883084
H	4.377806	2.059143	5.382352
H	5.110137	0.944458	6.540512
H	6.853882	-5.84776	0.549083
H	6.778353	-4.17381	1.093473
H	5.326364	-5.20643	1.155811
H	2.726521	-5.14499	2.908956
H	1.328808	-4.64881	3.885497
H	1.810521	-6.34694	3.834214
H	1.218759	7.276158	4.0046
H	0.683685	5.594883	4.014764
H	2.401947	5.994747	3.756572
H	-0.53326	-7.04594	3.101132
H	-1.06689	-5.36182	3.158686
H	-1.29062	-6.30696	1.678086
H	-0.92233	-0.53288	5.733543
H	-2.39503	-0.11601	6.610768
H	-2.47744	-1.28894	5.298542
H	2.397216	-6.62432	0.820337
H	1.471063	-7.74989	1.814577
H	0.757422	-7.11649	0.331809
H	7.844478	-5.09887	-1.60785
H	7.002519	-3.97944	-2.68505
H	7.711217	-3.3849	-1.17374
H	1.09339	-3.05937	-1.90655
H	-4.78169	4.847843	0.406451
H	-3.34689	5.884743	0.626341

H	-4.80385	6.524665	-0.13562
H	-5.4675	4.066353	-1.94507
H	-5.55877	5.781138	-2.38835
H	-4.60408	4.666592	-3.37253
H	1.849985	-0.77126	5.700459
H	2.82458	-0.08992	7.014906
H	2.050891	0.986842	5.849254
H	2.567619	1.942906	-3.39651
H	3.158979	7.10611	1.56164
H	1.961524	7.606184	0.355544
H	2.010335	8.405226	1.931508
H	3.867412	-2.26789	5.09233
H	5.461163	-1.52434	4.800736
H	4.755323	-1.50871	6.414425
H	-0.46143	7.889657	2.292571
H	-0.55311	7.067588	0.732826
H	-1.06089	6.223811	2.21145
H	-5.8972	-1.37815	-2.6252
H	-5.25067	-3.103	-2.6187
H	-7.51079	-2.60982	-1.19822
H	-8.04181	-3.68164	1.037486
H	-7.69963	-5.78506	2.298542
H	-5.64581	-7.15405	1.919932
H	-3.94514	-6.38496	0.265088
H	-4.27617	-4.27793	-0.98156
H	-6.27691	0.710241	1.811143
H	-7.93641	2.527912	2.118733
H	-10.0115	2.583197	0.729723
H	-10.4094	0.801843	-0.9686
H	-8.7813	-1.00915	-1.2934

Table S13. Optimized structure of **INT3A···*cis*-cyclopropane** at the B3LYP level

Atom	X	Y	Z
Ir	-0.95266	-0.18827	-1.70644
Ir	-2.47783	1.146021	0.201862
C	9.466294	-3.03721	-0.26519
O	-0.6991	1.334924	-4.70365
H	-0.26655	0.486867	-4.36546
N	-1.22165	1.226022	1.724761
N	-1.60236	3.000076	-0.12646
N	-2.09348	-1.92851	-1.61516
N	0.121079	-1.23611	-0.44072
N	-3.81232	1.113066	-1.3652
N	0.679778	1.09081	-1.43926
N	-2.26245	0.952565	-3.06915
N	-3.00784	-0.59086	1.228269
C	-2.30773	-0.76542	2.394869
C	-2.0665	-4.18143	-0.74218
H	-1.58224	-4.90653	-0.09991
C	3.171947	2.419965	-1.0461
C	-3.15289	-2.28651	-2.36693
H	-3.54332	-1.52898	-3.0355
C	-0.6849	3.347217	0.832505
C	-0.4529	5.603927	-0.07978
C	-3.71012	-3.55936	-2.34585
H	-4.55207	-3.75966	-3.00137
C	-2.6437	-1.77554	3.302853
H	-2.06783	-1.86265	4.215951
C	-1.9486	3.937944	-1.02965
H	-2.71495	3.651147	-1.74076
C	0.424317	1.432981	3.967419

C	1.697939	-2.79388	1.252916
C	2.168027	2.936354	-1.87989
H	2.307643	3.862799	-2.42508
C	-1.87425	1.563413	-4.17743
C	0.46813	-3.26672	0.749438
H	0.122023	-4.26038	1.009812
C	-1.54089	-2.88691	-0.80609
C	-3.73022	-2.63252	3.076608
C	0.396111	2.438567	2.985746
H	1.012573	3.321588	3.096611
C	-1.40847	5.217947	-1.0427
H	-1.76231	5.915215	-1.79599
C	2.866596	1.200925	-0.41072
H	3.603082	0.723558	0.226476
C	-4.77184	1.013693	-3.60975
H	-4.52948	0.408656	-4.48811
H	-5.63833	0.57068	-3.11036
H	-5.07294	2.006344	-3.95688
C	-4.43422	-2.42019	1.87359
H	-5.30163	-3.01873	1.61338
C	-5.61536	-3.37246	4.545165
C	2.540101	-3.68453	2.177845
C	-0.30282	-2.48202	-0.10682
C	1.313198	-0.75307	-0.02208
C	-0.1048	4.620592	0.857336
H	0.603674	4.8507	1.643596
C	-2.62073	2.633614	-4.93619
H	-1.88085	3.27458	-5.42141
H	-3.22266	2.182396	-5.73295
H	-3.26756	3.236861	-4.29767

C	-0.44698	2.326105	1.874078
C	-3.16974	-4.56477	-1.51901
C	-3.6054	1.074177	-2.64193
C	2.112716	-1.51676	0.836279
H	3.065886	-1.12185	1.164253
C	1.306211	1.521048	5.221841
C	-4.17714	-3.70863	4.072239
C	0.802175	7.313383	-1.42378
H	1.192852	8.336491	-1.43275
H	1.643748	6.631444	-1.59752
H	0.108414	7.220976	-2.26639
C	-1.26608	0.249449	2.665953
C	2.179575	2.790364	5.233095
C	2.937739	-4.96899	1.405358
H	2.064542	-5.54595	1.081205
H	3.533329	-4.73169	0.516735
H	3.539301	-5.61773	2.051319
C	4.528248	3.100223	-0.83351
C	1.644763	0.560766	-0.6118
C	-0.43924	0.330946	3.786106
H	-0.48358	-0.45101	4.53578
C	-4.04939	-1.41419	0.996437
H	-4.60087	-1.2389	0.079578
C	1.698269	-4.0683	3.420498
H	0.785548	-4.61321	3.154658
H	2.285324	-4.71708	4.079339
H	1.411367	-3.17814	3.993786
C	-3.74822	-5.98364	-1.52719
C	-5.24644	-5.92406	-1.13729
H	-5.8302	-5.30096	-1.8233

H	-5.6759	-6.93115	-1.1646
H	-5.37828	-5.53073	-0.12185
C	0.11616	7.026737	-0.06448
C	0.392267	1.54249	6.474169
H	-0.28715	2.401869	6.455931
H	-0.21461	0.634519	6.560764
H	1.005489	1.614921	7.378977
C	1.146643	7.230577	1.062241
H	1.530328	8.254883	1.023472
H	0.704066	7.09049	2.055652
H	2.008183	6.558037	0.962293
C	4.715686	3.390919	0.676806
H	3.937746	4.067866	1.051657
H	4.696414	2.475597	1.278361
H	5.685969	3.872168	0.841497
C	-3.25664	-3.78157	5.305441
H	-3.60962	-4.57049	5.977012
H	-2.22097	-4.02449	5.035702
H	-3.2615	-2.84667	5.878318
C	5.647651	2.146854	-1.3233
H	6.622476	2.627806	-1.1842
H	5.663138	1.199389	-0.77549
H	5.534117	1.912949	-2.38687
C	-4.17325	-5.08851	3.368748
C	3.823262	-2.98049	2.656601
H	3.606585	-2.06833	3.225935
H	4.379204	-3.65104	3.319362
H	4.493615	-2.72057	1.829951
C	4.635481	4.427576	-1.60793
H	3.876021	5.154497	-1.29301

H	5.614371	4.880065	-1.42027
H	4.55061	4.28024	-2.69077
C	-1.05279	8.023802	0.146312
H	-0.66486	9.048049	0.163604
H	-1.79551	7.966302	-0.65653
H	-1.56685	7.839751	1.096276
C	0.962604	2.255417	-2.04579
H	0.199119	2.655607	-2.70038
C	-3.01749	-6.9173	-0.54369
H	-1.95861	-7.03961	-0.80124
H	-3.09098	-6.56479	0.492823
H	-3.47312	-7.9118	-0.58001
C	-3.60798	-6.56306	-2.95901
H	-2.55818	-6.60687	-3.26992
H	-4.01226	-7.58074	-2.98561
H	-4.15591	-5.97128	-3.69998
C	2.236718	0.284071	5.279689
H	2.907919	0.250849	4.412804
H	2.854475	0.326965	6.183205
H	1.674604	-0.65607	5.313037
H	-4.79949	1.185884	-1.10824
H	-5.65331	-2.39551	5.039713
H	-6.33029	-3.35859	3.715568
H	-5.95455	-4.12862	5.261519
H	2.85949	2.833024	4.372961
H	1.576301	3.705678	5.250911
H	2.798371	2.797588	6.135928
H	-4.51248	-5.86098	4.067099
H	-4.84454	-5.11266	2.503292
H	-3.16575	-5.36028	3.030307

I	-4.51888	2.469729	1.67454
I	0.52318	-1.59544	-3.83469
H	10.43042	-3.48806	-0.48325
H	8.714012	-3.73678	0.089699
C	8.981036	-1.92072	-1.17819
C	7.574455	-1.82898	-1.67054
C	9.429215	-1.62998	0.268842
H	9.706214	-1.59518	-1.92088
C	8.501443	-1.18689	1.360859
H	10.3946	-1.12598	0.310998
C	7.340641	-1.21381	-2.91728
C	6.052416	-1.13306	-3.46176
C	4.958009	-1.66506	-2.76565
C	5.176629	-2.27547	-1.52135
C	6.465421	-2.35655	-0.97785
H	8.183385	-0.81203	-3.47787
H	5.906713	-0.67373	-4.43769
H	3.957638	-1.62569	-3.1928
H	4.336824	-2.71936	-0.98754
H	6.609295	-2.84883	-0.01913
C	8.157433	-2.03599	2.427005
C	7.36916	-1.57319	3.491145
C	6.914501	-0.24697	3.50707
C	7.25223	0.609875	2.447107
C	8.035226	0.141437	1.384669
H	8.534763	-3.05663	2.439483
H	7.135207	-2.23912	4.320063
H	6.331772	0.122776	4.349213
H	6.929563	1.649863	2.464455
H	8.307533	0.813242	0.571957

Table S14. Optimized structure of **INT3A···*trans*-cyclopropane** at the B3LYP level

Atom	X	Y	Z
Ir	-0.10662	-0.61691	-1.29111
Ir	-1.60187	1.680701	-0.80863
C	6.876077	-3.91466	-0.9079
O	2.229764	-0.3746	-3.71302
H	2.009263	-1.1374	-3.08906
N	-1.3823	1.895494	1.143516
N	0.036142	2.955832	-0.74928
N	-1.77363	-1.79408	-1.71178
N	-0.40448	-1.43495	0.468493
N	-1.84007	1.519044	-2.84619
N	1.546525	0.124507	-0.24118
N	0.090825	0.327582	-3.277
N	-3.27453	0.642904	-0.1179
C	-3.40773	0.677692	1.246822
C	-3.18262	-3.51287	-0.76306
H	-3.46072	-4.08572	0.112876
C	3.748203	0.763645	1.456659
C	-2.34935	-2.05089	-2.9027
H	-1.9744	-1.48635	-3.74762
C	0.401604	3.315455	0.523148
C	2.025994	4.943011	-0.30807
C	-3.33893	-3.0098	-3.08288
H	-3.73099	-3.15763	-4.08444
C	-4.55603	0.189633	1.879963
H	-4.61817	0.253116	2.959352
C	0.640431	3.585912	-1.7752
H	0.292616	3.318976	-2.76656
C	-1.17448	2.354203	3.887104

C	-0.74808	-2.72922	2.916433
C	3.687262	1.18006	0.117833
H	4.491678	1.746506	-0.33748
C	1.196033	0.371724	-4.00325
C	-1.62448	-2.95521	1.834252
H	-2.4455	-3.65527	1.938463
C	-2.18592	-2.53975	-0.63816
C	-5.63393	-0.32658	1.146549
C	-0.26282	2.921034	2.979758
H	0.533379	3.560228	3.339702
C	1.617295	4.558568	-1.60183
H	2.034797	5.02842	-2.48709
C	2.661581	-0.01365	1.89975
H	2.655024	-0.40594	2.910485
C	-1.49307	0.955789	-5.19769
H	-1.13999	0.0481	-5.69533
H	-2.58457	0.985768	-5.26262
H	-1.11377	1.816155	-5.7567
C	-5.46826	-0.34014	-0.25357
H	-6.25108	-0.70399	-0.91172
C	-8.09906	0.080076	1.275333
C	-0.96026	-3.46966	4.244991
C	-1.43163	-2.31148	0.61262
C	0.482586	-1.20804	1.463303
C	1.387445	4.282046	0.751136
H	1.630822	4.541082	1.774237
C	1.487504	1.293855	-5.16194
H	2.563301	1.484486	-5.17182
H	1.241463	0.801544	-6.1095
H	0.948795	2.240124	-5.09987

C	-0.39027	2.69269	1.604602
C	-3.79115	-3.7862	-1.99698
C	-1.07126	0.987393	-3.74078
C	0.320376	-1.84264	2.700102
H	1.042176	-1.66124	3.485924
C	-1.09876	2.596759	5.401838
C	-6.93806	-0.80955	1.790874
C	4.3775	5.648798	-0.83987
H	5.123477	6.441675	-0.72001
H	4.800166	4.724523	-0.42762
H	4.225008	5.503664	-1.91479
C	-2.31504	1.362055	1.972385
C	0.112287	3.46487	5.794061
C	-0.86669	-4.99579	3.988077
H	-1.63207	-5.34677	3.287287
H	0.112877	-5.27264	3.582276
H	-1.00877	-5.53915	4.928509
C	4.913903	1.086694	2.396991
C	1.596193	-0.32647	1.058671
C	-2.21757	1.570321	3.347872
H	-2.96914	1.144653	4.003149
C	-4.30083	0.140728	-0.83271
H	-4.17969	0.147363	-1.91015
C	-2.36422	-3.1233	4.80098
H	-3.16598	-3.4079	4.110493
H	-2.53464	-3.66097	5.739855
H	-2.4543	-2.04962	5.00671
C	-4.84926	-4.87622	-2.19701
C	-6.13582	-4.23455	-2.77445
H	-5.95749	-3.73648	-3.73351

H	-6.89135	-5.00911	-2.94372
H	-6.55978	-3.49849	-2.08039
C	3.070315	6.046792	-0.10987
C	-2.39144	3.322004	5.856751
H	-2.50588	4.286208	5.349126
H	-3.29126	2.728627	5.660581
H	-2.35063	3.509891	6.935155
C	3.388517	6.287821	1.377986
H	4.147009	7.072013	1.465475
H	2.509196	6.627844	1.937965
H	3.791814	5.391037	1.865524
C	4.36716	1.78323	3.668106
H	3.867706	2.728602	3.421058
H	3.658111	1.153278	4.216948
H	5.194798	2.00986	4.34872
C	-6.8964	-0.72375	3.328481
H	-7.84256	-1.08988	3.739246
H	-6.09646	-1.34115	3.756621
H	-6.77185	0.307448	3.679675
C	5.612546	-0.23979	2.791041
H	6.449963	-0.02747	3.465042
H	4.937235	-0.93066	3.306984
H	6.003379	-0.75692	1.909499
C	-7.19443	-2.28178	1.383544
C	0.092382	-3.085	5.302294
H	0.061274	-2.0156	5.545494
H	-0.10598	-3.63332	6.228619
H	1.11008	-3.34352	4.986614
C	5.947839	2.015688	1.733671
H	5.507566	2.97771	1.441155

H	6.752749	2.229284	2.44454
H	6.404893	1.557449	0.850252
C	2.516627	7.361574	-0.71834
H	3.248181	8.165947	-0.5852
H	2.319577	7.268993	-1.79174
H	1.584869	7.665908	-0.22862
C	2.588986	0.851789	-0.67746
H	2.553484	1.176096	-1.70949
C	-5.20216	-5.59521	-0.88112
H	-4.33724	-6.11347	-0.44997
H	-5.61215	-4.90709	-0.13117
H	-5.96646	-6.35419	-1.07537
C	-4.29616	-5.92126	-3.20068
H	-3.37738	-6.38611	-2.82596
H	-5.03803	-6.71233	-3.35437
H	-4.07761	-5.47999	-4.17894
C	-0.98395	1.235096	6.131331
H	-0.07016	0.703749	5.838244
H	-0.94656	1.395907	7.214076
H	-1.84004	0.581962	5.927518
H	-2.67372	1.968428	-3.23215
H	-7.94259	1.131511	1.540734
H	-8.21399	0.020127	0.187798
H	-9.04249	-0.24662	1.726107
H	1.064709	3.0021	5.506478
H	0.060707	4.46742	5.353261
H	0.130214	3.593722	6.880821
H	-8.13598	-2.62859	1.822488
H	-7.27633	-2.40154	0.297639
H	-6.3936	-2.93979	1.742935

I	-3.27398	3.973173	-0.97729
I	1.430004	-3.04856	-1.95827
H	7.518244	-4.66247	-0.45076
H	6.703595	-4.05071	-1.97213
C	6.972803	-2.49251	-0.3906
C	6.894586	-1.31334	-1.3033
C	5.741313	-3.35591	-0.07544
H	7.657527	-2.35519	0.444314
C	5.454763	-3.84091	1.307848
H	4.84711	-3.0905	-0.63588
C	7.750469	-0.21549	-1.08569
C	7.727123	0.902549	-1.93107
C	6.838859	0.948152	-3.01544
C	5.980644	-0.13942	-3.24272
C	6.007139	-1.25534	-2.39723
H	8.461699	-0.24798	-0.26123
H	8.415921	1.726838	-1.75535
H	6.837497	1.803519	-3.68815
H	5.301991	-0.13026	-4.09404
H	5.341219	-2.09078	-2.60632
C	4.115039	-3.96766	1.723841
C	3.800465	-4.43026	3.009156
C	4.819367	-4.76984	3.910285
C	6.157578	-4.64824	3.505488
C	6.470569	-4.19172	2.219277
H	3.316331	-3.72873	1.021882
H	2.75681	-4.55056	3.298719
H	4.579384	-5.14046	4.904572
H	6.957813	-4.92096	4.19045
H	7.516957	-4.12175	1.927253

Table S15. Optimized structure of **1B…*cis*-cyclopropane** at the B3LYP level

Atom	X	Y	Z
C	8.070921	0.092587	1.33961
C	8.557992	-1.22671	1.398723
C	8.239906	-2.00858	2.522676
C	7.456739	-1.48824	3.563637
C	6.981701	-0.17078	3.497009
C	7.292504	0.61837	2.378186
C	9.473334	-1.73408	0.323932
C	9.011315	-2.06564	-1.10828
C	7.605027	-1.93875	-1.59771
C	7.383981	-1.32006	-2.84443
C	6.0944	-1.19574	-3.37819
C	4.987764	-1.68751	-2.67213
C	5.195378	-2.30225	-1.42836
C	6.484956	-2.42679	-0.89519
C	9.464825	-3.16259	-0.15715
C	3.768804	-2.87261	2.803171
C	2.504184	-3.59204	2.297785
C	1.635815	-3.96964	3.523566
C	1.677745	-2.71778	1.341376
C	0.472576	-3.21427	0.803221
C	-0.28516	-2.44303	-0.07741
N	0.128833	-1.19082	-0.4007
C	1.301543	-0.68702	0.048629
C	2.08598	-1.43553	0.934492
Ir	-0.92857	-0.15936	-1.69509
I	0.645727	-1.48742	-3.75665
C	-1.50659	-2.85476	-0.80182
N	-2.05524	-1.89278	-1.60905

C	-3.12114	-2.23183	-2.35837
C	-3.67772	-3.50539	-2.34852
C	-3.13363	-4.52069	-1.53643
C	-2.03366	-4.15008	-0.75125
C	1.632954	0.620821	-0.55658
N	0.691014	1.11682	-1.42992
C	0.987989	2.239918	-2.10447
C	2.191094	2.927424	-1.93124
C	3.162785	2.463184	-1.03248
C	2.84494	1.276508	-0.34612
C	4.513383	3.155999	-0.81064
C	4.647771	4.442755	-1.64697
C	-3.71092	-5.94128	-1.56732
C	-3.56093	-6.50316	-3.00426
Ir	-2.51068	1.131231	0.172023
I	-4.57005	2.50378	1.633721
N	-2.22019	0.861765	-3.08744
C	-3.45917	1.220262	-2.7231
C	-4.47754	1.737442	-3.73016
N	-1.29201	1.210945	1.716679
C	-0.51671	2.310096	1.88199
C	0.293913	2.425003	3.01749
C	0.289677	1.42432	4.00402
C	-0.57046	0.323025	3.80517
C	-1.36554	0.237857	2.662356
C	1.134598	1.517152	5.284612
C	2.061488	0.280634	5.380577
C	-0.71312	3.317097	0.818928
N	-1.6009	2.961582	-0.16547
C	-1.88109	3.870768	-1.11789

C	-1.31755	5.140891	-1.14071
C	-0.40715	5.541231	-0.14269
C	-0.11367	4.581854	0.835715
C	0.179375	6.958203	-0.14055
C	-0.98289	7.973091	0.00784
C	-2.40308	-0.77226	2.362609
N	-3.07656	-0.58641	1.181152
C	-4.11941	-1.39813	0.916634
C	-4.52583	-2.41132	1.775924
C	-3.84583	-2.64036	2.990001
C	-2.76365	-1.79081	3.252867
C	-4.31596	-3.73058	3.961346
C	-4.29128	-5.10226	3.243673
N	-3.81757	1.141065	-1.44822
C	-1.7572	1.229201	-4.39544
C	-2.17204	0.368387	-5.5689
O	-1.00013	2.179101	-4.52888
C	2.935966	-4.88191	1.554861
C	2.011452	2.783952	5.315356
C	0.187285	1.549857	6.510837
C	-5.76489	-3.40653	4.406243
C	-3.42609	-3.81894	5.216054
C	4.655898	3.525462	0.686755
C	5.649775	2.184295	-1.21595
C	-5.21093	-5.89441	-1.18545
C	-2.98512	-6.88848	-0.5929
C	0.916385	7.21054	-1.47889
C	1.172513	7.177701	1.016572
H	-1.55174	-4.87977	-0.11212
H	-3.51108	-1.4496	-2.9986

H	-4.52611	-3.69602	-2.99888
H	-2.20678	-1.88906	4.176756
H	-2.59359	3.561766	-1.87245
H	2.351833	3.817137	-2.52949
H	0.133315	-4.2125	1.056716
H	0.91073	3.306581	3.138692
H	-1.61107	5.812185	-1.9419
H	3.563223	0.831908	0.334543
H	-4.93649	0.912222	-4.2864
H	-5.27513	2.280914	-3.2165
H	-4.01912	2.413133	-4.45829
H	-5.39005	-3.00384	1.491408
H	0.568724	4.819074	1.642841
H	-1.95739	0.88962	-6.50453
H	-1.56846	-0.54675	-5.524
H	-3.22484	0.077096	-5.52268
H	3.022732	-1.0237	1.288462
H	1.320365	8.229047	-1.49622
H	1.752471	6.512835	-1.60848
H	0.252328	7.104239	-2.34301
H	2.076736	-5.46896	1.21245
H	3.55109	-4.64787	0.678673
H	3.526439	-5.5186	2.223652
H	-0.63825	-0.45613	4.556574
H	-4.64431	-1.19651	-0.01076
H	0.734315	-4.52307	3.238161
H	2.209824	-4.60525	4.207335
H	1.324543	-3.07409	4.075039
H	-5.79097	-5.26121	-1.86511
H	-5.63887	-6.90228	-1.22811

H	-5.34796	-5.51262	-0.16645
H	-0.48928	2.41053	6.464512
H	-0.42629	0.645141	6.582296
H	0.772705	1.625924	7.434331
H	1.57098	8.196526	0.967943
H	0.693525	7.063316	1.996308
H	2.026381	6.490514	0.962337
H	3.86688	4.220478	0.999983
H	4.608641	2.642309	1.333544
H	5.622317	4.012769	0.860574
H	-3.79419	-4.61666	5.869743
H	-2.38405	-4.0556	4.966868
H	-3.44422	-2.88964	5.797582
H	6.621406	2.671572	-1.07062
H	5.645061	1.264477	-0.62251
H	5.567622	1.896261	-2.26924
H	3.527246	-1.95749	3.357206
H	4.314852	-3.53205	3.485973
H	4.455191	-2.6115	1.99008
H	3.876369	5.181058	-1.39548
H	5.62045	4.906351	-1.45057
H	4.594859	4.239523	-2.72254
H	-0.58688	8.995082	0.013815
H	-1.69916	7.898995	-0.81722
H	-1.53084	7.813895	0.943436
H	0.247932	2.565842	-2.82926
H	-1.92387	-7.00121	-0.84452
H	-3.06525	-6.54875	0.447153
H	-3.43786	-7.88415	-0.64629
H	-2.50854	-6.5376	-3.30768

H	-3.96299	-7.52173	-3.05002
H	-4.10254	-5.89893	-3.73973
H	2.755577	0.240883	4.532386
H	2.653706	0.326695	6.301596
H	1.496267	-0.65787	5.399975
H	-4.76096	1.470681	-1.26406
H	-5.81566	-2.43523	4.910835
H	-6.45839	-3.38018	3.559058
H	-6.12119	-4.1728	5.104235
H	2.71456	2.819445	4.474039
H	1.409569	3.700214	5.306434
H	2.603588	2.795507	6.236488
H	-4.64466	-5.88624	3.92288
H	-4.93924	-5.11494	2.360614
H	-3.27509	-5.36408	2.924309
H	10.41394	-3.65169	-0.35951
H	8.692551	-3.82278	0.228845
H	9.74374	-1.79101	-1.86473
H	10.45441	-1.25961	0.34898
H	8.235766	-0.94523	-3.4106
H	5.953935	-0.72842	-4.3511
H	3.983919	-1.60629	-3.0849
H	4.342549	-2.70451	-0.88323
H	6.617974	-2.91632	0.066809
H	8.62608	-3.02377	2.592387
H	7.234498	-2.1038	4.433709
H	6.392823	0.241802	4.31451
H	6.94165	1.647751	2.323817
H	8.31228	0.708908	0.475098

Table S16. Optimized structure of **1B…*trans*-cyclopropane** at the B3LYP level

Atom	X	Y	Z
C	6.916928	-4.12723	1.951457
C	5.784629	-3.71746	1.219399
C	4.513537	-3.91659	1.793306
C	4.37837	-4.50359	3.058693
C	5.51252	-4.90109	3.781124
C	6.783382	-4.71015	3.217789
C	5.876778	-3.09979	-0.1377
C	7.062622	-2.2154	-0.54228
C	6.888258	-0.95904	-1.33194
C	7.836771	0.073656	-1.19304
C	7.72977	1.259931	-1.93231
C	6.662645	1.43955	-2.82482
C	5.707567	0.420604	-2.96903
C	5.820352	-0.76464	-2.23124
C	6.874047	-3.58423	-1.17067
O	2.328529	0.45948	-3.76692
C	1.275946	-0.11332	-4.01003
C	1.183266	-1.17638	-5.0844
N	0.114657	0.155487	-3.21586
C	-0.85859	0.918718	-3.73441
C	-0.88894	1.297848	-5.20897
Ir	-0.06212	-0.61985	-1.21071
I	1.646074	-2.93846	-1.69519
Ir	-1.69038	1.589743	-0.87843
I	-3.43591	3.852608	-1.16106
N	-1.66158	-1.86955	-1.61956
C	-2.21215	-2.15573	-2.81437
C	-3.15952	-3.15731	-2.99069

C	-3.59208	-3.93695	-1.8992
C	-3.02043	-3.62029	-0.65954
C	-2.0641	-2.60616	-0.53626
C	-1.35424	-2.3048	0.725095
N	-0.36987	-1.38147	0.573739
C	0.47986	-1.07209	1.579747
C	0.31213	-1.66283	2.837712
C	-0.7153	-2.59537	3.059252
C	-1.54817	-2.91127	1.965889
C	-0.92663	-3.29599	4.410522
C	0.083505	-2.82849	5.475921
C	1.570568	-0.17014	1.154837
N	1.533191	0.214006	-0.16657
C	2.572098	0.919642	-0.64395
C	3.65715	1.299988	0.149527
C	3.706519	0.959486	1.50924
C	2.622682	0.201357	1.989295
C	4.87351	1.323621	2.435561
C	5.910376	2.220018	1.732858
C	-4.59779	-5.07705	-2.10102
C	-3.97785	-6.11659	-3.06958
N	-1.54519	1.874157	1.064756
C	-0.59703	2.721785	1.532433
C	-0.52652	2.998194	2.902988
C	-1.4485	2.426928	3.79652
C	-2.44507	1.589351	3.250843
C	-2.48834	1.334282	1.880317
C	-1.43226	2.717983	5.305631
C	-1.28713	1.387137	6.083869
C	0.218704	3.321802	0.456535

N	-0.08387	2.895823	-0.81245
C	0.572469	3.465661	-1.84032
C	1.528662	4.459592	-1.66951
C	1.860216	4.924352	-0.38147
C	1.17984	4.314068	0.680904
C	2.88022	6.054029	-0.19453
C	2.335746	7.327956	-0.89001
C	-3.5272	0.589285	1.136887
N	-3.343	0.526776	-0.22178
C	-4.3208	-0.02821	-0.96475
C	-5.48824	-0.54318	-0.41506
C	-5.70364	-0.50754	0.978149
C	-4.67892	0.069269	1.7394
C	-7.00989	-1.03402	1.586103
C	-7.1862	-2.52565	1.210446
N	-1.81917	1.381263	-2.94581
C	-0.75982	-4.82458	4.217668
C	-2.3557	-2.99286	4.925032
C	-0.26944	3.645043	5.709365
C	-2.76405	3.405121	5.700569
C	-8.19114	-0.21311	1.008867
C	-7.03145	-0.91052	3.12174
C	4.332511	2.076183	3.676063
C	5.573744	0.016225	2.885736
C	-5.89972	-4.50934	-2.71823
C	-4.94913	-5.7873	-0.77981
C	4.224993	5.645915	-0.84493
C	3.130188	6.375029	1.291427
H	-3.29213	-4.18482	0.223991
H	-1.85234	-1.56646	-3.64911

H	-3.53737	-3.33081	-3.99378
H	-4.78179	0.154552	2.81434
H	0.300702	3.114937	-2.82819
H	4.465228	1.838953	-0.33173
H	-2.33455	-3.64971	2.075766
H	0.236706	3.674721	3.266525
H	1.998802	4.870226	-2.55773
H	2.610177	-0.13674	3.019914
H	-1.30539	0.486573	-5.81675
H	-1.51343	2.182803	-5.35829
H	0.111723	1.518535	-5.59186
H	-6.23182	-0.95281	-1.09194
H	1.37246	4.625143	1.700413
H	1.285768	-2.14455	-4.57767
H	0.222605	-1.17156	-5.60518
H	2.006769	-1.0617	-5.79319
H	1.00119	-1.41151	3.633983
H	4.954592	6.455539	-0.73058
H	4.639198	4.747618	-0.37224
H	4.121632	5.444504	-1.91626
H	-1.49155	-5.23485	3.513314
H	0.239834	-5.06881	3.840902
H	-0.89822	-5.33973	5.175079
H	-3.20282	1.155862	3.894436
H	-4.14985	-0.02913	-2.03574
H	-3.12657	-3.33572	4.226022
H	-2.52635	-3.50194	5.88038
H	-2.49622	-1.91689	5.084726
H	-5.72135	-4.02005	-3.68164
H	-6.61689	-5.3202	-2.88821

H	-6.36921	-3.77862	-2.04852
H	-2.89879	4.347128	5.157337
H	-3.63301	2.771239	5.492613
H	-2.76653	3.627467	6.773799
H	3.872914	7.17561	1.372267
H	2.221069	6.724727	1.795102
H	3.522906	5.509064	1.839148
H	3.83485	3.010469	3.38715
H	3.618798	1.473684	4.249268
H	5.160376	2.329563	4.347866
H	-7.97598	-1.30859	3.507135
H	-6.22058	-1.48012	3.592889
H	-6.9622	0.133299	3.449985
H	6.414766	0.254025	3.547532
H	4.897228	-0.64964	3.432552
H	5.960647	-0.54019	2.025891
H	0.002513	-1.75302	5.675794
H	-0.11237	-3.35053	6.418415
H	1.117514	-3.05368	5.189115
H	5.470161	3.166533	1.394587
H	6.714737	2.465238	2.435043
H	6.367169	1.722804	0.870745
H	3.048895	8.151586	-0.77017
H	2.182304	7.174242	-1.9634
H	1.379019	7.638763	-0.45518
H	2.542098	1.144852	-1.70529
H	-4.07057	-6.25424	-0.3189
H	-5.40484	-5.10294	-0.05353
H	-5.67391	-6.58451	-0.97546
H	-3.04749	-6.5298	-2.66405

H	-4.67911	-6.94417	-3.22621
H	-3.75309	-5.68191	-4.04934
H	-0.34311	0.886605	5.83658
H	-1.29477	1.580568	7.16251
H	-2.10637	0.691991	5.868424
H	-2.48414	1.987217	-3.41844
H	-8.08945	0.850529	1.251606
H	-8.26006	-0.30276	-0.08044
H	-9.13645	-0.57099	1.432668
H	0.70818	3.211454	5.465105
H	-0.34428	4.629105	5.231942
H	-0.29226	3.808534	6.791907
H	-8.12673	-2.90669	1.623964
H	-7.21927	-2.67449	0.12575
H	-6.36804	-3.13517	1.613328
H	7.560777	-4.37471	-0.87933
H	6.547249	-3.6163	-2.20665
H	7.859431	-2.15797	0.197276
H	4.914708	-2.7911	-0.54023
H	8.676565	-0.05995	-0.51208
H	8.48359	2.037143	-1.81834
H	6.583639	2.354263	-3.40938
H	4.868769	0.542907	-3.6508
H	5.062385	-1.53483	-2.36196
H	3.626568	-3.62864	1.22976
H	3.384828	-4.66513	3.475314
H	5.410399	-5.36218	4.761438
H	7.671321	-5.02252	3.764294
H	7.914099	-4.00035	1.533206

Table S17. Optimized structure of **1B** at the B3LYP level

Atom	X	Y	Z
C	2.786677	-2.137130	0.475914
N	1.612570	-1.579409	0.817036
C	1.514814	-0.991696	2.057715
C	2.606473	-0.937420	2.923019
C	3.844222	-1.509543	2.577517
C	3.901709	-2.121797	1.316561
Ir	-0.211374	-1.697836	-0.17363
N	0.237562	-2.638061	-2.060647
C	0.013461	-1.985543	-3.209788
C	0.096677	-2.682368	-4.561018
C	5.021059	-1.476843	3.561516
C	6.272014	-2.172236	2.991656
C	0.160166	-0.533033	2.432196
N	-0.767828	-0.810513	1.487694
C	-2.093623	-0.612738	1.705297
C	-2.518033	-0.034720	2.901193
C	-1.587445	0.319523	3.900154
C	-0.232777	0.048870	3.643051
C	-2.078313	0.933134	5.220698
C	-0.919324	1.247943	6.185939
C	-2.944527	-1.139670	0.615570
N	-2.259900	-1.652668	-0.460322
C	-2.973642	-2.217453	-1.447085
C	-4.363974	-2.321358	-1.417812
C	-5.093275	-1.830385	-0.32282
C	-4.335400	-1.214632	0.689919
C	-6.613254	-1.987500	-0.183396
C	-6.905829	-2.858029	1.065670

Ir	0.200657	0.614074	-1.632293
I	0.507228	2.640756	-3.646820
I	-0.633782	-4.199400	1.256915
N	0.729355	1.872065	-0.212414
C	2.045676	2.019923	0.074612
C	2.448673	2.965141	1.025447
C	1.503256	3.784445	1.665495
C	0.151222	3.623984	1.293662
C	-0.217601	2.673306	0.342086
C	1.893894	4.855956	2.695789
C	1.155801	4.580297	4.028809
C	2.915123	1.169407	-0.763481
N	2.255972	0.353358	-1.648693
C	2.998552	-0.385048	-2.494798
C	4.387868	-0.352437	-2.508562
C	5.096329	0.476793	-1.616601
C	4.313095	1.232668	-0.734115
C	6.627664	0.546787	-1.660203
C	7.058499	1.070277	-3.054157
C	-1.557333	2.465456	-0.24736
N	-1.634438	1.473251	-1.192719
C	-2.801328	1.321753	-1.850394
C	-3.912469	2.122402	-1.61451
C	-3.866072	3.151813	-0.651464
C	-2.654186	3.287255	0.037400
C	-5.063370	4.086468	-0.436955
C	-6.310368	3.253452	-0.055333
N	-0.276039	-0.690987	-3.183338
C	0.836177	-3.941329	-2.116231
C	-0.054847	-5.126132	-2.418976

O	2.023621	-4.076892	-1.860935
C	-3.034359	-0.068195	5.917233
C	-2.836486	2.250082	4.921375
C	3.409291	4.873266	2.974925
C	1.481568	6.248588	2.154936
C	-5.334962	4.845792	-1.761045
C	-4.805832	5.119972	0.676555
C	5.377638	-0.003819	3.881184
C	4.607583	-2.204364	4.865897
C	-7.239538	-2.671366	-1.414086
C	-7.267538	-0.596167	-0.008977
C	7.212203	-0.869883	-1.437772
C	7.200277	1.489167	-0.584306
H	-4.827124	-0.822093	1.573662
H	-2.397787	-2.623845	-2.269905
H	-4.855540	-2.813763	-2.249353
H	-2.534494	4.067992	0.778582
H	2.447775	-1.007538	-3.188585
H	4.801146	-2.615919	0.966982
H	-3.578613	0.120723	3.065427
H	3.502632	3.072864	1.248849
H	4.902888	-0.975094	-3.233623
H	2.473501	-0.472614	3.894181
H	-0.813966	-3.256792	-4.765461
H	0.216127	-1.945895	-5.360186
H	0.942100	-3.375297	-4.606939
H	-4.802880	1.946225	-2.210402
H	4.784598	1.903846	-0.026619
H	-0.616485	-5.344572	-1.501922
H	-0.777755	-4.915692	-3.211689

H	0.557036	-5.993959	-2.675208
H	0.517842	0.268336	4.391754
H	8.306235	-0.834644	-1.489618
H	6.932817	-1.263540	-0.452976
H	6.868711	-1.582502	-2.194809
H	-3.916273	-0.290154	5.306573
H	-2.527401	-1.015990	6.129940
H	-3.387095	0.350424	6.866729
H	-0.611582	4.259399	1.730296
H	-2.813362	0.544717	-2.607095
H	-3.698980	2.089302	4.265154
H	-3.208700	2.688888	5.854055
H	-2.177037	2.983385	4.441387
H	-6.853358	-3.686627	-1.56036
H	-8.322576	-2.753202	-1.274114
H	-7.070889	-2.099040	-2.334287
H	1.980958	6.466752	1.204343
H	0.401095	6.323985	1.990572
H	1.761572	7.027331	2.873578
H	8.293403	1.496061	-0.648722
H	6.860956	2.522899	-0.720753
H	6.935358	1.165090	0.430164
H	5.686799	0.533196	2.975944
H	4.534144	0.536962	4.325103
H	6.207365	0.034246	4.596131
H	-5.691480	5.752126	0.799598
H	-4.608386	4.642539	1.644568
H	-3.966477	5.782924	0.435515
H	5.441766	-2.198801	5.576822
H	3.753257	-1.722942	5.354501

H	4.336424	-3.247049	4.666850
H	-0.211107	1.967934	5.757758
H	-1.319344	1.691213	7.103907
H	-0.369397	0.344760	6.475467
H	6.628545	-1.689769	2.073436
H	7.084637	-2.121389	3.724125
H	6.090278	-3.231556	2.777815
H	8.151552	1.129977	-3.108578
H	6.721032	0.411553	-3.861252
H	6.652153	2.070044	-3.244685
H	2.808294	-2.643005	-0.484883
H	-6.883721	-0.064245	0.868487
H	-7.095307	0.034195	-0.889105
H	-8.350035	-0.707190	0.120180
H	-6.532297	-2.395987	1.986244
H	-7.987289	-2.996123	1.178206
H	-6.443267	-3.847158	0.975778
H	1.441131	3.605751	4.443282
H	1.412235	5.349577	4.765964
H	0.066886	4.591292	3.907075
H	-0.389831	-0.275456	-4.103651
H	-4.463799	5.437451	-2.063871
H	-5.577830	4.163328	-2.582441
H	-6.183319	5.527801	-1.63264
H	3.767061	3.914942	3.371322
H	3.989243	5.118263	2.077420
H	3.633588	5.640034	3.723865
H	-7.174016	3.914806	0.077104
H	-6.570173	2.523746	-0.829107
H	-6.153093	2.709692	0.883947

References

(1) Frisch, M. J.; Trucks, G. W.; Schlegel, H. B.; Scuseria, G. E.; Robb, M. A.; Cheeseman, J. R.; Scalmani, G.; Barone, V.; Petersson, G. A.; Nakatsuji, H.; Li, X.; Caricato, M.; Marenich, A. V.; Bloino, J.; Janesko, B. G.; Gomperts, R.; Mennucci, B.; Hratchian, H. P.; Ortiz, J. V.; Izmaylov, A. F.; Sonnenberg, J. L.; Williams-Young, D.; Ding, F.; Lipparini, F.; Egidi, F.; Goings, J.; Peng, B.; Petrone, A.; Henderson, T.; Ranasinghe, D.; Zakrzewski, V. G.; Gao, J.; Rega, N.; Zheng, G.; Liang, W.; Hada, M.; Ehara, M.; Toyota, K.; Fukuda, R.; Hasegawa, J.; Ishida, M.; Nakajima, T.; Honda, Y.; Kitao, O.; Nakai, H.; Vreven, T.; Throssell, K.; Montgomery Jr., J. A.; Peralta, J. E.; Ogliaro, F.; Bearpark, M. J.; Heyd, J. J.; Brothers, E. N.; Kudin, K. N.; Staroverov, V. N.; Keith, T. A.; Kobayashi, R.; Normand, J.; Raghavachari, K.; Rendell, A. P.; Burant, J. C.; Iyengar, S. S.; Tomasi, J.; Cossi, M.; Millam, J. M.; Klene, M.; Adamo, C.; Cammi, R.; Ochterski, J. W.; Martin, R. L.; Morokuma, K.; Farkas, O.; Foresman, J. B.; Fox, D. J. Gaussian 16, Revision C.01 (Gaussian, Inc., Wallingford, CT, 2016).

(2) Becke, A. D. Density-functional exchange-energy approximation with correct asymptotic behavior. *Phys. Rev. A: At., Mol., Opt. Phys.* **1988**, *38,* 3098−3100, DOI: 10.1103/PhysRevA.38.3098.

(3) Lee, C.; Yang, W.; Parr, R. G. Development of the Colle-Salvetti correlation-energy formula into a functional of the electron density. *Phys. Rev. B: Condens. Matter Mater. Phys.* **1988**, *37*, 785−789, DOI: 10.1103/PhysRevB.37.785.

(4) Becke, A. D. Density-functional thermochemistry. III. The role of exact exchange. *J. Chem. Phys.* **1993**, *98*, 5648−5652, DOI: 10.1063/1.464913.

(5) Andrae, D.; Häußermann, U.; Dolg, M.; Stoll, H.; Preuß, H. Energy-adjusted *ab initio* pseudopotentials for the second and third row transition-elements. *Theor. Chem. Acc.* **1990**, *77,* 123−141, DOI: 10.1007/BF01114537.

(6) Dunning, T. H.; Hay, P. J. Gaussian basis sets for molecular calculations, in *Methods of Electronic Structure Theory*, vol. 3 of *Modern Theoretical Chemistry*, H. F. Schaefer, Ed. (Springer, 1977), chap. 1, pp. 1–27, DOI: 10.1007/978-1-4757-0887-5_1.

(7) Doering, W. von E.; Hoffmann, A. K. The Addition of Dichlorocarbene to Olefins. *J. Am. Chem. Soc.* **1954**, *76*, 6162–6165, DOI: 10.1021/ja01652a087.

(8) Simmons, H. E.; Smith, R. D. A New Synthesis of Cyclopropanes from Olefines. *J. Am. Chem. Soc.* **1958**, *80*, 5323–5324, DOI: 10.1021/ja01552a080.

(9) Furukawa, J.; Kawabata, N.; Nishimura, J. Syntheshis of Cyclopropanes by the Reaction of Olefins with Dialkylzinc and Methylene Iodide. *Tetrahedron* **1968**, *24*, 53–58, DOI: 10.1016/0040-4020(68)89007-6.

(10) Kanai, H.; Hiraki, N. Cyclopropanation of Electron-Deficient Olefines with gem-Dibromides Catalyzed by Nickel Catalysts. *Chem. Lett.* **1979**, 761–762, DOI: 10.1246/cl.1979.761.

(11) Takai, K.; Toshikawa, S.; Inoue, A.; Kokumai, R. Stereoselective Iodocyclopropanation of Terminal Alkenes with Iodoform, Chromium(II) Chloride, and N,N,N',N'-Tetraethylethylenediamine. *J. Am. Chem. Soc.* **2003**, *125*, 12990–12991, DOI: 10.1021/ja0373061.

(12) Lorenz, J. C.; Long, J.; Yang, Z.; Xue, S.; Xie, Y.; Shi, Y. A Novel Class of Tunable Zinc Reagents ($RXZnCH_2Y$) for Efficient Cyclopropanation of Olefins. *J. Org. Chem.* **2004**, *69*, 327–334, DOI: 10.1021/jo030312v.

(13) Zhou, Y.-Y.; Uyeda, C. Reductive Cyclopropanations Catalyzed by Dinuclear Nickel Complexes. *Angew. Chem. Int. Ed.* **2016**, *55*, 3171–3175, DOI: 10.1002/anie.201511271.

(14) del Hoyo, A. M.; Herraiz, A. G.; Suero, M. G. A Stereoconvergent Cyclopropanation Reaction of Styrenes. *Angew. Chem. Int. Ed.* **2017**, *56*, 1610–1613, DOI: 10.1002/anie.201610924.

(15) Werth, J.; Uyeda, C. Regioselective Simmons–Smith-Type Cyclopropanations of Polyalkenes Enabled by Transition Metal Catalysis. *Chem. Sci.* **2018**, *9*, 1604–1609, DOI: 10.1039/c7sc04861k.

(16) Werth, J.; Uyeda, C. Cobalt-Catalyzed Reductive Dimethylcyclopropanation of 1,3-Dienes. *Angew. Chem. Int. Ed.* **2018**, *57*, 13902–13906, DOI: 10.1002/anie.201807542.

(17) Peil, S.; Guthertz, A.; Biberger, T.; Fürstner, A. Hydrogenative Cyclopropanation and Hydrogenative Metathesis. *Angew. Chem. Int. Ed.* **2019**, *58*, 8851–8856, DOI: 10.1002/anie.201904256.

(18) Ikeda, H.; Nishi, K.; Tsurugi, H.; Mashima, K. Chromium-Catalyzed Cyclopropanation of Alkenes with Bromoform in the presence of 2,3,5,6-Tetramethyl-1,4-bis(trimethylsilyl)-1,4-dihydropyrazine. *Chem. Sci.* **2020**, *11*, 3604–3609, DOI: 10.1039/d0sc00964d.

(19) Wei, N.; Yang, D.; Zhao, J.; Mei, T.; Zhang, Y.; Wang, B.; Qu, J. Structure and Methylene Transfer Reactivity of Thiolate-Bridged Dichromium Methylene Complexes Derived from Dihalomethane via Cleavage of Two Carbon–Halogen Bonds. *Organometallics* **2021**, *40*, 1434–1442, DOI: 10.1021/acs.organomet.1c00031.
